# A New Centrosaurine Ceratopsid, *Machairoceratops cronusi* gen et sp. nov., from the Upper Sand Member of the Wahweap Formation (Middle Campanian), Southern Utah

**DOI:** 10.1371/journal.pone.0154403

**Published:** 2016-05-18

**Authors:** Eric K. Lund, Patrick M. O’Connor, Mark A. Loewen, Zubair A. Jinnah

**Affiliations:** 1 Department of Biomedical Sciences, Ohio University Heritage College of Osteopathic Medicine, Athens, Ohio, United States of America; 2 Ohio Center for Ecology and Evolutionary Studies, Ohio University, Athens, Ohio, United States of America; 3 Department of Geology and Geophysics, University of Utah, Salt Lake City, Utah, United States of America; 4 Natural History Museum of Utah, Salt Lake City, Utah, United States of America; 5 School of Geosciences, University of the Witwatersrand, Johannesburg South Africa; Perot Museum of Nature and Science, UNITED STATES

## Abstract

The Upper Cretaceous (middle-late Campanian) Wahweap Formation of southern Utah contains the oldest diagnostic evidence of ceratopsids (to date, all centrosaurines) in North America, with a number of specimens recovered from throughout a unit that spans between 81 and 77 Ma. Only a single specimen has been formally named, *Diabloceratops eatoni*, from the lower middle member of the formation. *Machairoceratops cronusi* gen. et sp. nov., a new centrosaurine ceratopsid from the upper member of the Wahweap Formation, is here described based on cranial material representing a single individual recovered from a calcareous mudstone. The specimen consists of two curved and elongate orbital horncores, a left jugal, a nearly complete, slightly deformed braincase, the left squamosal, and a mostly complete parietal ornamented by posteriorly projected, anterodorsally curved, elongate spikes on either side of a midline embayment. The fan-shaped, stepped-squamosal is diagnostic of Centrosaurinae, however, this element differs from the rectangular squamosal in *Diabloceratops*. *Machairoceratops* also differs in the possession of two anterodorsally (rather than laterally) curved epiparietal ornamentations on either side of a midline embayment that are distinguished by a posteromedially-oriented sulcus along the entire length of the epiparietal. Additionally, the parietosquamosal frill is lacking any other epiossifications along its periphery. *Machairoceratops* shares a triangular (rather than round) frill and spike-like epiparietal loci (p1) ornamentation with the stratigraphically lower *Diabloceratops*. Both parsimony and Bayesian phylogenetic analyses place *Machairoceratops* as an early-branching centrosaurine. However, the parsimony-based analysis provides little resolution for the position of the new taxon, placing it in an unresolved polytomy with *Diabloceratops*. The resultant Bayesian topology yielded better resolution, aligning *Machairoceratops* as the definitive sister taxon to a clade formed by *Diabloceratops* and *Albertaceratops*. Considered together, both phylogenetic methods unequivocally place *Machairoceratops* as an early-branching centrosaurine, and given the biostratigraphic position of *Machairoceratops*, these details increase the known ceratopsid diversity from both the Wahweap Formation and the southern portion of Laramidia. Finally, the unique morphology of the parietal ornamentation highlights the evolutionary disparity of frill ornamentation near the base of Centrosaurinae.

## Introduction

**Fig 1:**
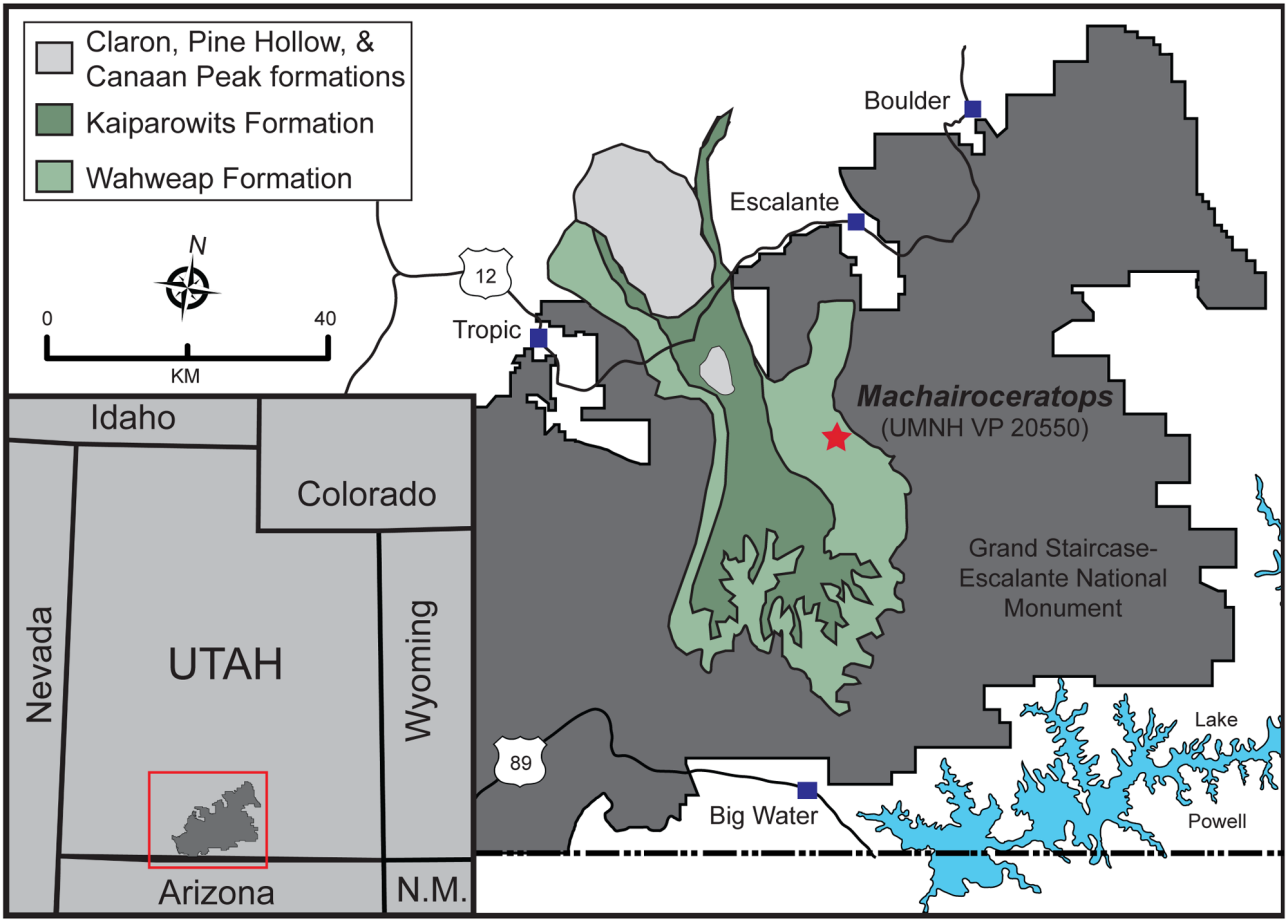
Locality map: Grand Staircase-Escalante National Monument, southern Utah. Map showing locality (indicated by star) of *Machairoceratops cronusi* gen et. sp nov. (UMNH VP 20550), recovered from the Wahweap Formation of Grand Staircase-Escalante National Monument (GSENM). GSENM is bounded by the red rectangle and silhouetted in dark gray on the inset of Utah and surrounding states (modified from [1]).

The centrosaurine fossil record from southern Laramidia (Utah, Colorado, New Mexico, Texas, and Mexico) has been scant relative to northern Laramidia (Alaska, Alberta, Saskatchewan, and Montana), resulting in a latitudinal bias of the dinosaur fossil record within the Western Interior Basin (WIB). However, new discoveries from the late Campanian Wahweap Formation in Grand Staircase-Escalante National Monument (GSENM), southern Utah are helping to expand both the temporal and geographic sampling of non-avian dinosaurian diversity, particularly the ceratopsid diversity in the WIB (Fig 1).

**Fig 2:**
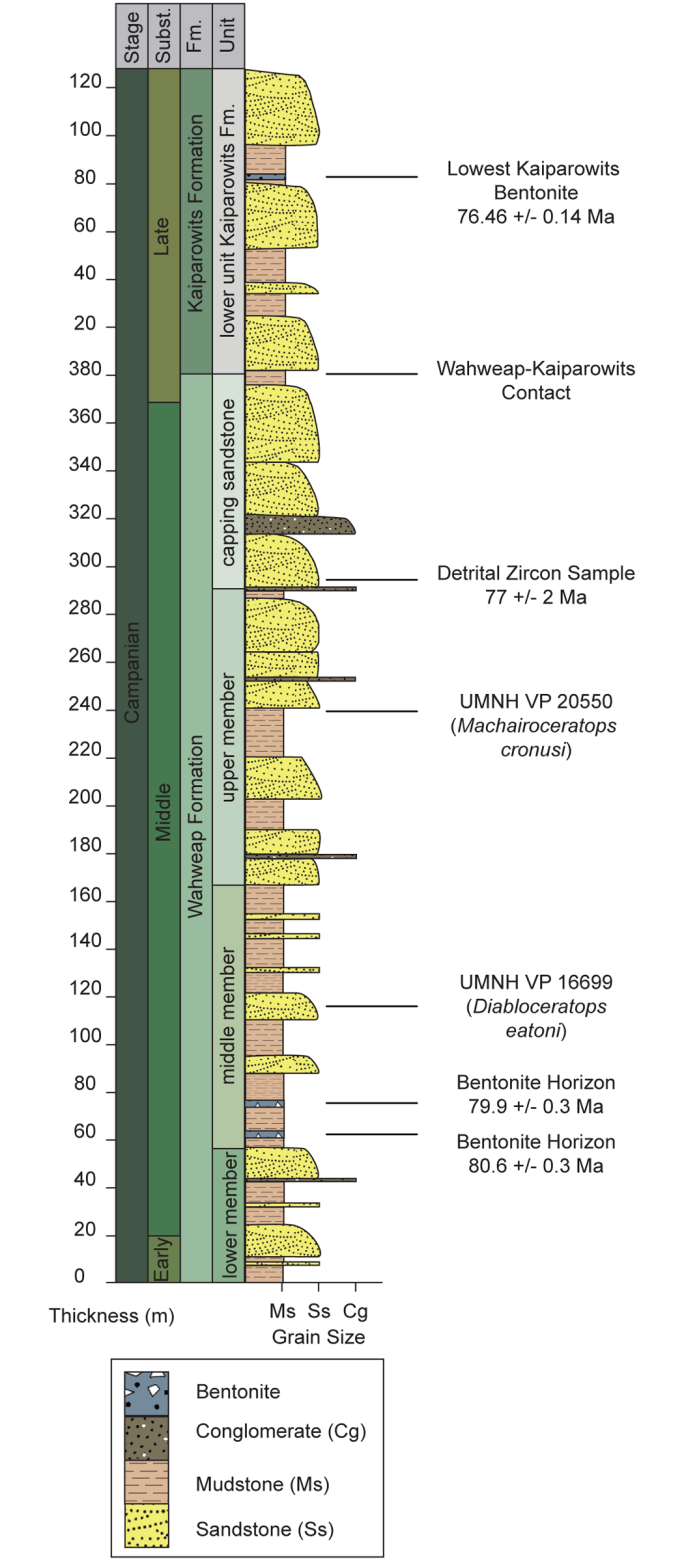
Schematic Stratigraphic Section of the Wahweap Formation. Schematic stratigraphic section of the Wahweap Formation within GSENM, southern Utah. Approximate stratigraphic positions of *Machairoceratops cronusi* (UMNH VP 20550) gen. et sp. nov. and *Diabloceratops eatoni* (UMNH VP 16699) indicated on the right of the column. Numbers to the right of the column represent dates obtained from radiometric dating of bentonite horizons and detrital zircons distributed discretely within the section (after [1–3]).

**Fig 3:**
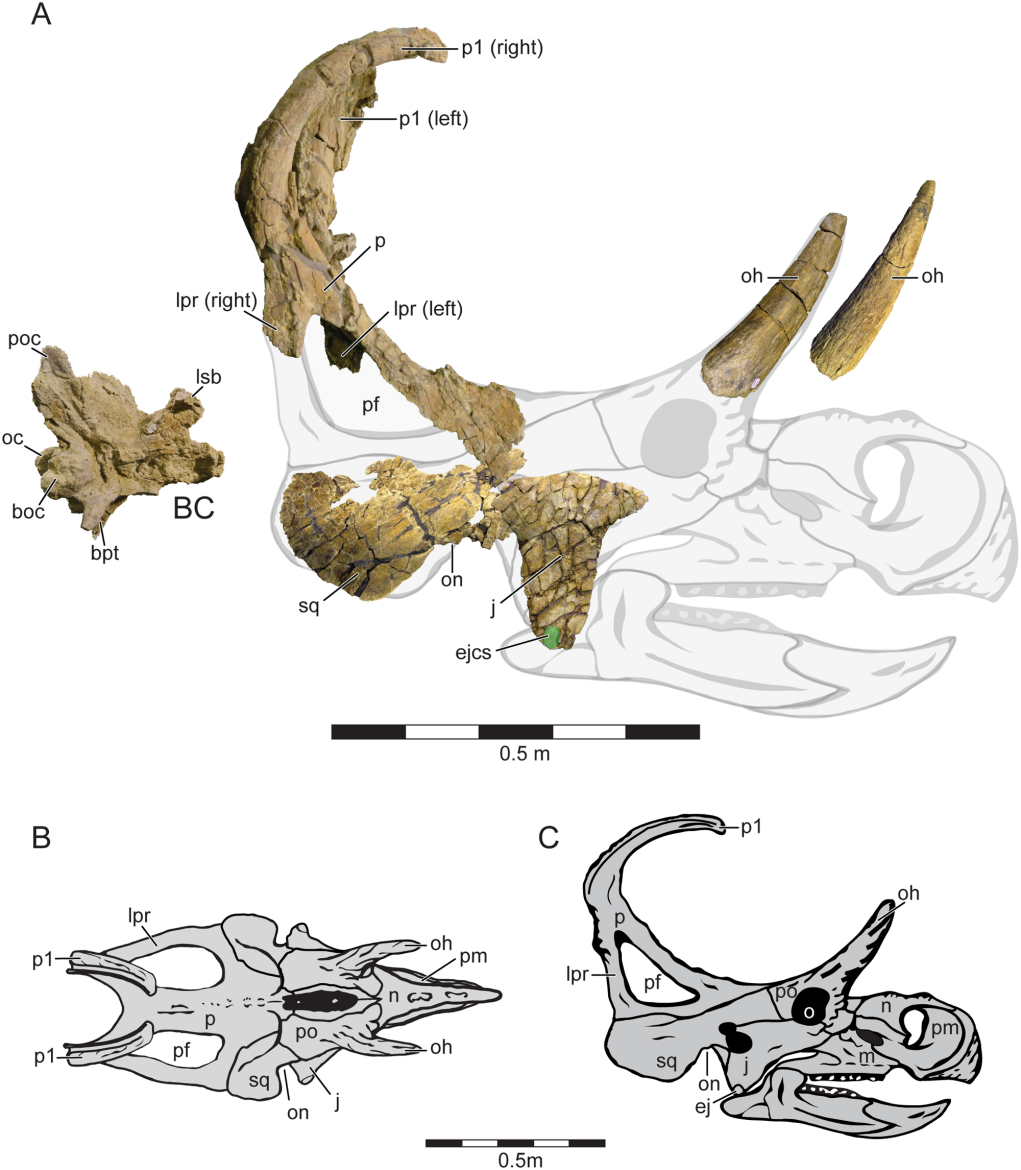
Holotype cranial Material and Cranial Reconstruction of *Machairoceratops cronusi* (UMNH VP 20550) gen. et sp. nov. Recovered cranial elements of *Machairoceratops* in right-lateral view, shown overlain on a ghosted cranial reconstruction (**A**). The jugal, squamosal and braincase are all photo-reversed for reconstruction purposes. *Machairoceratops* cranial reconstruction in dorsal (**B**), and right-lateral (**C**) views. Green circle overlain on the ventral apex of the jugal highlights the size of the epijugal contact scar (ejcs). **Abbreviations**: **BC**, braincase; **boc**, basioccipital; **bpt**, basipterygoid process; **ej**, epijugal; **ejcs**, epijugal contact scar; **j**, jugal; **lpr**, lateral parietal ramus; **lsb**, laterosphenoid buttress; **m**, maxilla; **n**, nasal; **o**, orbit, **oc**, occipital condyle; **oh**, orbital horn; **on**, otic notch; **p**, parietal; **pf**, parietal fenestra; **pm**, premaxilla; **po**, postorbital; **poc**, paroccipital process; **p1**, epiparietal locus p1; **sq**, squamosal. Scale bars = 0.5 m.

The Wahweap Formation is a ~ 400 m-thick succession of stacked fluviatile and estuarine clastic sediments delineated into four informal units: the lower, middle, and upper members, and the overlying capping sandstone, estimated to have been deposited between ~81 and 77 Ma (Fig 2) [2, 3]. The Wahweap Fm. contains one of the most diverse middle-late Campanian terrestrial faunas in North America and preserves multiple taxa of shark, rays, bony fish, crocodyliforms, turtles, lizards, mammals, and dinosaurs [4, 5]. Additionally, the Wahweap Fm. preserves the oldest diagnostic evidence of ceratopsids (all centrosaurines) in North America, with material known from each of the four members of the formation [6]. Only a single taxon, *Diabloceratops eatoni* (UMNH VP 16699) from the middle member, has thus far been formally named. Consequently, the phylogenetic affinities of other Wahweap Fm. ceratopsids remain ambiguous, largely due to the paucity of recovered diagnostic material [5, 6]. In 2006 new ceratopsid material (UMNH VP 20550) was recovered from a calcareous mudstone in the upper member of the Wahweap Formation (Figs 1 and 2). Over the course of two field seasons, a partial cranium that includes the braincase, portions of the lateral and dorsal dermal skull roof, and various facial and frill ornaments, all pertaining to a single individual, were recovered (Fig 3). No other faunal remains were recovered from the locality. The new material can be confidently placed within Centrosaurinae based on the subrectangular, fan-shaped, stepped-squamosal. The locality of the new specimen is stratigraphically higher in section than the locality from which *Diabloceratops eatoni* (UMNH VP 16699) was collected. Interestingly, the new specimen shares several morphologic features with *Diabloceratops*, including robust, elongate supraorbital ornamentation, a triangular (rather than round) parietosquamosal frill, and elongate spike-like epiparietal loci (p1) ornamentation. The epiparietal numbering scheme follows that proposed by Clayton et al., [7], where epiparietal loci are numbered according to their position along the posterior margin of the frill (e.g., p0 is located at the midline of the frill and p1 is positioned just lateral to p0, on either side of the midline). Any epiossifications or protuberances emanating from the dorsal surface of the frill near the midline are not given a number, but instead are recognized, simply, as a dorsal parietal process. The new ceratopsian material does, however, also exhibit unique morphologies that distinguish it from *Diabloceratops* and all other known centrosaurines, thereby increasing the known diagnostic centrosaurine fossil record from the southern portion of Laramidia.

## Materials and Methods

### Institutional Abbreviations

**AMNH**: American Museum of Natural History, New York, New York, USA; **ANSP**: The Academy of Natural Sciences, Philadelphia, Pennsylvania, USA; **CMN**: Canadian Museum of Nature, Ottawa, Ontario, Canada; **MOR**: Museum of the Rockies, Bozeman, Montana, USA; **MSM**: Mesa Southwest Museum, Mesa, Arizona, USA; **NHMUK**: The Natural History Museum, London, England, United Kingdom; **ROM**: Royal Ontario Museum, Toronto, Ontario, Canada; **TMP**: Royal Tyrrell Museum of Paleontology; Drumheller, Alberta, Canada; **UMNH VP**: Natural History Museum of Utah, Salt Lake City, Utah, USA; **WDC DJR**: Wyoming Dinosaur Center, Thermopolis, Wyoming, USA; **YPM**: Yale Peabody Museum of Natural History, New Haven, Connecticut, USA; **ZCDM**: Zhucheng Dinosaur Museum, Shandong Provence, China.

### Computed Tomography

The braincase of *Machairoceratops* (UMNH VP 20550) was scanned on a Philips Brilliance computed tomography (CT) 64-channel medical scanner using the following protocol: 120 kV, 377 mA, and a slice thickness of 1 mm with a 0.5 mm overlap between slices. Digital visualization of raw DICOM files was completed in Avizo 8.0 (Visualization Science Group (VSG)/FEI, U.S.A.).

### Phylogenetic Protocol

Hypotheses regarding the phylogenetic relationships of *Machairoceratops cronusi* within Ceratopsidae were evaluated using both standard parsimony and model-based (Bayesian) approaches. The character scorings for *Machairoceratops* were added to the data matrix of [8] using the character definitions of the same, but expanded to include the taxon *Wendiceratops pinhornensis* from [9]. The analyses utilized 26 taxa with respect to 101 characters (80 cranial and 21 postcranial). See supplementary materials: Appendix A in S1 File (specific taxon used for character scoring), Appendix B in S2 File (coded character definitions), and S1 Table (character-taxon matrix). As several characters clearly support the affiliation of *Machairoceratops* within Centrosaurinae, the selection of ingroup taxa included all valid centrosaurines and the two early-branching chasmosaurines *Chasmosaurus belli* and *Pentaceratops sternbergii*. In order to ensure proper character polarization and determine the phylogenetic affinity of *Machairoceratops* within Ceratopsia, we included three protoceratopsians (*Magnirostris*, *Bagaceratops*, and *Protoceratops*) and several early-branching nonceratopsid neoceratopsians (*Leptoceratops*, *Turanoceratops*, and *Zuniceratops*) in the analysis. *Leptoceratops* has been recovered as the proximate sister taxon to Coronosauria in recent analyses and as such was constrained as the outgroup taxon [10, 11].

The parsimony analysis was conducted in PAUP version 4.0b10 [12] employing the heuristic search option implemented under the parsimony criterion with random addition and tree bisection and reconnection (TBR) branch swapping, and cycled through 10,000 repetitions. All characters were assessed under an equal-weight model, with most treated as unordered. The one exception to the latter is that character 20 was run ordered based on ontogenetic data [13, 14]). Multistate characters were run as polymorphic and zero length branches were collapsed if they lacked support under the parsimony framework. Tree statistics including tree length, Consistency Index (CI) and Retention Index (RI) were calculated in PAUP. In order to assess the robusticity of the resultant topology, bootstrap proportions were calculated in TNT 1.1 (Trees using New Technology) using 10,000 bootstrap replicates, and employing 10 random addition sequence replicates per bootstrap replicate [15–17]. Additionally, Bremer support values were calculated implementing negative constraints as employed by the BREMER.RUN script supplied with TNT [15].

In addition to the parsimony-based analysis discussed above, a Bayesian phylogenetic analysis was conducted in order to evaluate the phylogenetic relationships of *Machairoceratops* within a model-based framework and to ameliorate ambiguities (e.g., low Bremer support values) that are produced in the parsimony analysis. Bayesian analytical techniques are becoming an increasingly common tool for morphology-only cladistic analyses with several recent studies implementing a Bayesian approach [18–27]. The Bayesian analysis discussed herein, generally follows the protocol of [26] in which an assumed ‘morphological clock’ model is used to simultaneously infer phylogenetic relationships and divergence dates using both morphological and taxon age range (‘tip-dating’) data in MrBayes 3.1.2 [27–29]. Stratigraphic age for each fossil taxon was constrained following [8], and is used here as either the mean age of a taxon based on the maximum and minimum stratigraphic occurrence or the most probable age range of a taxon based on stratigraphic placement. The data set mirrors that used for the parsimony-based analysis discussed above consisting of 101 characters arrayed across 21 ceratopsid in-group taxa and 6 out-group taxa. For simplicity of analysis, autapomorphies were excluded from the study despite their potential to impact the analysis results (e.g., branch lengths; [18, 19, 30]). The tree was rooted on the branch between *Leptoceratops* and Centrosaurinae, as Centrosaurinae has long been established as a monophyletic clade [31–40]. All characters were run equally weighted and unordered (excluding character 20) as in the parsimony analysis above.

The Bayesian analysis utilized an MK likelihood model [30] implemented with a variable rates parameter (assuming a gamma-distribution) of character state changes, and an uncorrelated relaxed clock parameter assuming variable rates of change across branches. Both of these model parameters were preferable to an equal rates model that assumes equal rates of character change, and a strict clock model where evolutionary rate is held constant throughout the tree [26]. The default priors in MrBayes 3.1.2 were used throughout the analysis, unless otherwise specified (e.g., strict clock model). The analysis used four replicate runs of 20,000,000 iterations, sampling every 1,000 generations with 4 chains (1 ‘cold’ chain and 3 incrementally ‘hot’ chains sampling the tree space). The initial 25% of sampled generations were discarded as the ‘burn-in’ phase before the analysis converges on stationarity, with the remaining samples used to calculate the summary statistics (e.g., consensus tree) [28, 29]. Within the analysis, all replicate runs converged on nearly identical tree topologies (average standard deviation of clade frequencies across replicates = 0.008) and parameters (Potential Scale Reduction Factor (PSRF) at or close to 1.0) [41]. A majority-rule consensus tree was created through combination of all post burn-in samples for all four replicate runs. Exact parameter settings in MrBayes commands are shown in a supplemental appendix (Appendix C in S3 File).

### Paleontological Ethics Statements

The holotype specimen described herein (UMNH VP 20550) is permanently reposited in the collections of the Natural History Museum of Utah, 301 Wakara Way, Salt Lake City, Utah, USA. Detailed locality information is available from the museum registrar as per museum policy. All pertinent permits were obtained for the described study, which conformed to all relevant regulations. UMNH VP 20550 was collected under permits (permit Nos. UT-S-05-028, UT08-00NE-GS) received from the United States Department of the Interior’s Bureau of Land Management (BLM) for work conducted in the BLM-regulated Grand Staircase-Escalante National Monument.

### Nomenclatural Acts

The electronic edition of this article conforms to the requirements of the amended International Code of Zoological Nomenclature (ICZN), and hence the new names contained herein are available under that Code from the electronic edition of this article. This published work and the nomenclatural acts it contains have been registered in ZooBank, the online registration system for the ICZN. The ZooBank LSIDs (Life Science Identifiers) can be resolved and the associated information viewed through any standard web browser by appending the LSID to the prefix “http://zoobank.org/”. The LSID for this publication is: urn:lsid: zoobank.org:pub: E19AA0BC-82E4-481A-BB69-95AA3665367E. The electronic edition of this work was published in a journal with an ISSN, and has been archived and is available from the following digital repositories: PubMed Central, LOCKSS, and Morphobank.

## Results

### Systematic Paleontology

Systematic hierarchy.

Ornithischia Seeley, 1887 [42] *sensu* Sereno 1998 [43]

Ceratopsia Marsh, 1890 [44] *sensu* Dodson 1997 [45]

Ceratopsidae Marsh, 1888 [46] sensu Sereno 1998 [43]

Centrosaurinae Lambe, 1915 [31] sensu Dodson et al., 2004 [37]

*Machairoceratops* gen. nov.

urn:lsid:zoobank.org:act: F8351E74-0476-425F-AC6A-04C57CFC8AA1

*Machairoceratops cronusi*, gen. et. sp. nov.

urn:lsid:zoobank.org:act: F1863F1B-4151-4B06-A1E4-9808A2CF2A9

#### Etymology

*Machairoceratops*, from *machairis* (Greek), bent sword, in reference to the posterodorsally projecting, anteriorly curved epiparietal (locus p1) ornamentation, and *ceratops* (Latinized Greek), horned-face. The specific epithet *cronusi* refers to the Greek god Cronus who, according to mythology, deposed his father Uranus with a sickle or scythe, and as such is depicted carrying a curved bladed weapon.

#### Holotype

The holotype specimen is UMNH VP 20550, an associated partial skull including two curved and elongate orbital horncores, left jugal, nearly complete, slightly deformed braincase, left squamosal, and a parietal complex ornamented by caudally projecting, rostrally curved, elongate spikes on either side of a midline embayment. All material is reposited at the Natural History Museum of Utah, Salt Lake City, Utah, United States of America.

#### Type, locality, horizon and age

Grand Staircase-Escalante National Monument (GSENM), Kane County, southern Utah, U.S.A. Stratigraphically, *Machairoceratops* occurs within the upper member (~200–350 m) of the late Campanian Wahweap Formation, which is currently dated between ~80.1–77 Ma (Fig 2) [2, 3].

#### Diagnosis

Centrosaurine ceratopsid diagnosed by the following autapomorphies: posteriorly projecting, anteriorly curved spike-like epiparietal loci (p1) ornamentation, that also exhibits a posteromedially directed sulcus along the entire length of the epiparietal differing from all other sulci present on ceratopsian epiossifications in width, depth, and overall conformation. *Machairoceratops* differs from the stratigraphically lower *Diabloceratops* in a number of key features including: a fan-shaped, subrectangular (rather than rectangular) stepped squamosal, an inferred (based on size and shape of the epijugal contact facet) smaller, elliptical (rather than tetrahedral) epijugal, two anterodorsally (rather than laterally) curved (p1) epiparietals on either side of a midline embayment, and a posteromedially oriented sulcus running the entire length of the posterior surface of the epiparietal loci (p1) ornamentation. Additionally, *Machairoceratops* differs from several roughly contemporaneous centrosaurines from the northern portion of Laramidia (e.g., *Albertaceratops nesmoi*, *Coronosaurus brinkmani*, and *Spinops sternbergorum*) in possessing a triangular (rather than rounded) parietosquamosal frill, and in the morphology and orientation of the epiparietal ornamentation as described above.

## Description and Comparisons

### Circumorbital Regions

#### Supraorbital ornamentation

*Machairoceratops* preserves both right and left supraorbital horncores (~270 mm in length for both). Both are broken and isolated from the rest of the skull (Fig 3A). It is assumed that as in all ceratopsids, the supraorbital ornamentation occurred as outgrowths of the postorbital, the rest of which is not preserved. In addition to being elongate and robust, the supraorbital horncores are morphologically similar to other early-branching ceratopsids (e.g., *Albertaceratops*, TMP 2001.26.1; *Diabloceratops*, UMNH VP 16699; *Nasutoceratops*, UMNH VP 16800) in being elongate, subcircular in cross-section, tapering distally to a point, and possessing numerous longitudinal ridges and groves on the external surface. The true horncore orientation and position relative to the orbit cannot be confidently determined due to the incomplete nature of the proximal end of each element.

#### Jugal

*Machairoceratops* preserves a nearly complete, but erosionally damaged left jugal (Fig 3A). The jugal (UMNH VP 20550) is missing much of the dorsal margin, including portions that contribute to the ventral margin of the orbit in addition to the contact facets for the lacrimal (anterodorsally), the maxilla (anteriorly), the postorbital (posterodorsally), and the squamosal (posteriorly). Overall the jugal exhibits morphology typical of other centrosaurines (e.g., *Centrosaurus*, ROM 767; *Albertaceratops*, TMP 2001.26.1) in being triangular with one apex positioned ventrally. The jugal measures 235 mm from the ventral apex to the dorsal-most margin. The contact facet for an epijugal ossification is typical of other centrosaurines in being relatively small in area (Fig 3A green overlay) with the longest diameter axis (~ 30 mm) being dorsoventrally oriented. This conformation suggests a smaller, more elliptical (rather than tetraheadral) epiossification for *Machairoceratops* as compared to the relatively large, tetrahedral morphology observed in *Diabloceratops*.

### Parietosquamosal Frill

#### Squamosal

**Fig 4:**
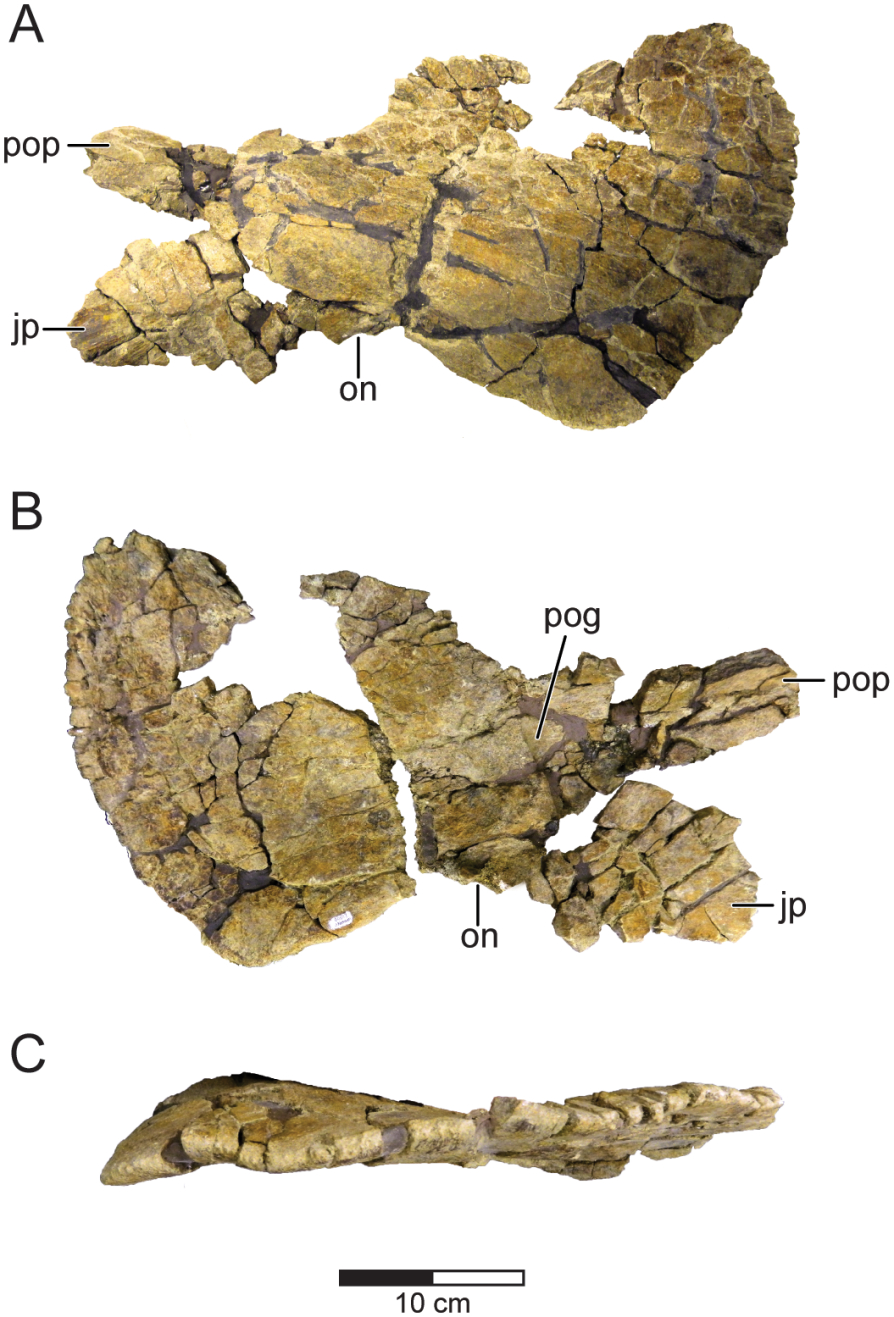
Left squamosal of *Machairoceratops cronusi* (UMNH VP 20550) gen. et sp. nov. *Machairoceratops* squamosal in lateral (**A**), medial (**B**), and caudal (**C**) views. **Abbreviations**: **jp**, jugal process; **on**, otic notch; **pog**, paroccipital groove; **pop**, postorbital process. Scale bar = 10 cm.

The left squamosal of *Machairoceratops* (UMNH VP 20550) was recovered from the quarry, although the dorsal margin of the element is variably preserved (Figs 3A and 4). The shape of the element is mostly intact and characteristically centrosaurine, being squared off anteromedially, and having the diagnostic ‘stepped-up’ dorsal margin (Figs 3A and 4). The squamosal measures 240 mm from the distal margin of the parietosquamosal contact to the approximate anteroventral corner of the free blade just posterior to the otic notch. Unfortunately, the surrounding contacts with the jugal (anteroventrally), postorbital (anteriorly), parietal (dorsally), and quadrate (ventrally) are not preserved. The dorsal surface of the squamosal is marginally preserved being slightly root damaged and fractured; however, a weakly-developed ridge extending from the anteromedial margin to the anteroventral corner of the free blade is observable, a morphology that is typically noted for all ceratopsids [9]. The squamosal differs from that of *Diabloceratops* in being fan-shaped and subrectangular (rather than rectangular), possessing a relatively constricted otic notch, and with a relatively large parietosquamosal contact step. The squamosal lacks any fused epiossifications (i.e., episquamosals) along the posterior margin but there are undulations suggesting the presence of 4 episquamosal loci.

#### Parietal

**Fig 5:**
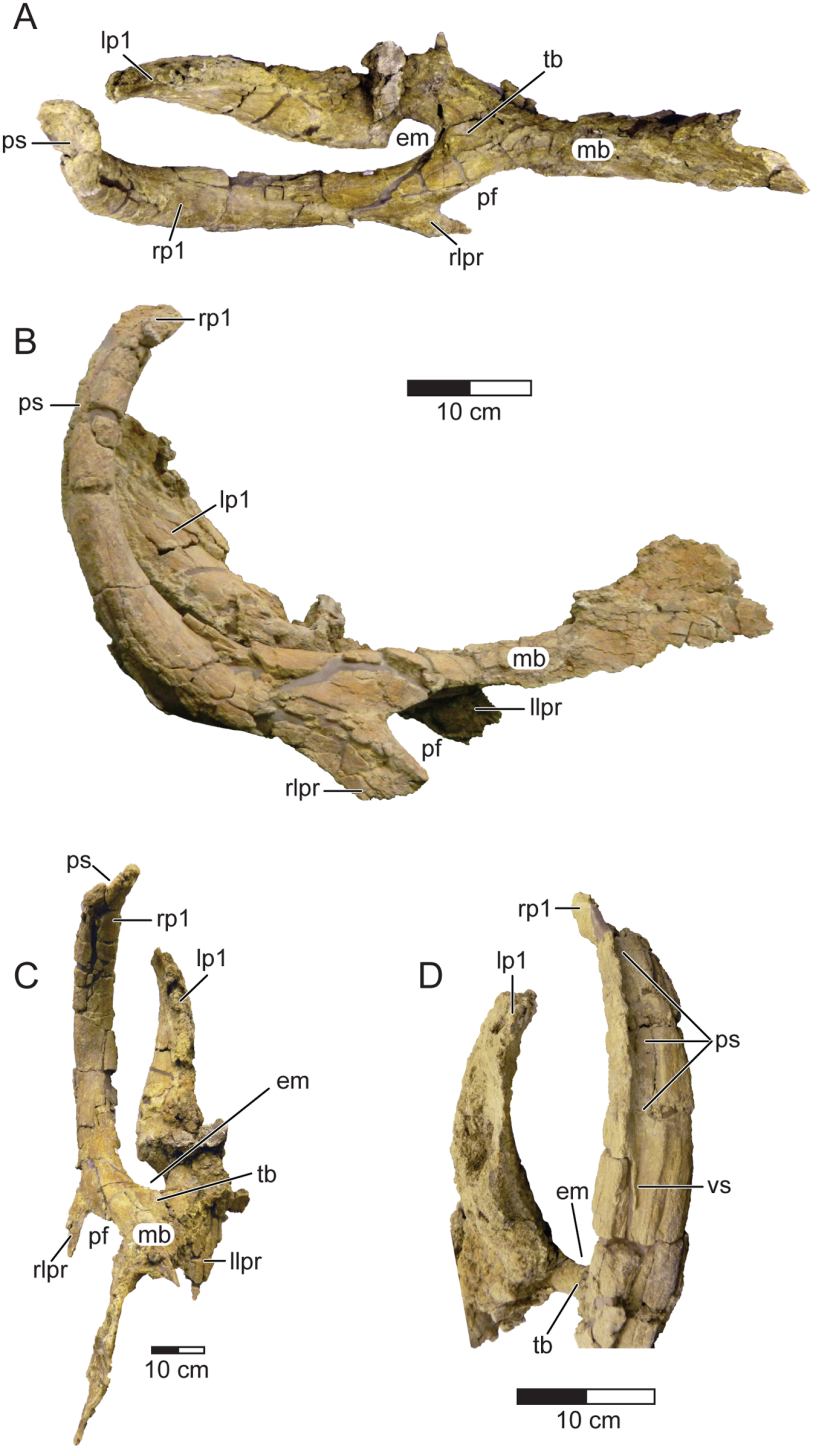
Parietal of *Machairoceratops cronusi* (UMNH VP 20550) gen. et sp. nov. *Machairoceratops* parietal complex in dorsal (**A**), right-lateral (**B**), rostral (**C**), and close-up caudal (**D**) views. **D** highlights the autapomorphic posteromedially-oriented sulcus. **Abbreviations**: **em**, midline embayment; **lpr**, lateral parietal ramus (r = right, l = left); **mb**, median bar; **pf**, parietal fenestra; **p1**, epiparietal locus p1 (r = right, l = left); **ps**, posteromedial sulcus, **tb**, transverse bar, **vs**, vascular sulcus. Scale bars = 10 cm.

The parietal of *Machairoceratops* (UMNH VP 20550) is nearly complete, preserving most of the median bar, the entire transverse bar complete with caudally projecting, rostrally curved epiparietal loci (p1) ornamentations on either side of a midline embayment, but missing most of both lateral rami (Figs 3 and 5). The epiparietal numbering scheme follows that proposed by [7], which is a deviation from the ‘traditional’ homologies for epiparietal ossifications [37]. The parietal lacks any other epiparietal ornamentations, and this conformation could be due to one or more of the following: 1) *Machairoceratops* truly lacks any other epiparietal ossifications, making the frill distinct from all other centrosaurines; 2) the lack of any other fused epiparietal ornamentation suggests *Machairoceratops* is a juvenile or young subadult individual; and/or 3) the lack of any other epiparietal ornamentation is due to taphonomic processes which have removed the ossifications. The parietal is similar in general morphology to that observed in *Diabloceratops* (UMNH VP 16699), offering a triangular (rather than round) “M-shaped” conformation to the frill (in dorsal view), and possessing two spike-like p1 epiparietals. The right p1 epiparietal is nearly complete (~440 mm in length) and spike-like, with a flattened tongue-like apex (rather than a point), and preserving a posteromedially oriented sulcus along the entire length of the posterior surface (Fig 5D). This posteromedial sulcus differs in morphology to all other sulci observed on ceratopsian epiossifications in width, depth, and overall morphology. In addition, the bone surface forming the lateral walls and floor of the sulcus, where adequately preserved, is marked by vascular sulci similar to those seen on all other ceratopsian epiossifications, supporting this character as an autapomorphy of *Machairoceratops* (Fig 5D). The left p1 epiparietal, however, is badly crushed and erosionally damaged, making its original conformation difficult to characterize. However, it is assumed to be similar in form to the right epiparietal (p1) spike. Despite the erosional and modern root damage to the surface of the parietal, the taphonomic deformation affecting the element appears to be nominal. The relatively uncrushed preservation of the parietal as a whole, and the consistency in morphology, along the entire length, of the right p1 epiparietal coupled with the similar orientation of both p1 epiparietals, indicates the morphology and orientation is not due to taphonomic distortion.

In contrast to the epiparietal morphology present in *Diabloceratops*, the epiparietals in *Machairoceratops* are anterodorsally (rather than laterally) directed and possess a posteromedially oriented sulcus along the entire length of the posterior surface. Additionally, the epiparietal spikes in *Machairoceratops* differ in being more robust overall and have a comma-shaped (rather than semicircular) cross-section along most of the length of the process (excluding the base). Furthermore, the epiparietals differ in the shape of their terminal apex, with *Machairoceratops’* terminating in a flattened tongue-shaped end where those of *Diabloceratops* terminate in a point.

Unlike other centrosaurines possessing elongate epiparietal spikes (e.g., *Spinops sternbergorum* [NHMUK R16307], *Einiosaurus procurvicornis* [MOR 456], and *Styracosaurus albertensis* [CMN 344]), the epiparietal spikes of *Machairoceratops* differ in being strongly anterodorsally curved, being marked by a prominent posteromedially oriented sulcus along the entire length of the process, having a comma-shaped cross-section (rather than flat, round, or semicircular), and terminate in a flattened tongue-like apex (rather than a pointed apex). Farke et al. [47] noted variably shaped longitudinal sulci marking the dorsal and medial surfaces of the elongate epiparietal (p2) spikes in *Spinops sternbergorum* (NHMUK R 16307); however, these sulci, and the epiossifications as a whole, can be distinguished from the morphology seen in *Machairoceratops* by the unique suit of characters already outlined above.

The median bar is dorsoventrally restricted (~ 4 mm) near the margins (Fig 5B), thickening near the midline (~15 mm), with an overall wide, strap-like conformation similar to that in *Achelousaurus* (e.g., MOR 485). Anteriorly, the median bar is dorsally convex, forming a low, rounded median ridge, a synapomorphy for the Centrosaurinae [48]. The bar widens caudally near the apex of the parietal fenestrae to transition into the transverse parietal bar. The transverse bar is dorsoventrally rod-like and forms a “Y” with the midline embayment and epiparietal loci (p1) ornamentations that diverge posterodorsally.

### Braincase

**Fig 6:**
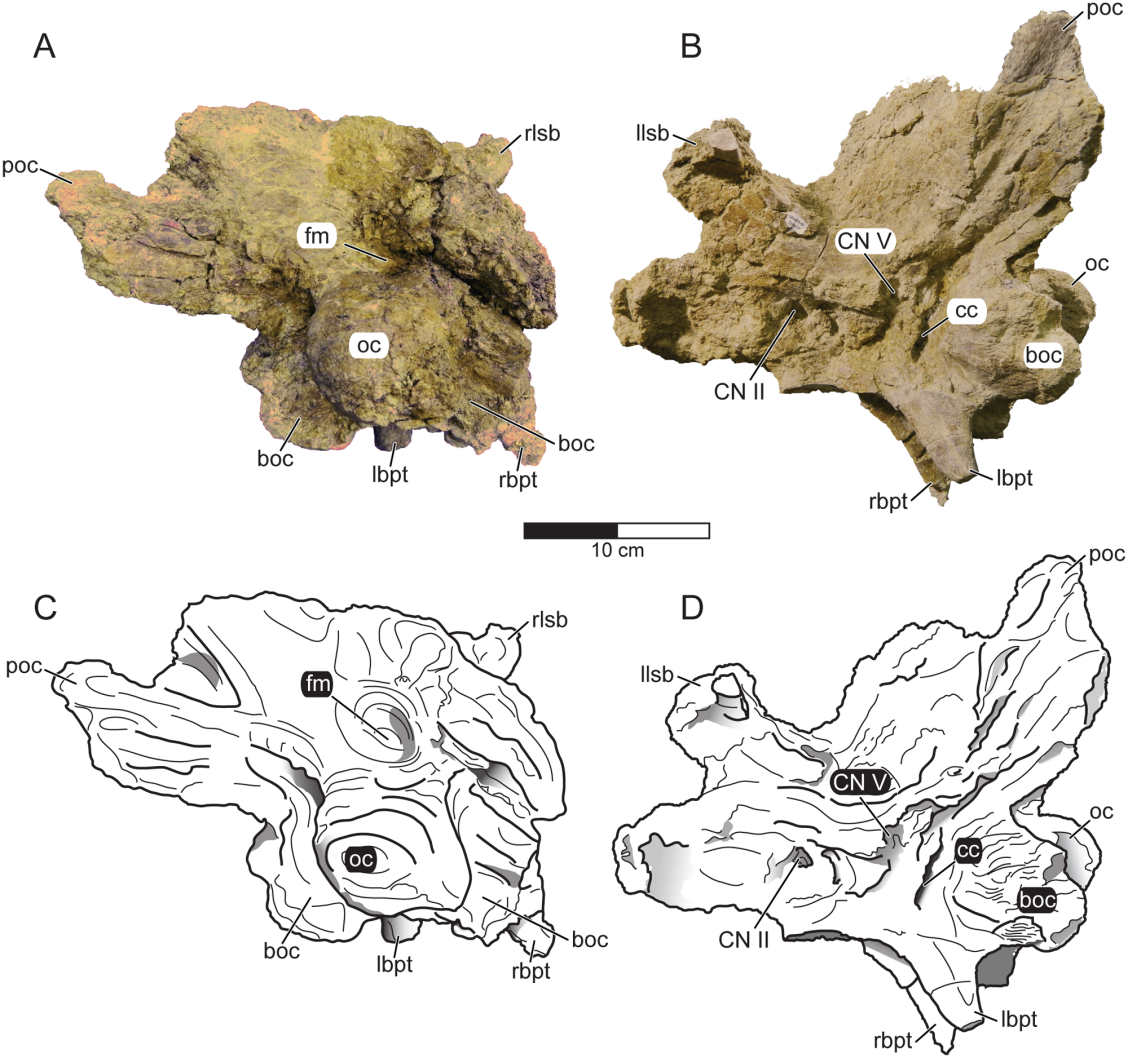
Braincase *Machairoceratops cronusi* (UMNH VP 20550) gen. et sp. nov. *Machairoceratops* braincase in occipital (**A**) and left-lateral (**B**) views, with corresponding line drawings in occipital (**C**) and left-lateral (**D**) views. **Abbreviations**: **boc**, basioccipital; **bpt**, basipterygoid process (r = right, l = left); **cc**, carotid canal; **CN II**, optic canal; **CN V**, trigeminal foramen; **fm**, foramen magnum; **lsb**, laterosphenoid buttress (r = right, l = left); **oc**, occipital condyle; **poc**, paroccipital process. Scale bar = 10 cm.

The braincase of *Machairoceratops* is disarticulated from the other cranial elements, but mostly complete, only missing portions of the right paroccipital process and supraoccipital (Fig 6; S4 File). Much of the surface and overall structure of the braincase has been damaged by modern roots, thereby obscuring much of the external braincase morphology (e.g., bone surface texture and sutural contacts). Additionally, the modern root damage has similarly affected the internal braincase morphology (i.e., CT scan data of the element), rendering all but the surface model relatively indeterminate (S5 File). Moreover, the braincase has undergone lateral shearing deformation causing the dorsal portion of the braincase to shear left relative to the ventral portion (Fig 6A and 6C). The foramen magnum has been slightly obscured (e.g., infilled with matrix) by fossilization, but is generally oval to subrectangular in conformation and measuring, as preserved, ~39.97 mm dorsoventral diameter and ~24.75 mm along the transverse diameter. In contrast to the morphology exhibited by several other ceratopsids (e.g., *Diabloceratops* UMNH VP 16699; *Pachyrhinosaurus* TMP 1989.55.1243), the braincase of *Machairoceratops* does not possess a pair of deeply excavatedfossae or a robust posteromedial ridge formed from the supraoccipital just dorsal to the foramen magnum (Fig 6). Instead, these features are only weakly developed. The weakly developed suproccipital ridge is, however, excluded from a contribution to the foramen magnum by the exoccipitals. The occipital condyle is characteristically ceratopsid, being ‘trailer-ball-hitch’ in conformation (i.e., subrounded to elliptical) and fully fused on a short neck that is ventrally deflected. The occipital condyle, as preserved, measures 46.15 mm in dorsoventral diameter and 55.36 mm along the transverse diameter. Directly below the occipital condyle the basioccipital supports two basal tubers/tuberosities, with only the left side being completely preserved. Immediately anterior to the basal tuberosities are the basipterygoid processes that are noticeably deflected to the right. The left lateral wall of the braincase is better preserved than the right, making it possible to identify several of the cranial nerve openings (e.g., CN II, CN V; Fig 6C and 6D). The size and position of the cranial nerve openings, as preserved, in the braincase of *Machairoceratops* are consistent with the size and position of those described for *Pachyrhinosaurus lakustai* (TMP 1989.55.1243), but differ slightly with regard to position for those described in *Diabloceratops* [5, 40]. Specifically, the location of CN II in *Diabloceratops* is described by Kirkland and DeBlieux [5] as being located dorsal to CN III and slightly anterodorsal to CN V. This is in contrast to the morphology noted for *Machairoceratops* in which the opening of CN II is anterior to CN V. Unfortunately, modern root damage and the preservation of the braincase makes further comparisons difficult.

The braincase of ceratopsid dinosaurs are distinctive in their morphology when compared to other dinosaurs, but tend to be conservative throughout the clade and vary predominantly in size [40]. The braincase of *Machairoceratops* appears generally similar in conformation to other centrosaurines (e.g., *Diabloceratops* UMNH VP 16699; *Centrosaurus* ROM 767; *Pachyrhinosaurus* TMP 1989.55.1243); however, detailed anatomical comparisons between specimens is difficult due to the paucity of well-preserved disarticulated braincases, not to mention the fact that individual braincase elements are typically obscured by fusion with adjacent elements.

### Ontogenetic status of UMNH VP 20550

The holotype of *Machairoceratops cronusi* is very nearly, if not the same size as the postulated adult holotype of *Diabloceratops eatoni* (UMNH VP 16699). However, several characteristics suggest *Machairoceratops* represents a juvenile to subadult individual. Namely, the lack of fused epiparietal ornamentation, and the disarticulation of the braincase. Interestingly, *Machairoceratops* also exhibits exclusion of the supraoccipital from the foramen magnum, a trait typically associated with skeletally-mature individuals in ceratopsians [14]. Unfortunately the surface texture of UMNH VP 20550 is not preserved in enough detail due to modern root damage to bring this line of evidence to the discussion. Thus, the ontogenetic status of *Machairoceratops* remains ambiguous until more definitive materials are recovered.

### Phylogenetic Analysis

**Fig 7:**
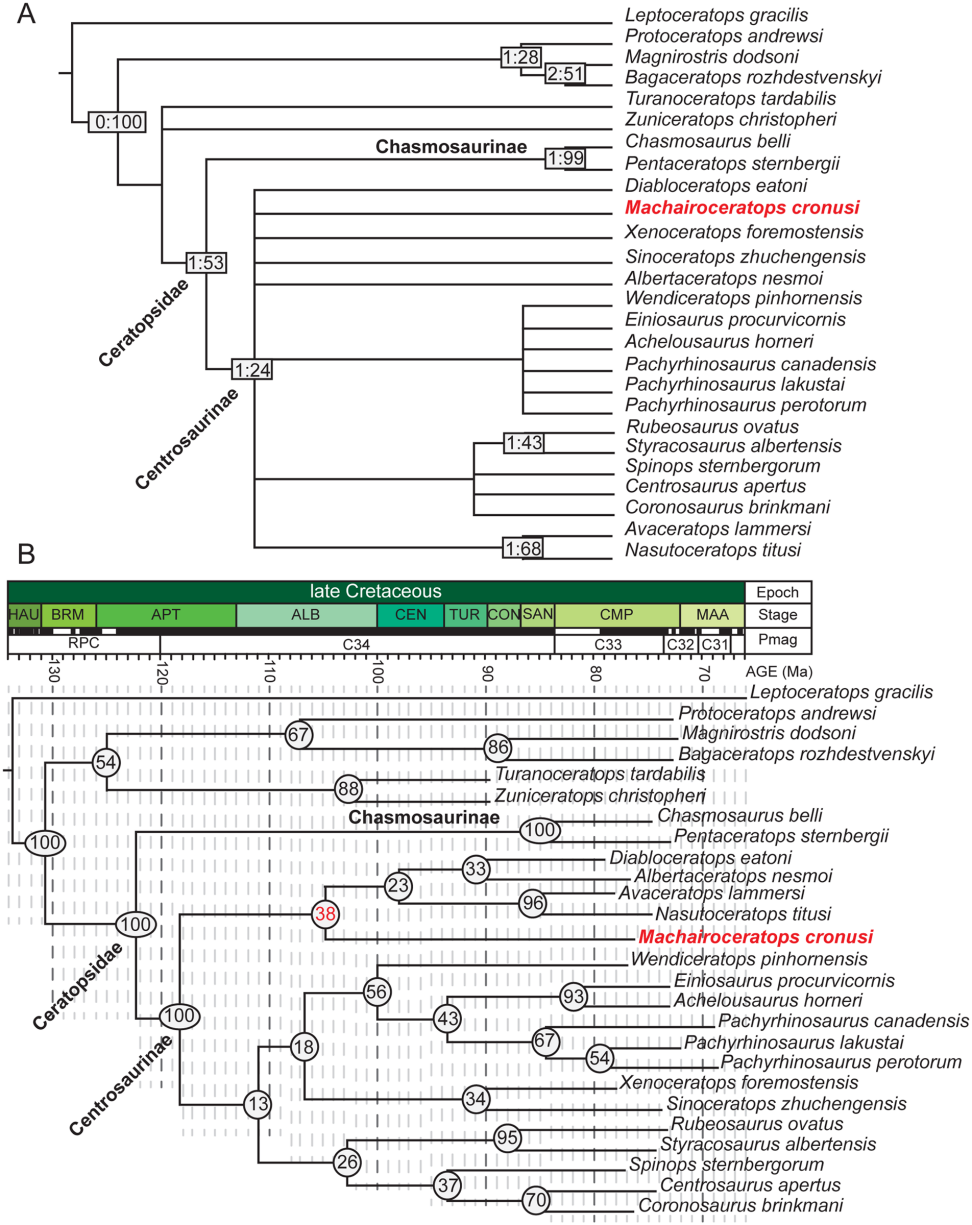
Evolutionary Relationships of *Machairoceratops cronusi* (UMNH VP 20550) gen. et sp. nov. Results of the maximum parsimony and Bayesian phylogenetic analyses. Strict consensus of 1194 most parsimonious trees (tree length = 160, CI = 0.675, RI, 0.818, RCI = 0.552) of an analysis of 101 characters arrayed across 26 ceratopsian taxa (**A**). Numbers in node boxes indicate Bremer support indices to the left and bootstrap proportions presented as Groups Present/Contradicted (GC) values to the right. Resultant time-calibrated Bayesian topology (i.e., majority rule consensus tree of all sampled trees) from the morphological clock model with posterior probabilities at each node (**B**). **Abbreviations**: **ALB**, Albian; **APT**, Aptian, **BRM**, Barremian; **CEN**, Cenomanian; **CMP**, Campanian; **CON**, Coniacian; **HAU**, Hauterivian; **MAA**, Maastrichtian; **Pmag**, Paleomagnetism; **RPC**, Rapid Polarity Changes.

A phylogenetic analysis employing maximum parsimony recovered 1194 MPTs with tree lengths of 160 steps, consistency indices (CI) of 0.675, rescaled consistency indices (RCI) of 0.552, and retention indices (RI) of 0.818. The strict consensus tree is reported herein (Fig 7A). The resolution of the parsimony analysis was relatively poor, being unable to resolve the position of *Machairoceratops* within Ceratopsidae and placing the taxon in a large unresolved polytomy with the centrosaurines *Diabloceratops eatoni* (UMNH VP 16699), *Albertaceratops nesmoi* (TMP 2002.26.1), *Sinoceratops zhuchengensis* (ZCDM V0010), and *Xenoceratops foremostensis* (CMN 53282)and, a grouping consisting of all other centrosaurines (i.e., [(*Nasutoceratops* + *Avaceratops*)], [[[(*Rubeosaurus* + *Styracosaurus*)] + [(*Spinops* + *Centrosaurus* + *Coronosaurus*)]], and [(*Einiosaurus* + *Wendiceratops* + *Achelousaurus* + *P*. *canadensis* + *P*. *lakustai* + *P*. *perotorum*)]). The robusticity of the analysis is comparatively weak for most clades, with bootstrap proportions and Bremer support values being well below 50% and only 1, respectively (Fig 7A). However, *Machairoceratops* can still be confidently placed within Centrosaurinae on the basis of the squamosal that is anteroposteriorly abbreviated with a sub-rectangular outline (Character 41 [0]) and possessing a ‘stepped-up’ dorsal margin, and the relatively wide, strap-like midline parietal bar (Character 52 [1]). Notably, the parsimony analysis presented herein differs from that of two recent analyses of Centrosaurinae (i.e., [9, 49]) in the position of several early-branching taxa. In addition to the relatively poor resolution of the analysis, the topology presented herein (Fig 7A) differs in the positions of *Xenoceratops foremostensis* (CMN 53282) and *Wendiceratops pinhornensis* (TMP 2011.051.0009), both from the middle Campanian of Alberta, Canada. The recent analysis of *Xenoceratops* [49] places this taxon as the most earliest-branching centrosaurine. By contrast, the analysis results reported herein positions *Xenoceratops* in a large polytomy with *Diabloceratops*, *Machairoceratops*, *Albertaceratops*, and *Sinoceratops*. Another recent analysis [9] introduced *Wendiceratops* as the sister taxon to *Sinoceratops zhuchengensis*, whereas the results reported herein position *Wendiceratops* in a large unresolved polytomy nested within Centrosaurinae. Lability of *Machairoceratops* (this study), *Wendiceratops* [9], *Xenoceratops* [49], and *Sinoceratops* [50] primarily results from a lack of overlapping elements with other centrosaurines, particularly early-branching members of the clade (e.g., *Diabloceratops* [UMNH VP 16699], *Albertaceratops* [TMP 2002.26.1]), and missing or poorly preserved materials distinguishing each taxon.

The Bayesian analysis (Fig 7B) is generally congruent with the parsimony analysis described above, for both the strict consensus (Fig 7A) and 50% majority rule (S1 Fig) consensus trees. Specifically, *Machairoceratops* is placed as an early-branching ceratopsid. However, in contrast to the parsimony analysis, the Bayesian results reveal increased resolution at the base of Centrosaurinae, aligning *Machairoceratops* as the sister taxon to two clades, one consisting of *Diabloceratops* (UMNH VP 16699) from the lower Wahweap Formation and *Albertaceratops* (TMP 2002.26.1) from the Oldman Formation of Alberta, Canada and a second clade consisting of *Nasutoceratops* (UMNH VP 16800) from the overlying Kaiparowits Formation and *Avaceratops* (ANSP 15800) from the Judith River Formation of Montana. Note that the posterior probability (PP) for this sister-taxon relationship is only marginally supported at 38%. Other notable differences between the parsimony and Bayesian approaches include the position of several taxa including *Xenoceratops* [CMN 53282], *Albertaceratops* [TMP 2002.26.1], *Sinoceratops* [ZCDM V0010], *Einiosaurus* [MOR 373], *Achelousaurus* [MOR 485], and *Wendiceratops* [TMP 2011.051.0009]. *Xenoceratops* and *Sinoceratops* become allied as sister taxa, as do *Albertaceratops* and *Diabloceratops* with these unions being marginally supported with PP’s of 34% and 33%, respectively. The placement of *Einiosaurus* also differs between the two analyses in that *Einiosaurus* is allied with *Achelousaurus*, with this union being generally well supported with a PP of 93%. It should be noted however, that except for the union of *Einiosaurus* and *Achelousaurus*, support for the new positions of the aforementioned taxa is low being well below 70% (Fig 7B). These ambiguities are most likely due to the lack of overlapping material among these taxa as well as missing or poorly preserved material diagnosable to these taxa.

## Discussion

Historically, the ceratopsid fossil record preceding the group’s late Campanian radiation (approximately 77 MA) in North America has remained relatively enigmatic despite a handful of taxa (e.g., *Avaceratops lamersi* [ANSP 15800]; *Albertaceratops nesmoi* [TMP 2002.26.1]; *Coronosaurus brinkmani* [TMP 2002.68.1–3]; *Diabloceratops eatoni* [UMNH VP 16699]; *Xenoceratops foremostensis* [CMN 53282]; *Medusaceratops lokii* [WDC DJR 001]; *Judiceratops tigris* [YPM 022404]) being described from this formative period in the clade’s evolutionary history [9]. The majority of these taxa (e.g., *Xenoceratops*, *Judiceratops*, *Medusaceratops*) however, are only known from fragmentary material and no doubt contribute to our poor understanding of the early evolutionary history of the clade [49, 51, 52]. The recovery of *Machairoceratops cronusi* from the upper member of the Wahweap Formation of GSENM, southern Utah helps provide important insights into this early radiation of late Campanian ceratopsids from southern Laramidia. Currently the oldest recognized member of Ceratopsidae is the centrosaurine *Diabloceratops eatoni* (UMNH VP 16699), a form known from the ~80 Ma Wahweap Formation in the southern portion (southern Utah) of Laramidia [5]. *Diabloceratops* is known from a single individual and suggests that diminutive nasal ornamentation along with large supraorbital ornamentation and relatively unadorned, triangular frills represent plesiomorphic traits for the clade. *Machairoceratops*, also from the Wahweap Formation, is dated to between 77 to 80 ± 2 Ma, and reinforces the interpretation of these characters by exhibiting large orbital horns and a relatively unadorned, triangular parietosquamosal frill (Fig 3). The retention of such characters in *Machairoceratops* for approximately two million years provides insights into the selective evolutionary pressures and the evolutionary tempo acting upon the ceratopsid taxa from the Wahweap Formation during the middle to late Campanian. For example, retention of a relatively unadorned, triangular (rather than round) parietosquamosal frill suggest the presence of natural or sexual stabilizing selection acting upon this character trait. And similarly, retention of such traits further suggests relatively stable or slow evolutionary tempos acting upon the ceratopsians from the Wahweap Formation. The retention of these traits highlights potential differences regarding the evolutionary constraints (natural or sexual) acting upon northern Laramidian centrosaurines versus southern Laramidian centrosaurines, further supporting the idea of dinosaur provincialism within Laramidia during the late Cretaceous. Additionally, *Machairoceratops* expands the diversity of parietosquamosal frill ornamentation by the possession of the autapomorphic posteromedial sulcus running the entire length of the epiparietal (p1) ornamentation (Figs 3 and 5D). The overall conformation is unique with respect to Centrosaurinae, suggesting evolutionary experimentation in parietal ornamentation by centrosaurines of this time. Finally, the discovery of *Machairoceratops* provides evolutionary and biogeographic support for the hypothesis of a southern Laramidian origination and subsequent northern dispersal of centrosaurine ceratopsids throughout Laramidia by approximately 79 Ma when considered together with the oldest recognized member of Ceratopsidae from northern Laramidia (i.e., *Xenoceratops Foremostensis* [CMN 53282]), a form dated to approximately 79 Ma; refer to [25] for a discussion of the aforementioned biogeographic hypothesis. Together these taxa seem to highlight dispersal from southern Laramidia to northern Laramidia, with increasing disparity in cranial ornamentation in northern Laramidian forms and relatively conservatism in cranial architecture in southern forms until the appearance of *Nasutoceratops titusi* (UMNH VP 16800) during the late Campanian [8].

The pre-orbital region of the skull was not preserved, thereby limiting detailed morphological comparisons of *Machairoceratops* with *Diabloceratops* and other centrosaurines. Nonetheless, the preserved material of *Machairoceratops* includes characters that allow its confident placement within Centrosaurinae. In addition, other features link it with the stratigraphically lower *Diabloceratops eatoni* from the lower middle member of the Wahweap Formation and to a yet undescribed centrosaurine from the lower member of the Wahweap Formation (Wahweap centrosaurine A [UMNH VP 20600] of [6]). Among these are the presence of robust, elongate supraorbital horns, a triangular (rather than round) parietosquamosal frill, and two spike-like epiparietal loci (p1) adornments on either side of a midline parietal embayment [5]. However, *Machairoceratops* differs from *Diabloceratops* in a number of key features, including: a fan-shaped, subrectangular (rather than rectangular) stepped squamosal, a larger overall step of the squamosal, epiparietals that are anterodorsally (rather than laterally) curved, and a posteromedially oriented sulcus running the length of the posterior surface of epiparietal locus (p1). In fact, this latter feature is autapomorphic and distinguishes *Machairoceratops* from all other known centrosaurines.

*Machairoceratops* shares morphological features of the squamosal with yet another unnamed and stratigraphically lower taxon from the Wahweap Formation (Nipple Butte skull [UMNH VP 16704]) [5]. Specifically, the squamosals of each taxon are comparable in overall shape, being subrectangular rather than rectangular, suggesting that there may be at least two distinct lineages of centrosaurines through the Wahweap Formation (i.e., a *Diabloceratops* lineage and a *Machairoceratop*s lineage). Additionally, the variation observed among the squamosals of these three taxa (i.e., *Diabloceratops*, *Machairoceratops*, and UMNH VP 16704) falls well outside the expected intraspecific variation for *Diabloceratops* based on variation known for other ceratopsids [53, 54]. Moreover, the temporal separation among the aforementioned taxa is similarly outside the expected temporal duration given known species turnover rates for other ceratopsians [55, 56]. Taken together, these attributes suggest the presence of a divergent centrosaurine ceratopsid from the upper member of the Wahweap Formation, thereby increasing the known centrosaurine diversity from the southern portion of Laramidia during the late Campanian.

The discovery, phylogenetic placement, and stratigraphic occurrence of *Machairoceratops* from the Wahweap Formation further supports the hypothesis of ceratopsian dinosaur provincialism in Laramidia by indicating the presence of two distinct clades of contemporaneous centrosaurines that were geographically isolated for at least a million years. For example, *Coronosaurus brinkmani* (TMP 2002.68.1–3), a characteristically short-horned, northern distributed centrosaurine temporally overlaps with *Machairoceratops cronusi* (UMNH VP 20550), a characteristically long-horned, southern distributed centrosaurine bolstering the hypothesis of disparate, latitudinally-arrayed groups of contemporaneous centrosaurines occupying Laramidia (Fig 7) [49].

## Conclusions

New ceratopsian dinosaur material (UMNH VP 20550) recovered from the upper member of the Wahweap Formation is here used to erect a new taxon, *Machairoceratops cronusi* gen. et sp. nov., which can be confidently placed as an early-branching centrosaurine established on both a parsimony-based analysis and a Bayesian analysis. One autapomorphic character of the new taxon (i.e., epiparietal (p1) ornamentation) expands known epiparietal disparity in ceratopsid dinosaurs. Considered together, the phylogenetic, stratigraphic, and morphologic evidence distinguishes *Machairoceratops* from all other centrosaurine dinosaurs, and increases the known ceratopsian diversity in the southern portion of Laramidia.

## Supporting Information

S1 Fig Resultant 50% majority rule consensus tree from the parsimony analysis. Results of the maximum parsimony analysis reported here as the 50% majority rule consensus of 1194 most parsimonious trees (tree length = 160, CI = 0.675, RI, 0.818, RCI = 0.552) of an analysis of 101 characters arrayed across 26 ceratopsian taxa. Numbers in node boxes indicate frequency (in percent) of node configuration found for all solutions in the analysis.
(TIF)

S1 File Appendix A: Specific specimen sources used for character scoring. (DOCX)

S2 File Appendix B: Character list used for phylogenetic analysis. (DOCX)

S3 File Appendix C: Parameter setting used in MrBayes. (DOCX)

S4 File 3D-PDF of the braincase of *Machairoceratops cronusi* (UMNH VP 20550) gen. et sp. nov., based on CT scan data in S5 File. (PDF)

S5 File Individual CT scan slice data of the braincase of *Machairoceratops cronusi* (UMNH VP 20550) gen. et sp. nov. (ZIP)

S1 Table Character-taxon matrix for phylogenetic analysis. (DOCX)
