# Supplementary figures and images for: A New Centrosaurine Ceratopsid, Machairoceratops cronusi gen et sp. nov., from the Upper Sand Member of the Wahweap Formation (Middle Campanian), Southern Utah (part 1 of 3)
Source: PLoS One. 2016 May 18;11(5):e0154403. doi: 10.1371/journal.pone.0154403 (PMC4871575; doi:10.1371/journal.pone.0154403)

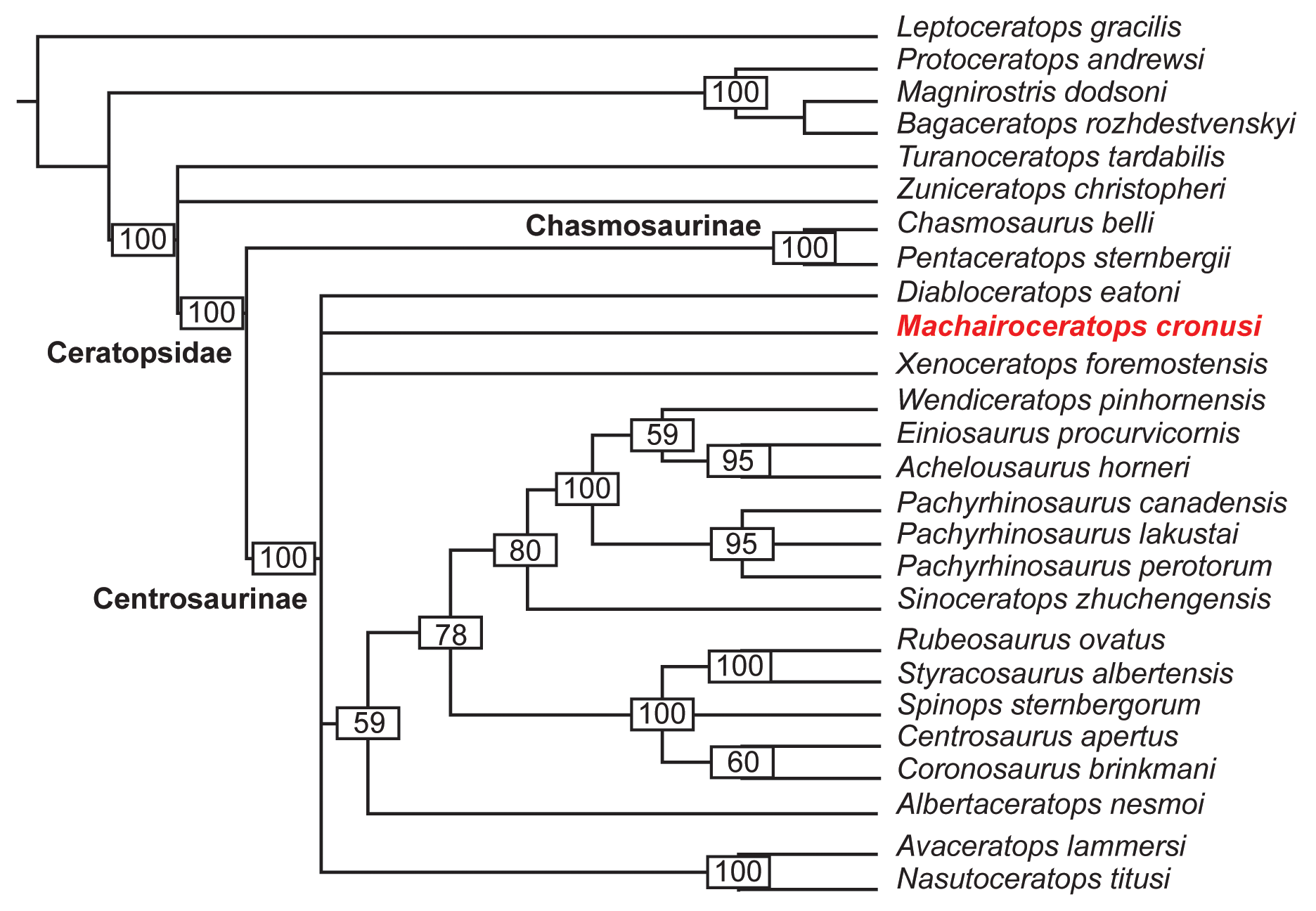

Supplement: S1 Fig — Results of the maximum parsimony analysis reported here as the 50% majority rule consensus of 1194 most parsimonious trees (tree length = 160, CI = 0.675, RI, 0.818, RCI = 0.552) of an analysis of 101 characters arrayed across 26 ceratopsian taxa. Numbers in node boxes indicate frequency (in percent) of node configuration found for all solutions in the analysis. (TIF) [file pone.0154403.s001.tif]

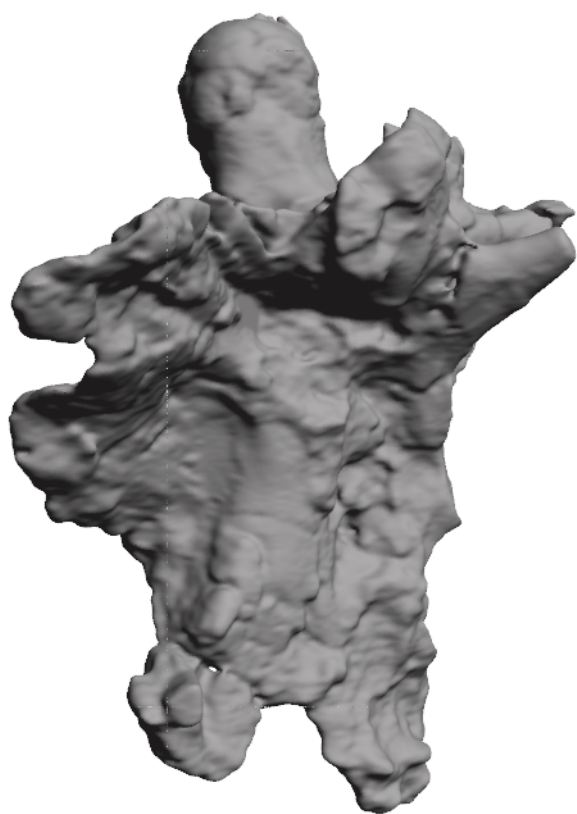

Supplement: S4 File — (PDF) [file pone.0154403.s005.pdf]

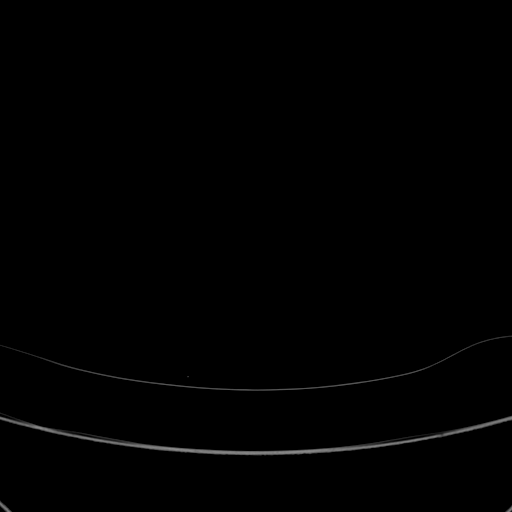

Supplement: S5 File — (ZIP) [file pone.0154403.s006.zip › S2_Files/WWCERATBC.Ser2.Img1.tif]

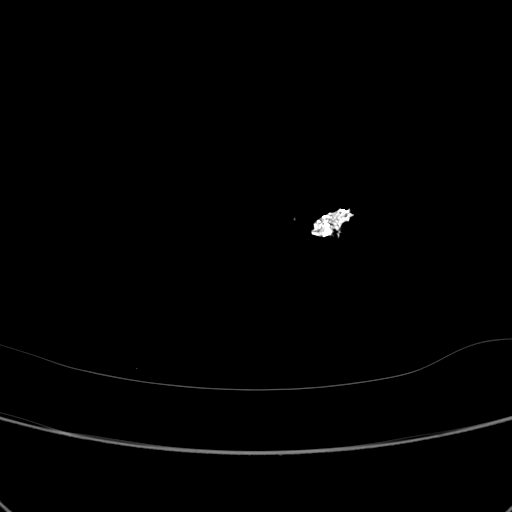

Supplement: S5 File — (ZIP) [file pone.0154403.s006.zip › S2_Files/WWCERATBC.Ser2.Img10.tif]

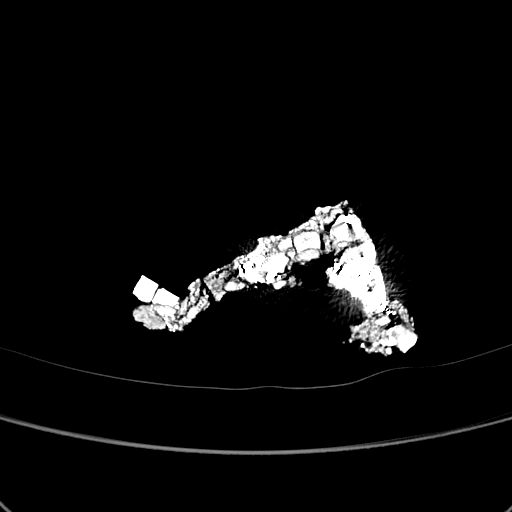

Supplement: S5 File — (ZIP) [file pone.0154403.s006.zip › S2_Files/WWCERATBC.Ser2.Img100.tif]

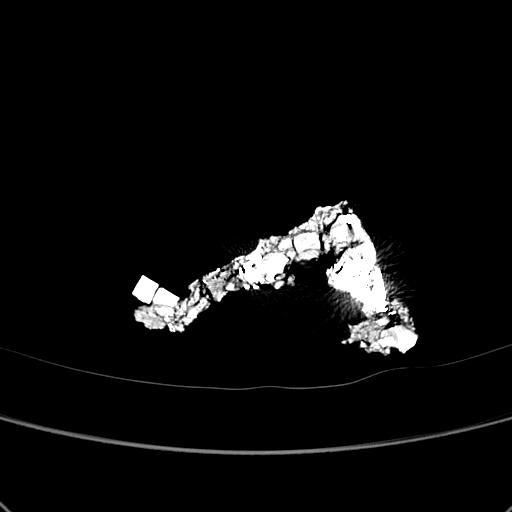

Supplement: S5 File — (ZIP) [file pone.0154403.s006.zip › S2_Files/WWCERATBC.Ser2.Img101.tif]

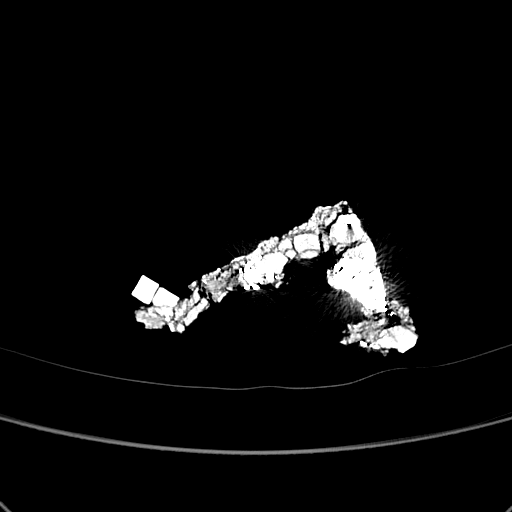

Supplement: S5 File — (ZIP) [file pone.0154403.s006.zip › S2_Files/WWCERATBC.Ser2.Img102.tif]

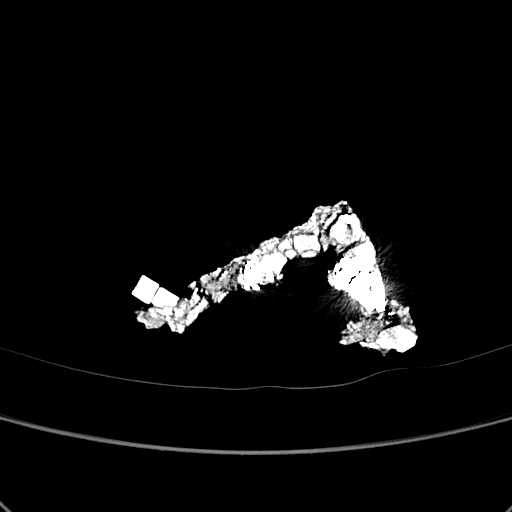

Supplement: S5 File — (ZIP) [file pone.0154403.s006.zip › S2_Files/WWCERATBC.Ser2.Img103.tif]

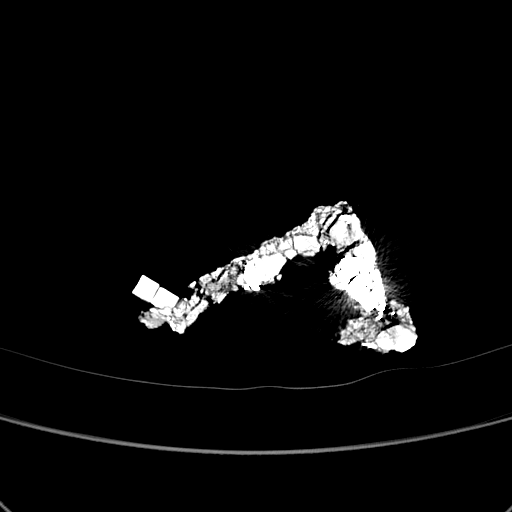

Supplement: S5 File — (ZIP) [file pone.0154403.s006.zip › S2_Files/WWCERATBC.Ser2.Img104.tif]

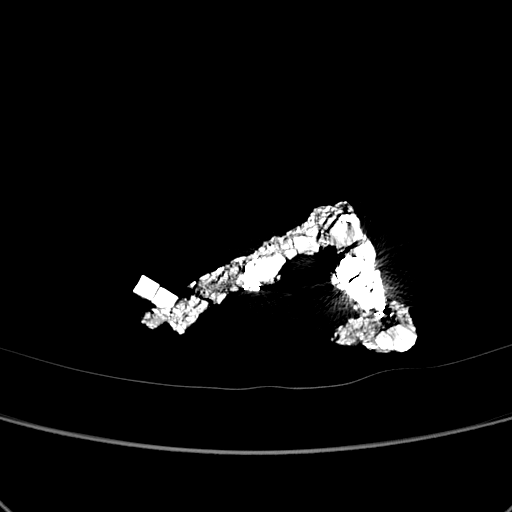

Supplement: S5 File — (ZIP) [file pone.0154403.s006.zip › S2_Files/WWCERATBC.Ser2.Img105.tif]

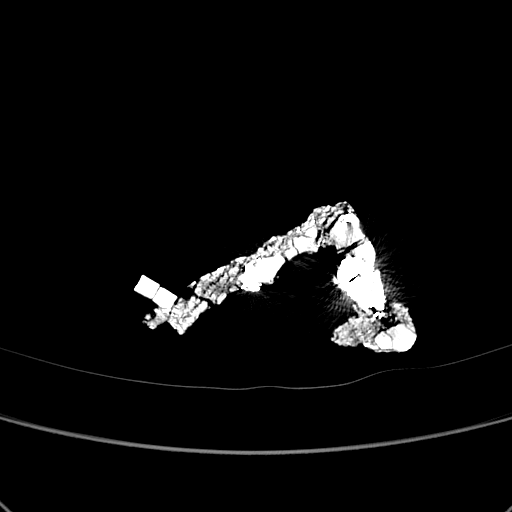

Supplement: S5 File — (ZIP) [file pone.0154403.s006.zip › S2_Files/WWCERATBC.Ser2.Img106.tif]

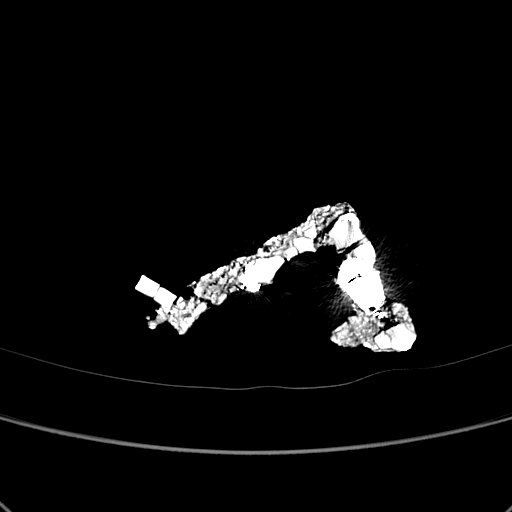

Supplement: S5 File — (ZIP) [file pone.0154403.s006.zip › S2_Files/WWCERATBC.Ser2.Img107.tif]

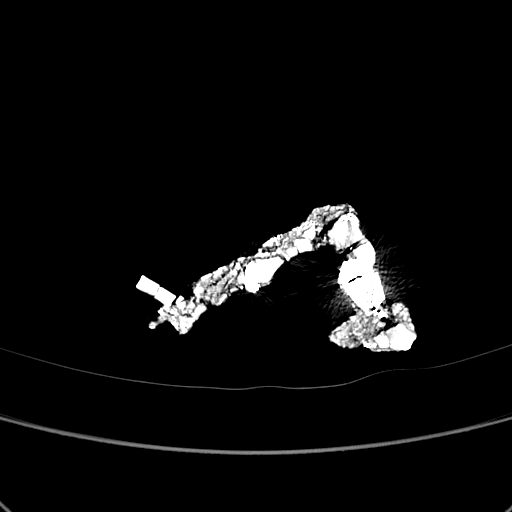

Supplement: S5 File — (ZIP) [file pone.0154403.s006.zip › S2_Files/WWCERATBC.Ser2.Img108.tif]

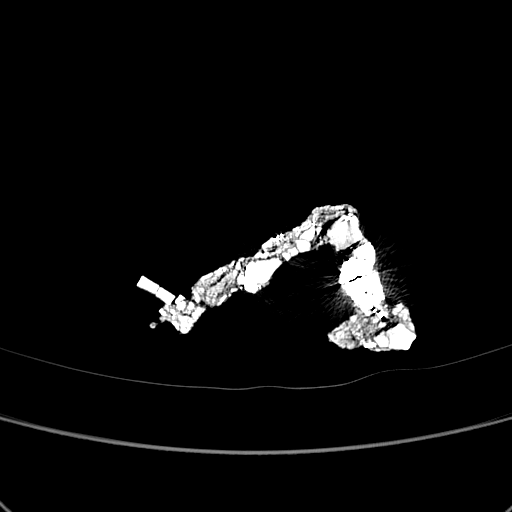

Supplement: S5 File — (ZIP) [file pone.0154403.s006.zip › S2_Files/WWCERATBC.Ser2.Img109.tif]

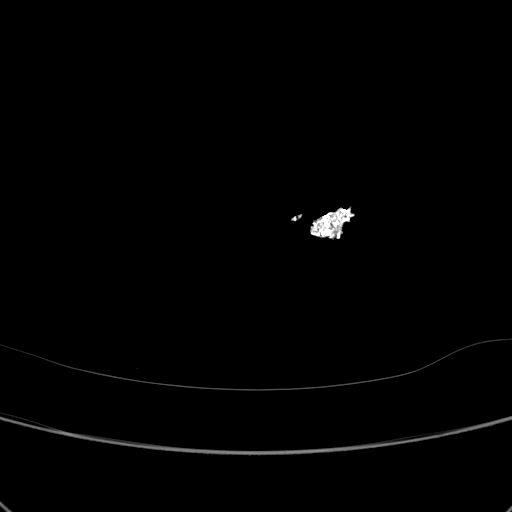

Supplement: S5 File — (ZIP) [file pone.0154403.s006.zip › S2_Files/WWCERATBC.Ser2.Img11.tif]

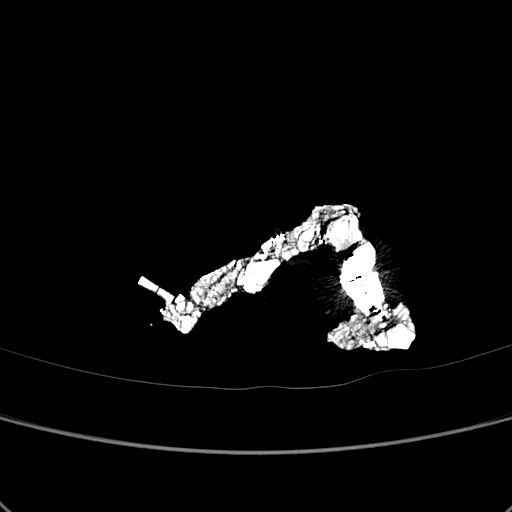

Supplement: S5 File — (ZIP) [file pone.0154403.s006.zip › S2_Files/WWCERATBC.Ser2.Img110.tif]

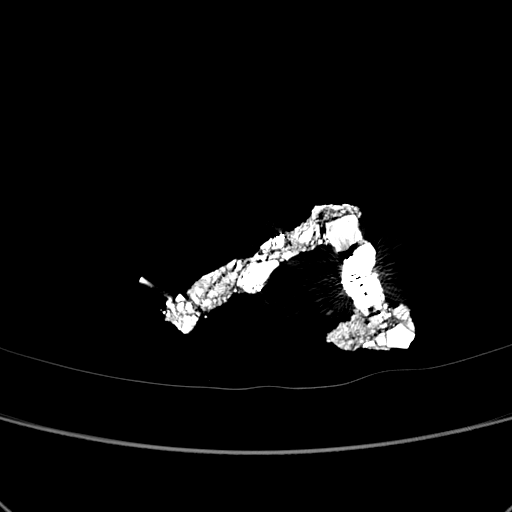

Supplement: S5 File — (ZIP) [file pone.0154403.s006.zip › S2_Files/WWCERATBC.Ser2.Img111.tif]

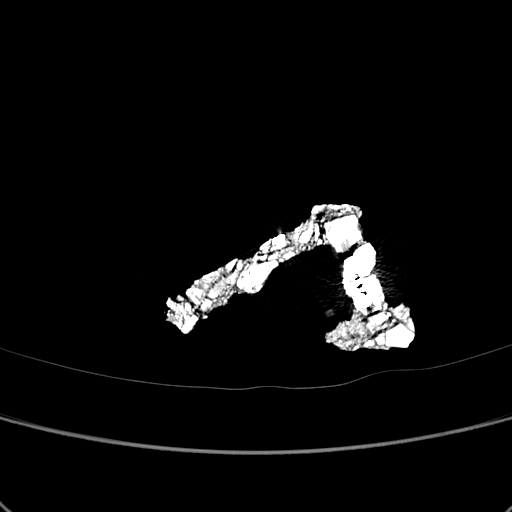

Supplement: S5 File — (ZIP) [file pone.0154403.s006.zip › S2_Files/WWCERATBC.Ser2.Img112.tif]

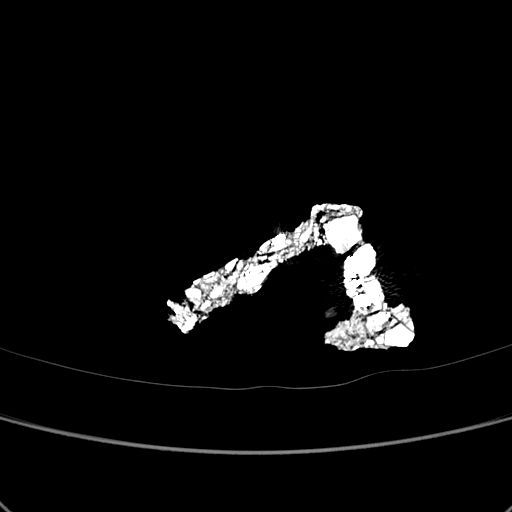

Supplement: S5 File — (ZIP) [file pone.0154403.s006.zip › S2_Files/WWCERATBC.Ser2.Img113.tif]

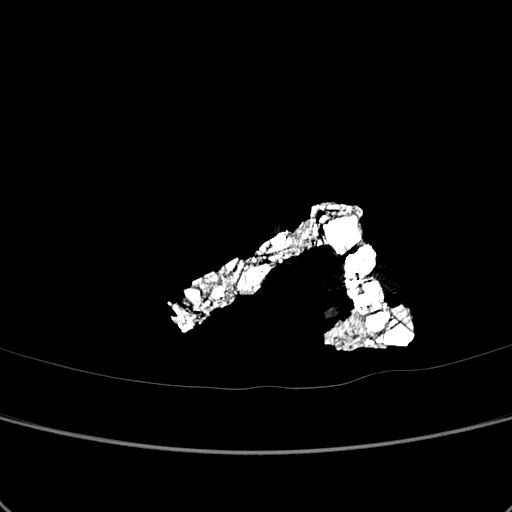

Supplement: S5 File — (ZIP) [file pone.0154403.s006.zip › S2_Files/WWCERATBC.Ser2.Img114.tif]

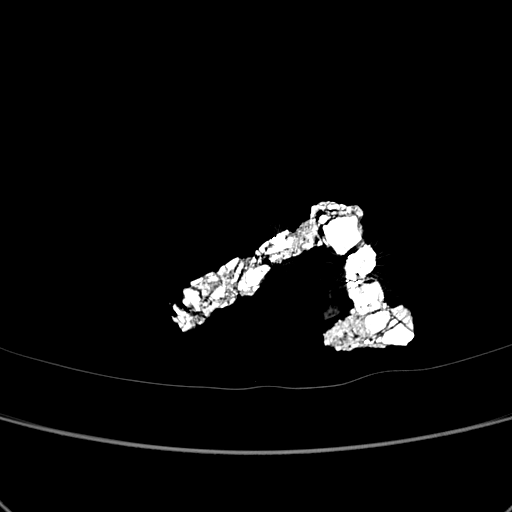

Supplement: S5 File — (ZIP) [file pone.0154403.s006.zip › S2_Files/WWCERATBC.Ser2.Img115.tif]

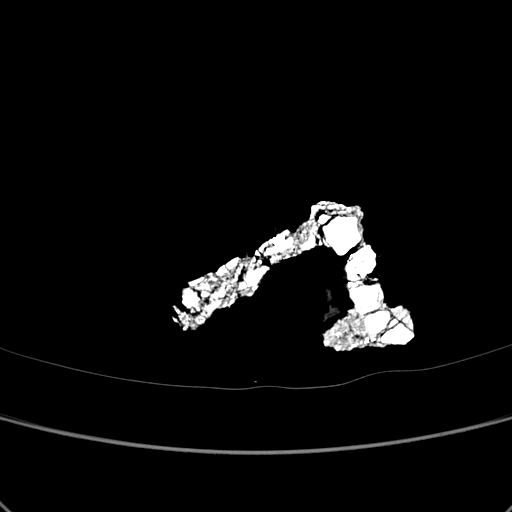

Supplement: S5 File — (ZIP) [file pone.0154403.s006.zip › S2_Files/WWCERATBC.Ser2.Img116.tif]

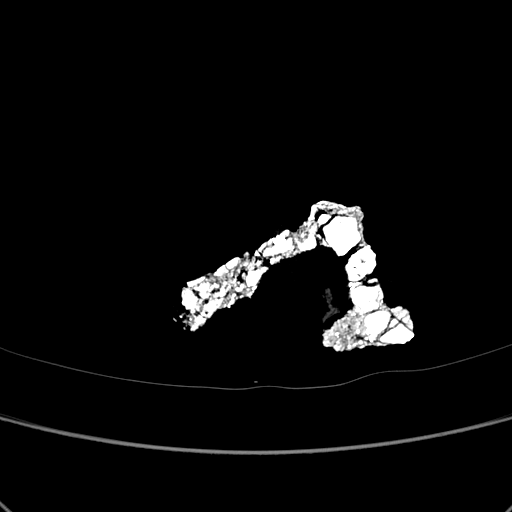

Supplement: S5 File — (ZIP) [file pone.0154403.s006.zip › S2_Files/WWCERATBC.Ser2.Img117.tif]

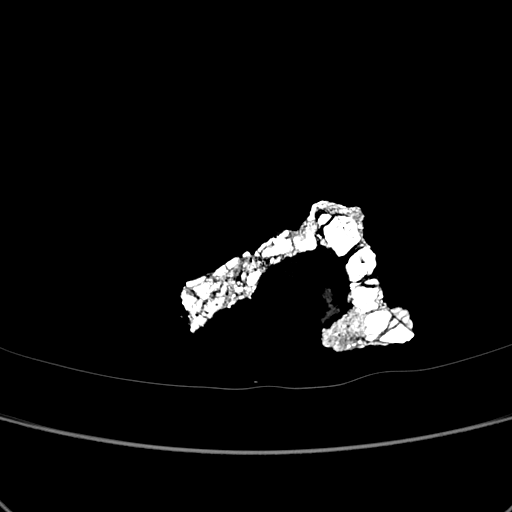

Supplement: S5 File — (ZIP) [file pone.0154403.s006.zip › S2_Files/WWCERATBC.Ser2.Img118.tif]

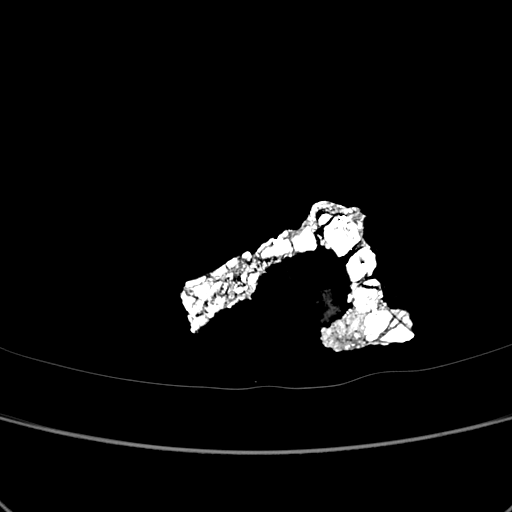

Supplement: S5 File — (ZIP) [file pone.0154403.s006.zip › S2_Files/WWCERATBC.Ser2.Img119.tif]

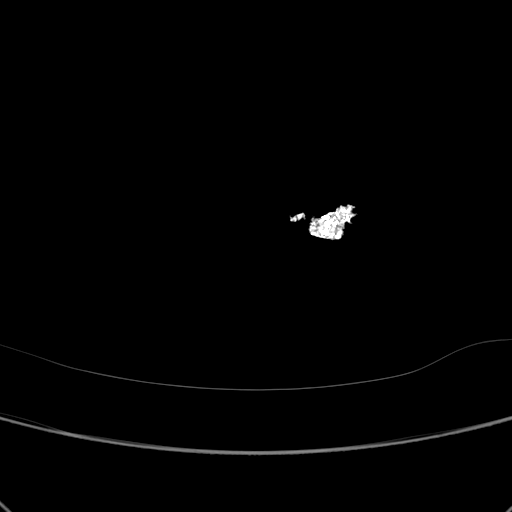

Supplement: S5 File — (ZIP) [file pone.0154403.s006.zip › S2_Files/WWCERATBC.Ser2.Img12.tif]

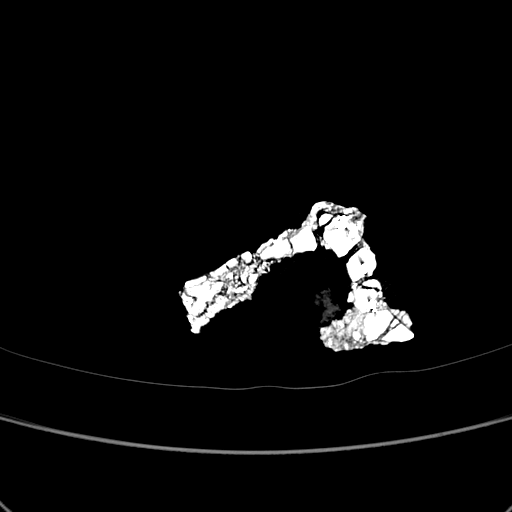

Supplement: S5 File — (ZIP) [file pone.0154403.s006.zip › S2_Files/WWCERATBC.Ser2.Img120.tif]

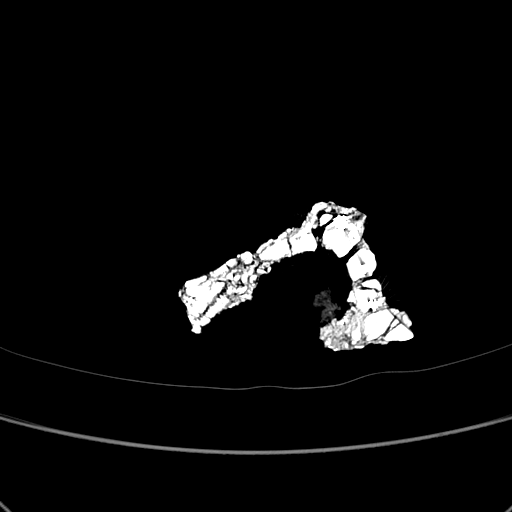

Supplement: S5 File — (ZIP) [file pone.0154403.s006.zip › S2_Files/WWCERATBC.Ser2.Img121.tif]

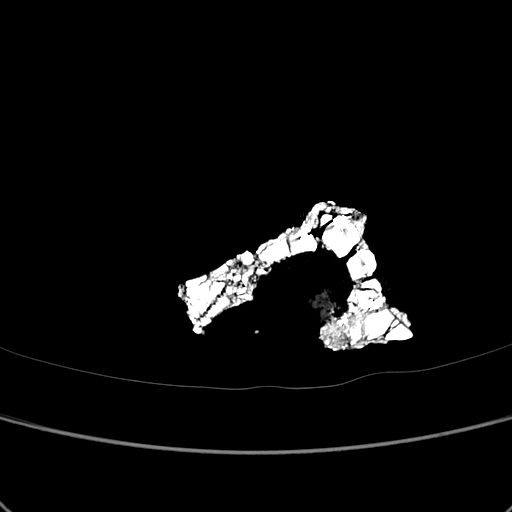

Supplement: S5 File — (ZIP) [file pone.0154403.s006.zip › S2_Files/WWCERATBC.Ser2.Img122.tif]

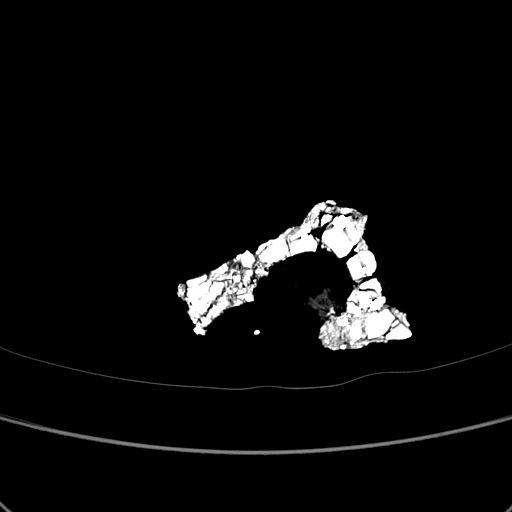

Supplement: S5 File — (ZIP) [file pone.0154403.s006.zip › S2_Files/WWCERATBC.Ser2.Img123.tif]

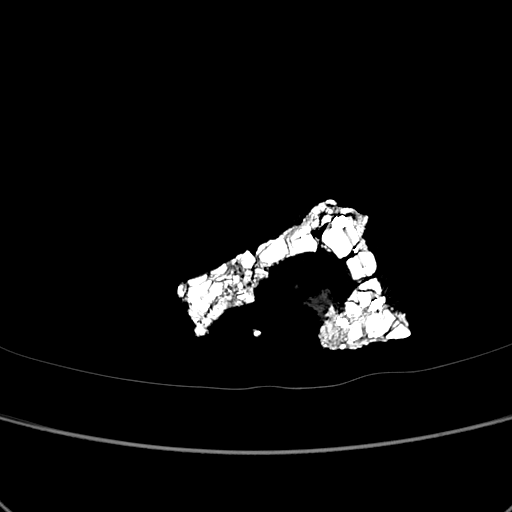

Supplement: S5 File — (ZIP) [file pone.0154403.s006.zip › S2_Files/WWCERATBC.Ser2.Img124.tif]

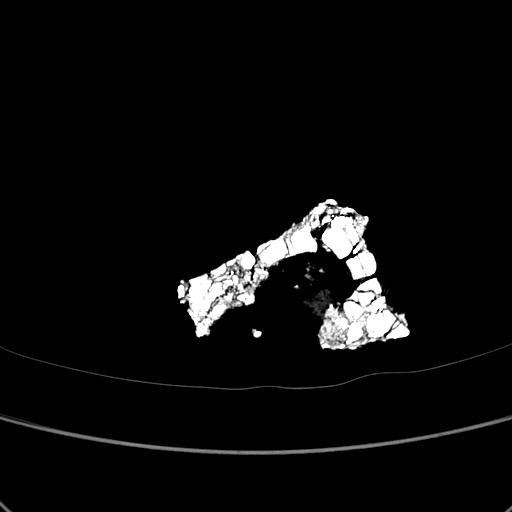

Supplement: S5 File — (ZIP) [file pone.0154403.s006.zip › S2_Files/WWCERATBC.Ser2.Img125.tif]

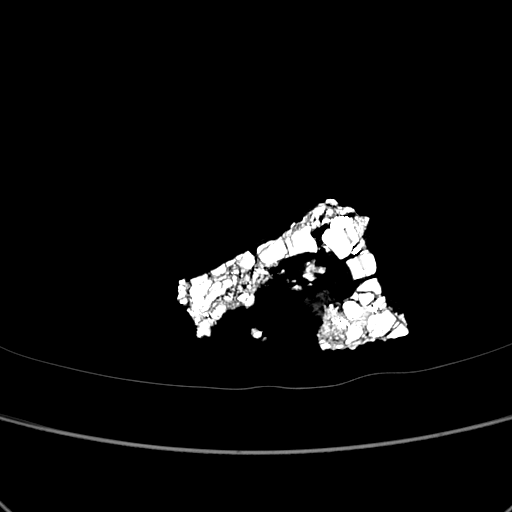

Supplement: S5 File — (ZIP) [file pone.0154403.s006.zip › S2_Files/WWCERATBC.Ser2.Img126.tif]

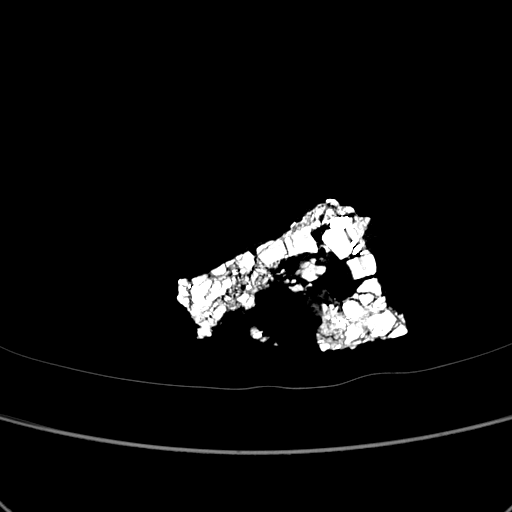

Supplement: S5 File — (ZIP) [file pone.0154403.s006.zip › S2_Files/WWCERATBC.Ser2.Img127.tif]

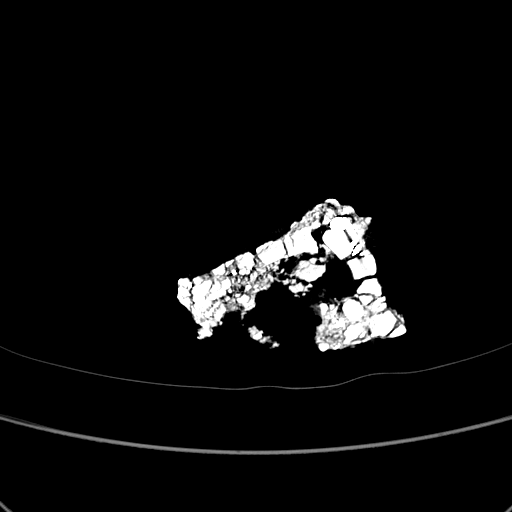

Supplement: S5 File — (ZIP) [file pone.0154403.s006.zip › S2_Files/WWCERATBC.Ser2.Img128.tif]

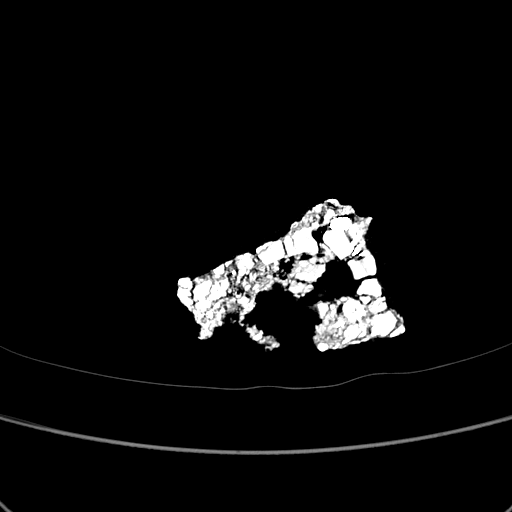

Supplement: S5 File — (ZIP) [file pone.0154403.s006.zip › S2_Files/WWCERATBC.Ser2.Img129.tif]

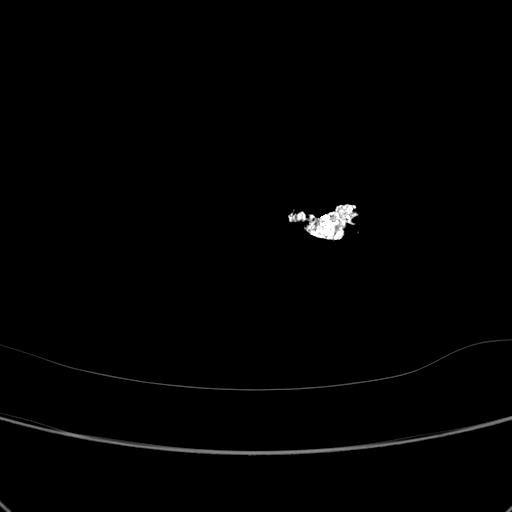

Supplement: S5 File — (ZIP) [file pone.0154403.s006.zip › S2_Files/WWCERATBC.Ser2.Img13.tif]

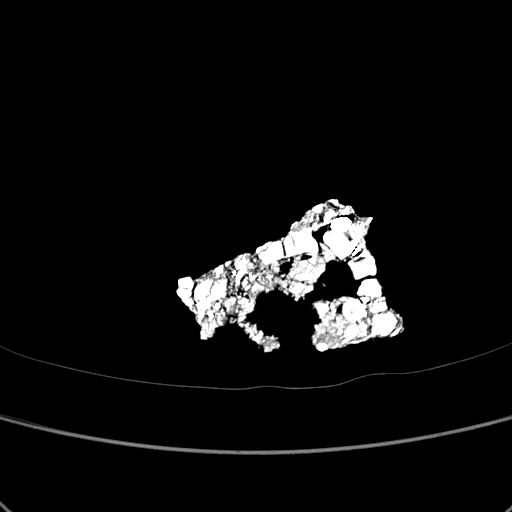

Supplement: S5 File — (ZIP) [file pone.0154403.s006.zip › S2_Files/WWCERATBC.Ser2.Img130.tif]

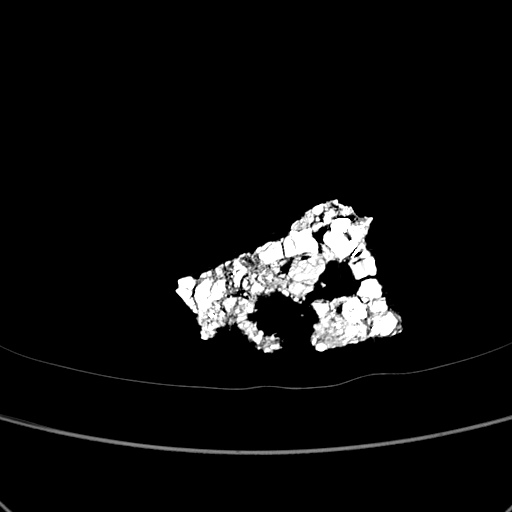

Supplement: S5 File — (ZIP) [file pone.0154403.s006.zip › S2_Files/WWCERATBC.Ser2.Img131.tif]

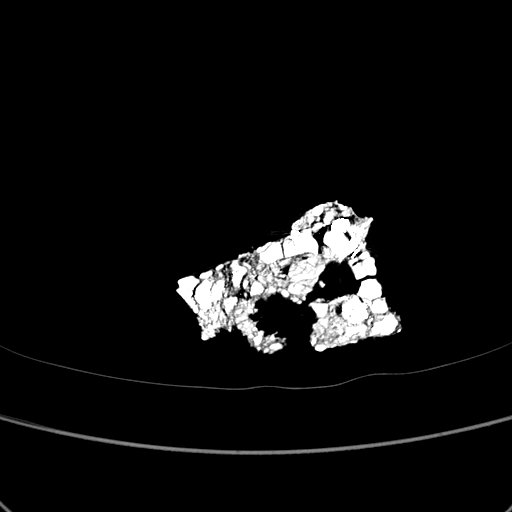

Supplement: S5 File — (ZIP) [file pone.0154403.s006.zip › S2_Files/WWCERATBC.Ser2.Img132.tif]

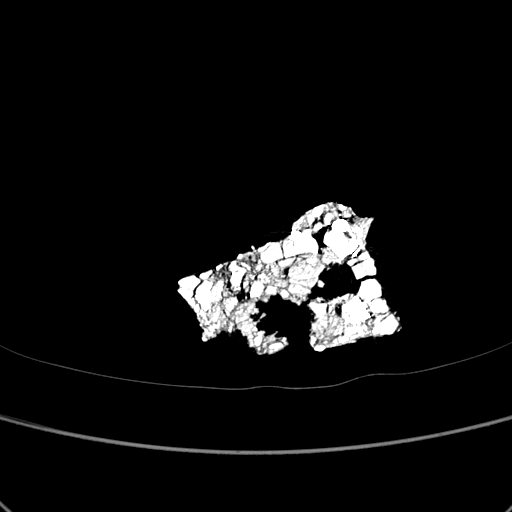

Supplement: S5 File — (ZIP) [file pone.0154403.s006.zip › S2_Files/WWCERATBC.Ser2.Img133.tif]

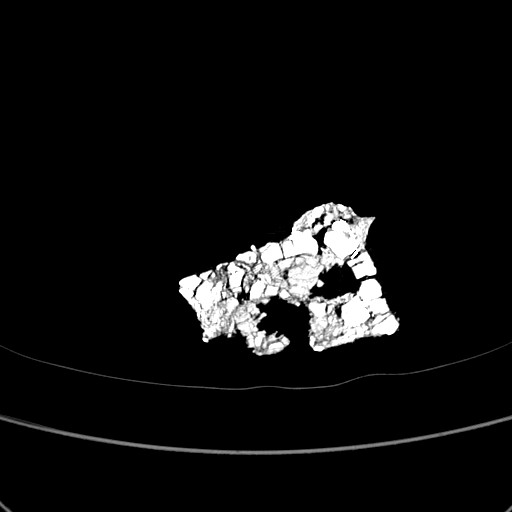

Supplement: S5 File — (ZIP) [file pone.0154403.s006.zip › S2_Files/WWCERATBC.Ser2.Img134.tif]

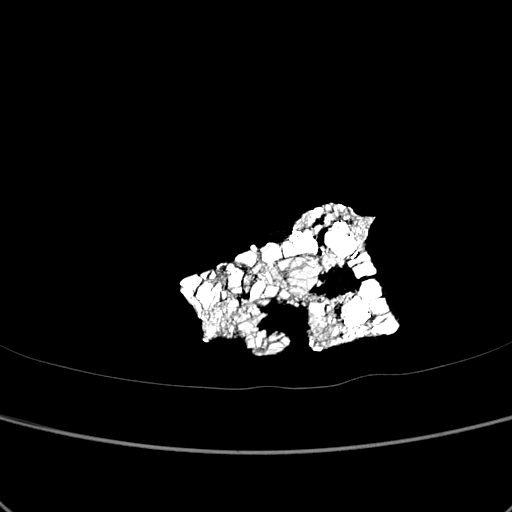

Supplement: S5 File — (ZIP) [file pone.0154403.s006.zip › S2_Files/WWCERATBC.Ser2.Img135.tif]

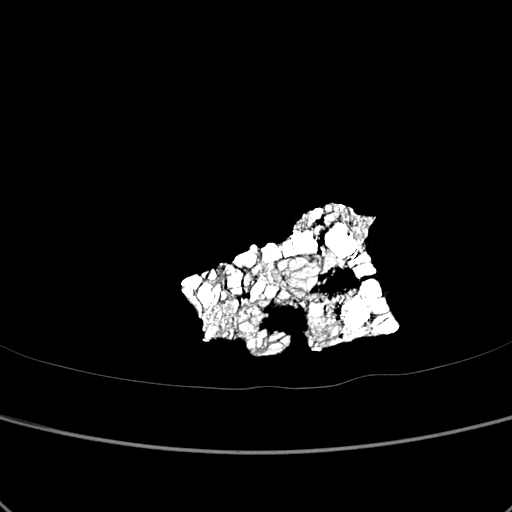

Supplement: S5 File — (ZIP) [file pone.0154403.s006.zip › S2_Files/WWCERATBC.Ser2.Img136.tif]

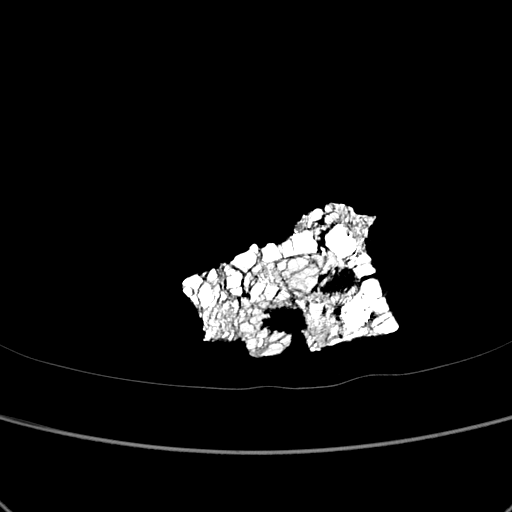

Supplement: S5 File — (ZIP) [file pone.0154403.s006.zip › S2_Files/WWCERATBC.Ser2.Img137.tif]

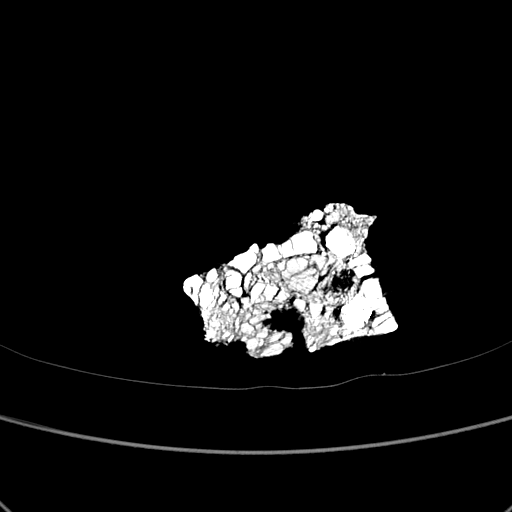

Supplement: S5 File — (ZIP) [file pone.0154403.s006.zip › S2_Files/WWCERATBC.Ser2.Img138.tif]

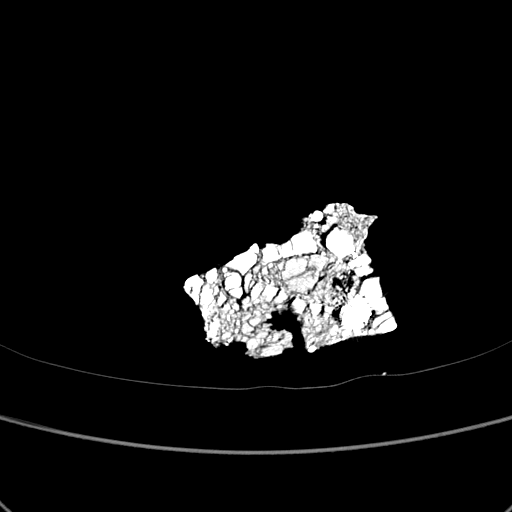

Supplement: S5 File — (ZIP) [file pone.0154403.s006.zip › S2_Files/WWCERATBC.Ser2.Img139.tif]

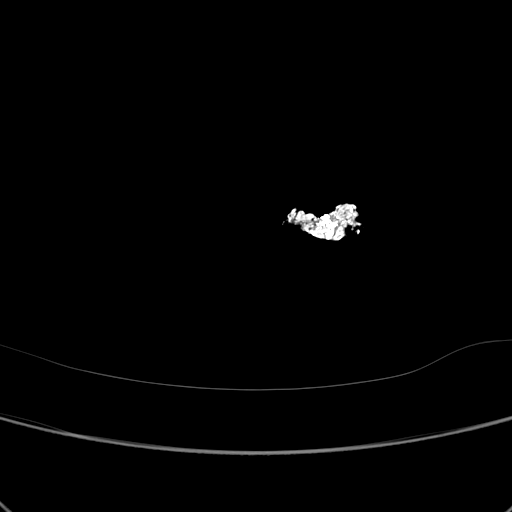

Supplement: S5 File — (ZIP) [file pone.0154403.s006.zip › S2_Files/WWCERATBC.Ser2.Img14.tif]

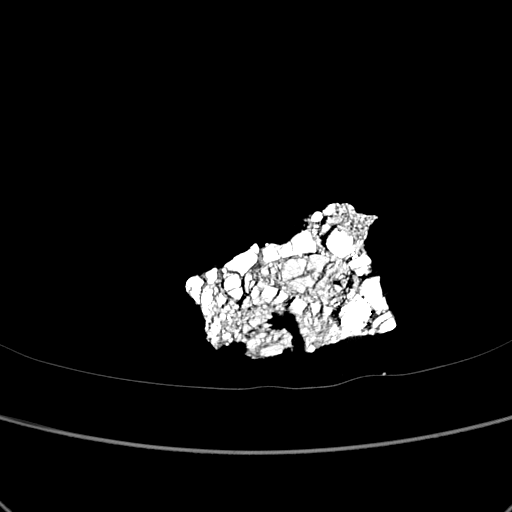

Supplement: S5 File — (ZIP) [file pone.0154403.s006.zip › S2_Files/WWCERATBC.Ser2.Img140.tif]

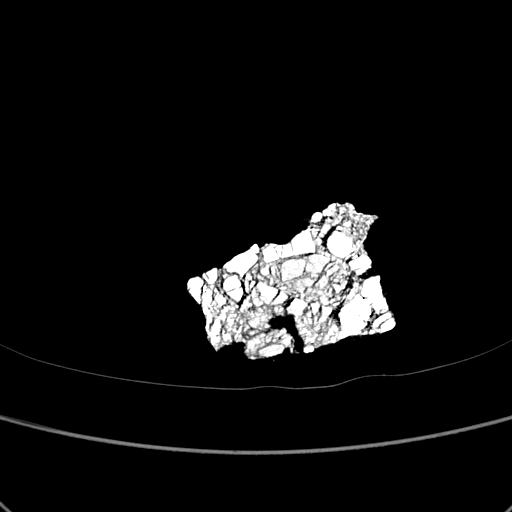

Supplement: S5 File — (ZIP) [file pone.0154403.s006.zip › S2_Files/WWCERATBC.Ser2.Img141.tif]

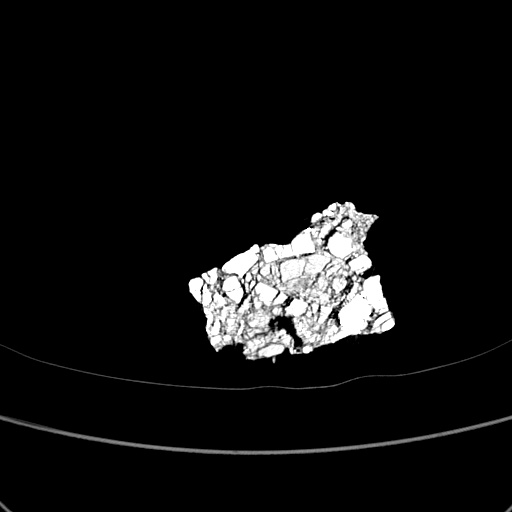

Supplement: S5 File — (ZIP) [file pone.0154403.s006.zip › S2_Files/WWCERATBC.Ser2.Img142.tif]

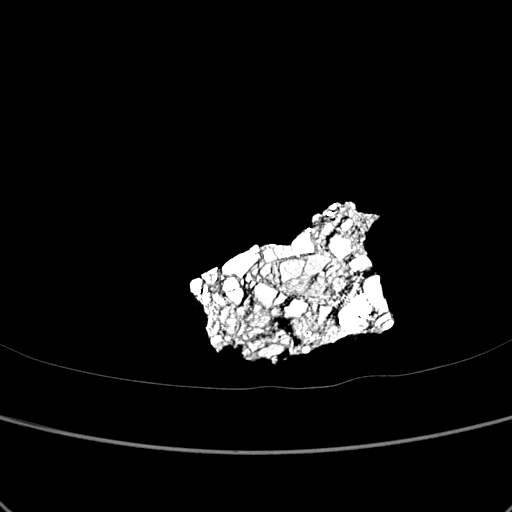

Supplement: S5 File — (ZIP) [file pone.0154403.s006.zip › S2_Files/WWCERATBC.Ser2.Img143.tif]

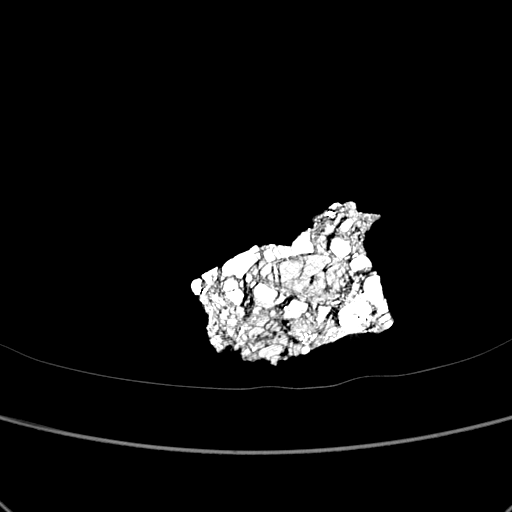

Supplement: S5 File — (ZIP) [file pone.0154403.s006.zip › S2_Files/WWCERATBC.Ser2.Img144.tif]

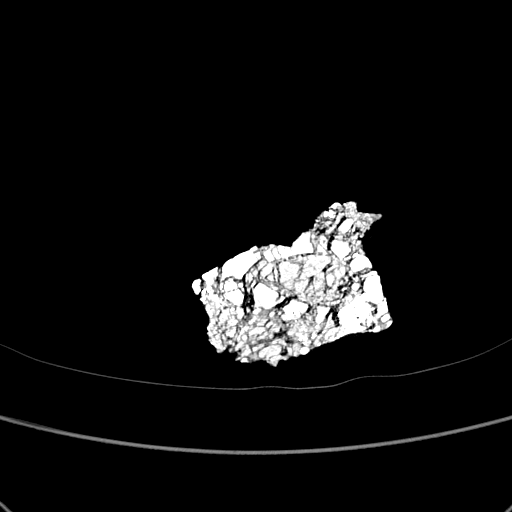

Supplement: S5 File — (ZIP) [file pone.0154403.s006.zip › S2_Files/WWCERATBC.Ser2.Img145.tif]

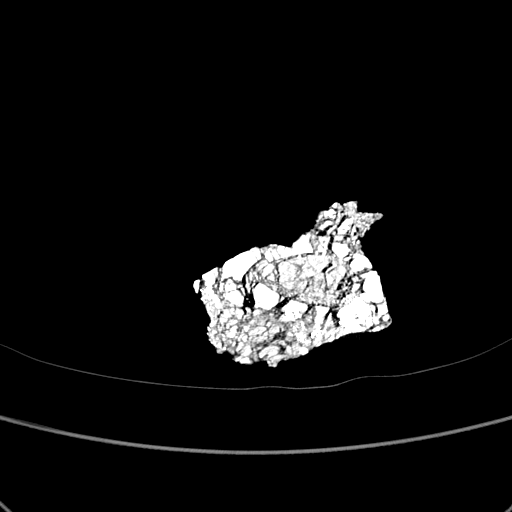

Supplement: S5 File — (ZIP) [file pone.0154403.s006.zip › S2_Files/WWCERATBC.Ser2.Img146.tif]

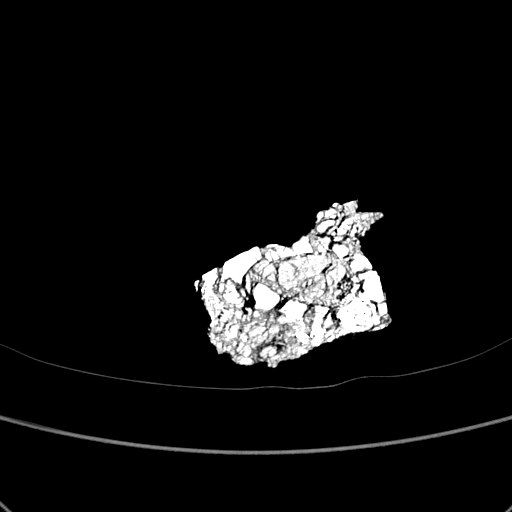

Supplement: S5 File — (ZIP) [file pone.0154403.s006.zip › S2_Files/WWCERATBC.Ser2.Img147.tif]

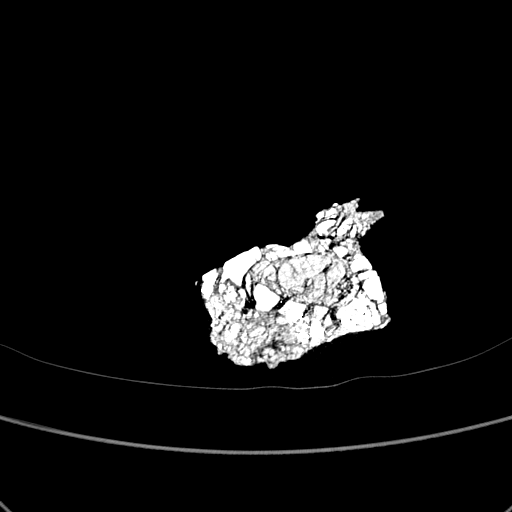

Supplement: S5 File — (ZIP) [file pone.0154403.s006.zip › S2_Files/WWCERATBC.Ser2.Img148.tif]

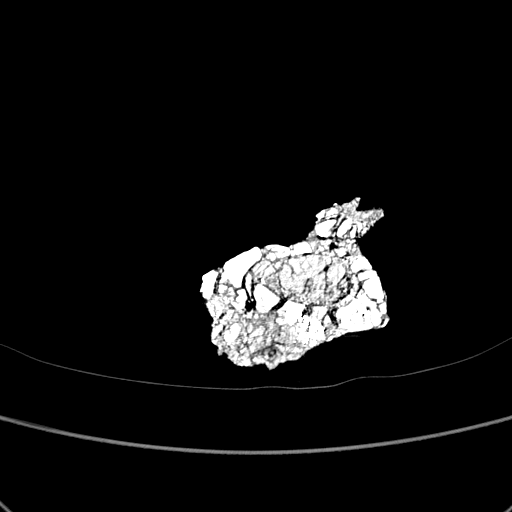

Supplement: S5 File — (ZIP) [file pone.0154403.s006.zip › S2_Files/WWCERATBC.Ser2.Img149.tif]

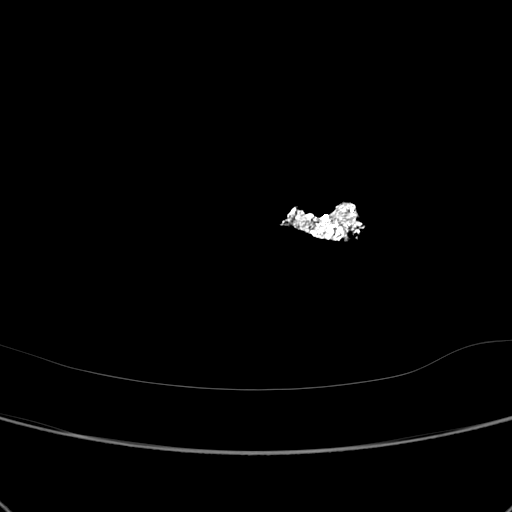

Supplement: S5 File — (ZIP) [file pone.0154403.s006.zip › S2_Files/WWCERATBC.Ser2.Img15.tif]

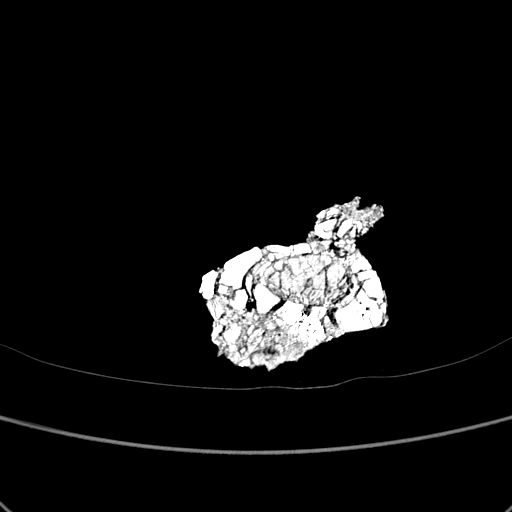

Supplement: S5 File — (ZIP) [file pone.0154403.s006.zip › S2_Files/WWCERATBC.Ser2.Img150.tif]

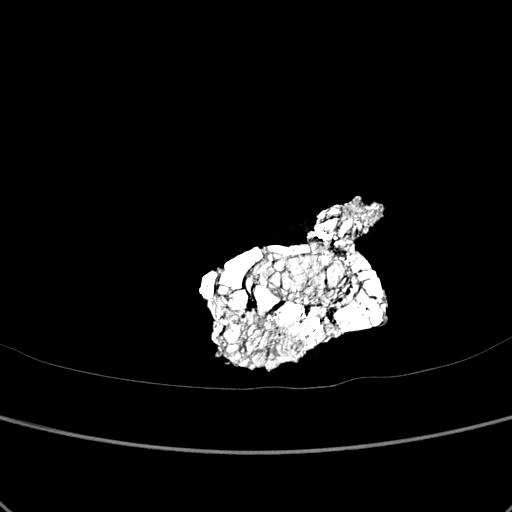

Supplement: S5 File — (ZIP) [file pone.0154403.s006.zip › S2_Files/WWCERATBC.Ser2.Img151.tif]

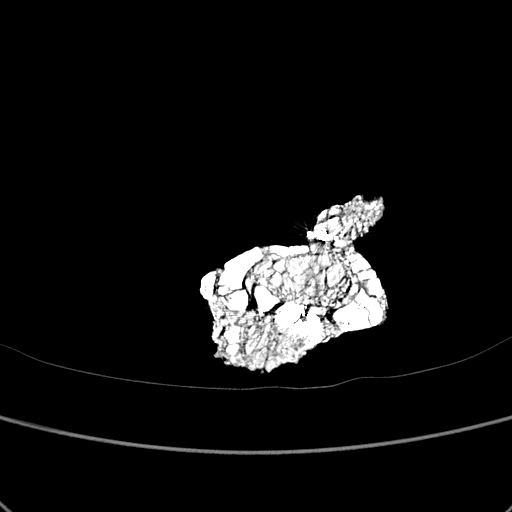

Supplement: S5 File — (ZIP) [file pone.0154403.s006.zip › S2_Files/WWCERATBC.Ser2.Img152.tif]

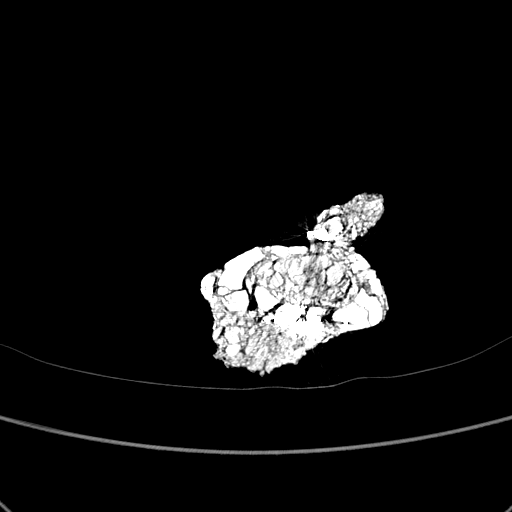

Supplement: S5 File — (ZIP) [file pone.0154403.s006.zip › S2_Files/WWCERATBC.Ser2.Img153.tif]

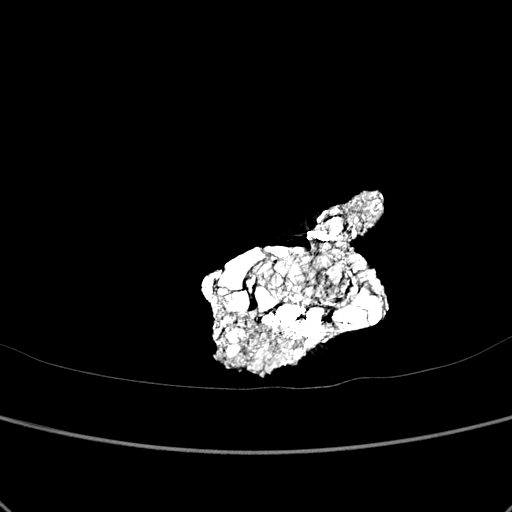

Supplement: S5 File — (ZIP) [file pone.0154403.s006.zip › S2_Files/WWCERATBC.Ser2.Img154.tif]

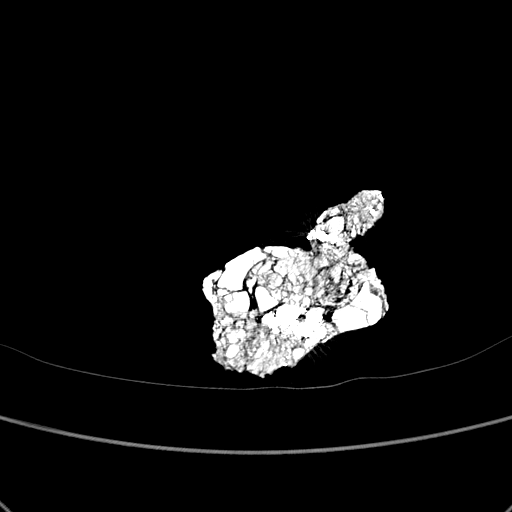

Supplement: S5 File — (ZIP) [file pone.0154403.s006.zip › S2_Files/WWCERATBC.Ser2.Img155.tif]

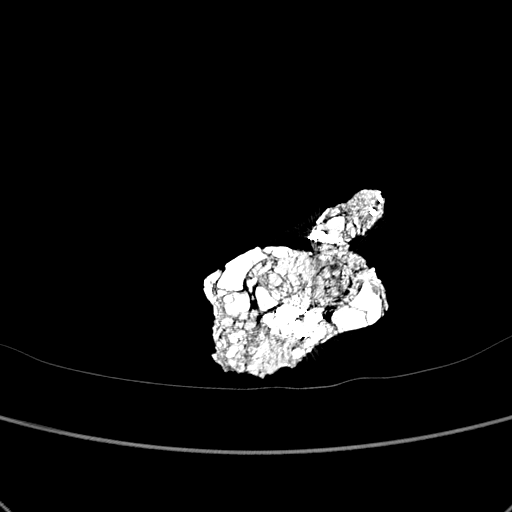

Supplement: S5 File — (ZIP) [file pone.0154403.s006.zip › S2_Files/WWCERATBC.Ser2.Img156.tif]

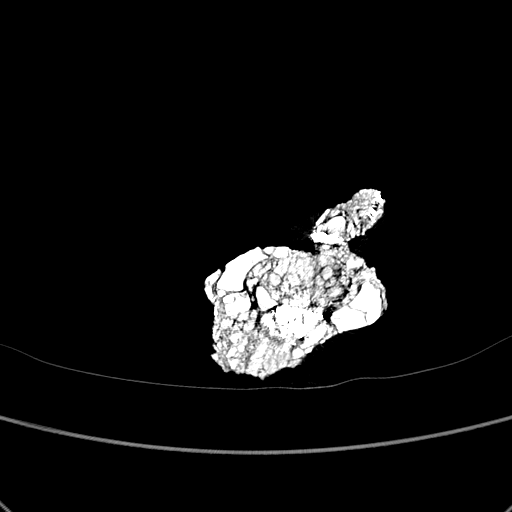

Supplement: S5 File — (ZIP) [file pone.0154403.s006.zip › S2_Files/WWCERATBC.Ser2.Img157.tif]

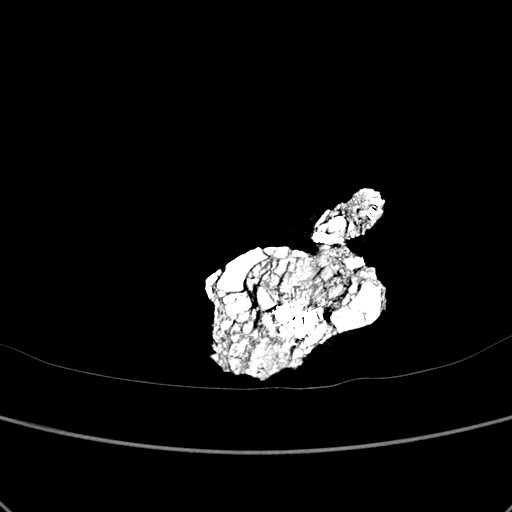

Supplement: S5 File — (ZIP) [file pone.0154403.s006.zip › S2_Files/WWCERATBC.Ser2.Img158.tif]

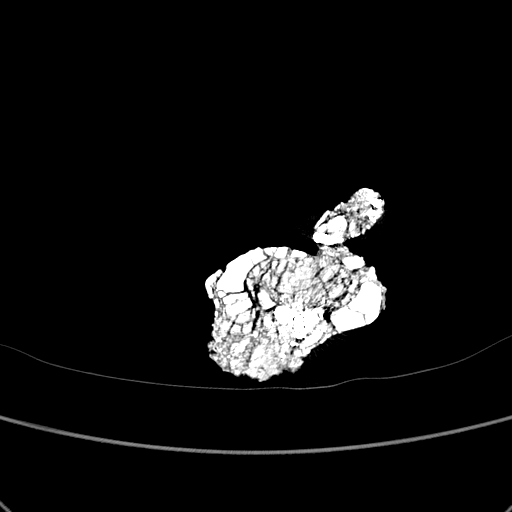

Supplement: S5 File — (ZIP) [file pone.0154403.s006.zip › S2_Files/WWCERATBC.Ser2.Img159.tif]

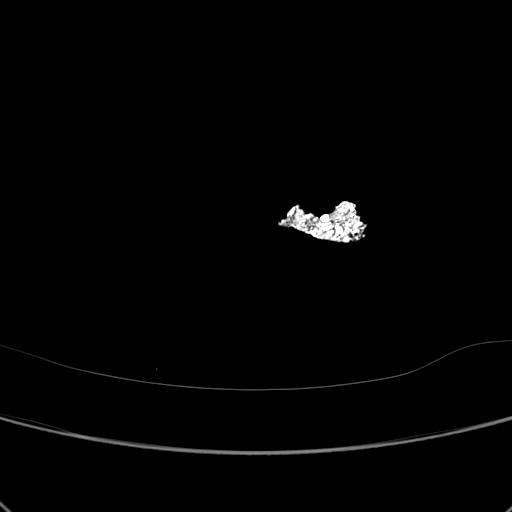

Supplement: S5 File — (ZIP) [file pone.0154403.s006.zip › S2_Files/WWCERATBC.Ser2.Img16.tif]

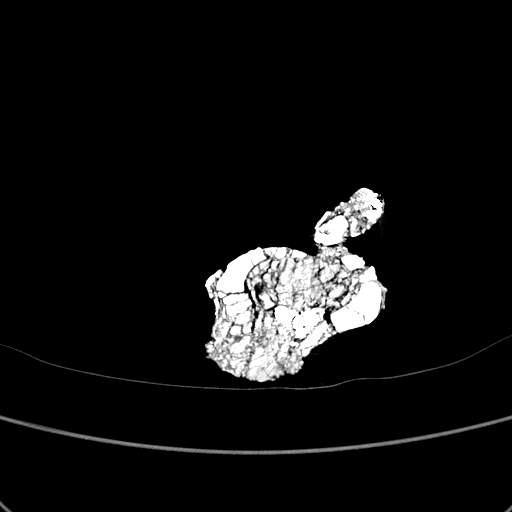

Supplement: S5 File — (ZIP) [file pone.0154403.s006.zip › S2_Files/WWCERATBC.Ser2.Img160.tif]

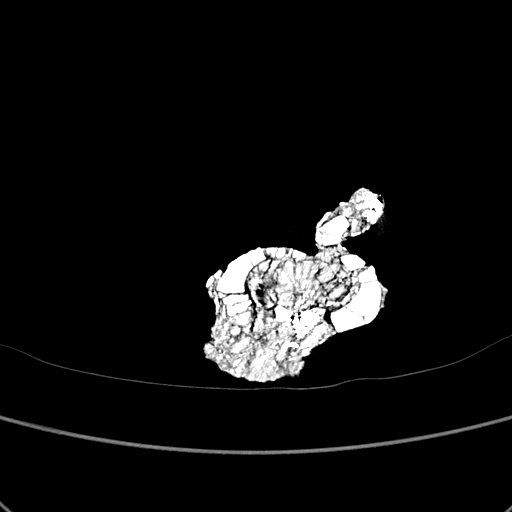

Supplement: S5 File — (ZIP) [file pone.0154403.s006.zip › S2_Files/WWCERATBC.Ser2.Img161.tif]

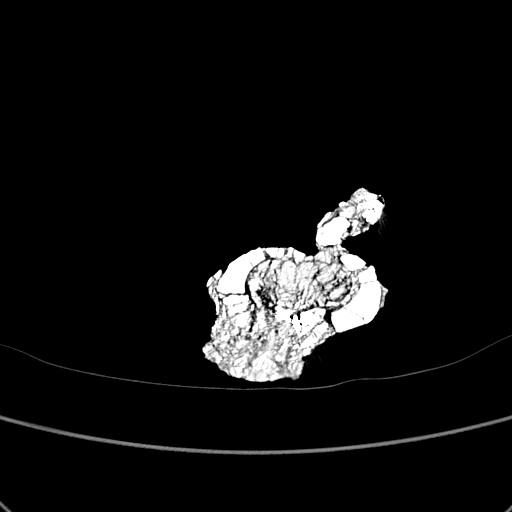

Supplement: S5 File — (ZIP) [file pone.0154403.s006.zip › S2_Files/WWCERATBC.Ser2.Img162.tif]

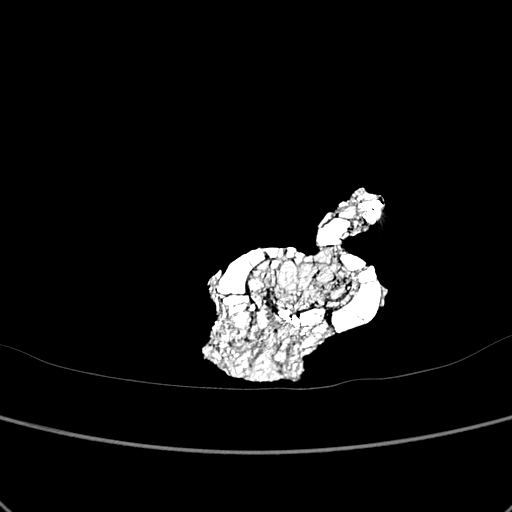

Supplement: S5 File — (ZIP) [file pone.0154403.s006.zip › S2_Files/WWCERATBC.Ser2.Img163.tif]

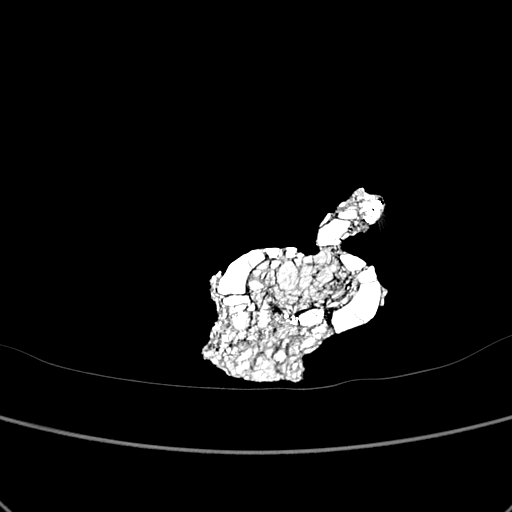

Supplement: S5 File — (ZIP) [file pone.0154403.s006.zip › S2_Files/WWCERATBC.Ser2.Img164.tif]

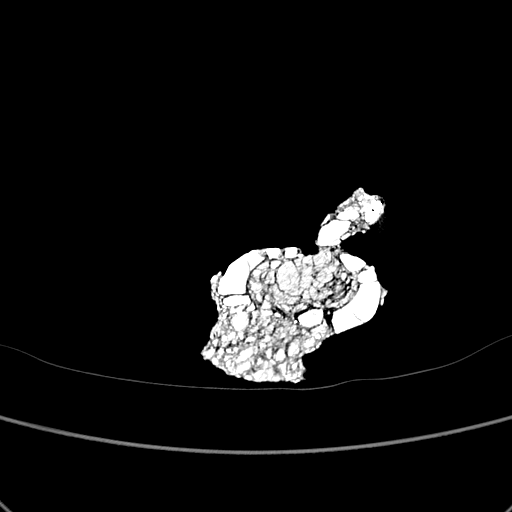

Supplement: S5 File — (ZIP) [file pone.0154403.s006.zip › S2_Files/WWCERATBC.Ser2.Img165.tif]

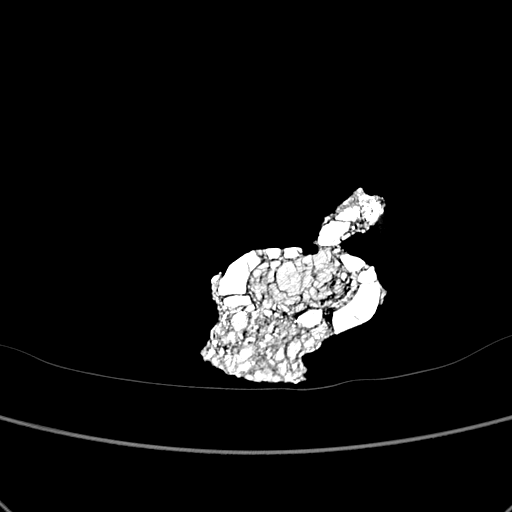

Supplement: S5 File — (ZIP) [file pone.0154403.s006.zip › S2_Files/WWCERATBC.Ser2.Img166.tif]

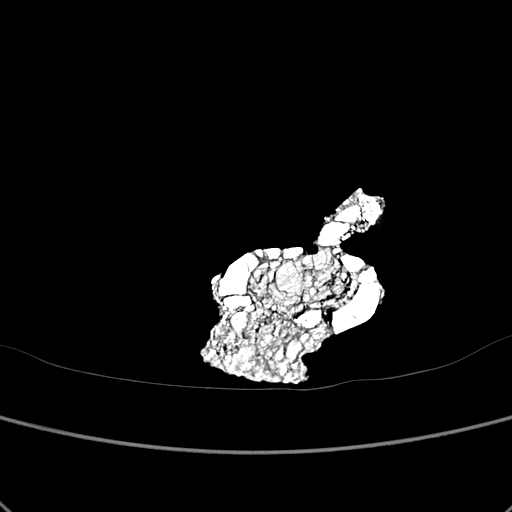

Supplement: S5 File — (ZIP) [file pone.0154403.s006.zip › S2_Files/WWCERATBC.Ser2.Img167.tif]

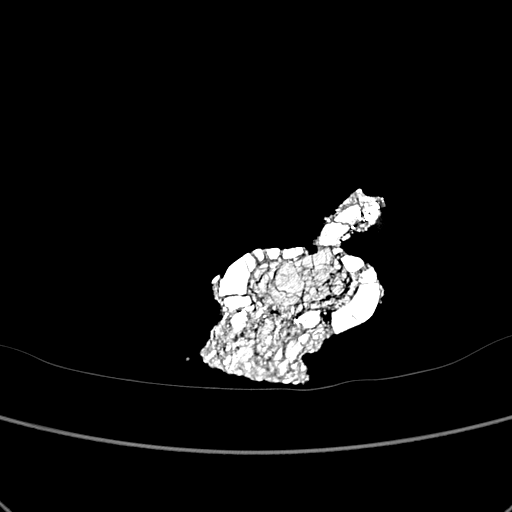

Supplement: S5 File — (ZIP) [file pone.0154403.s006.zip › S2_Files/WWCERATBC.Ser2.Img168.tif]

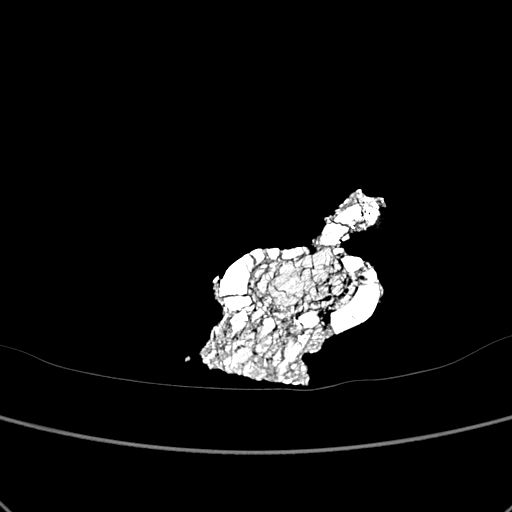

Supplement: S5 File — (ZIP) [file pone.0154403.s006.zip › S2_Files/WWCERATBC.Ser2.Img169.tif]

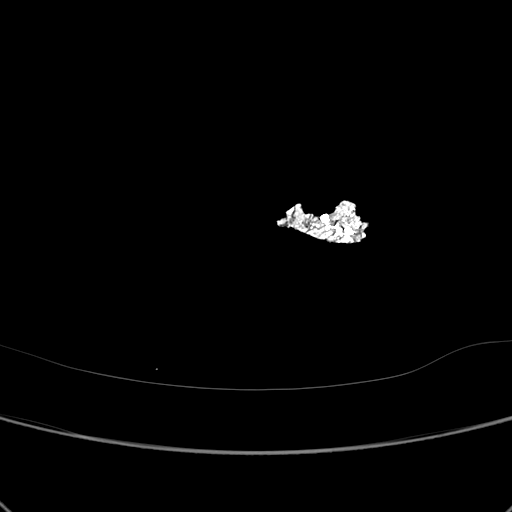

Supplement: S5 File — (ZIP) [file pone.0154403.s006.zip › S2_Files/WWCERATBC.Ser2.Img17.tif]

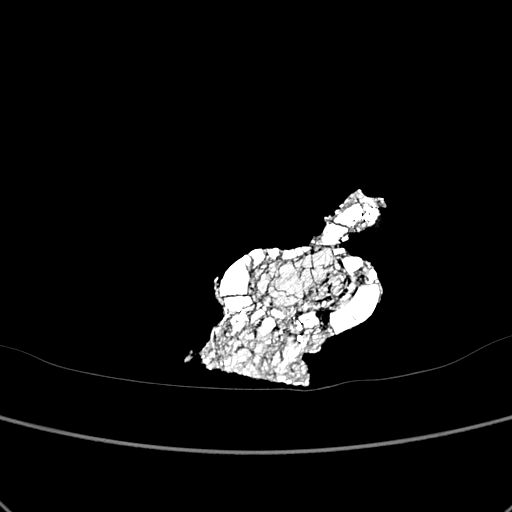

Supplement: S5 File — (ZIP) [file pone.0154403.s006.zip › S2_Files/WWCERATBC.Ser2.Img170.tif]

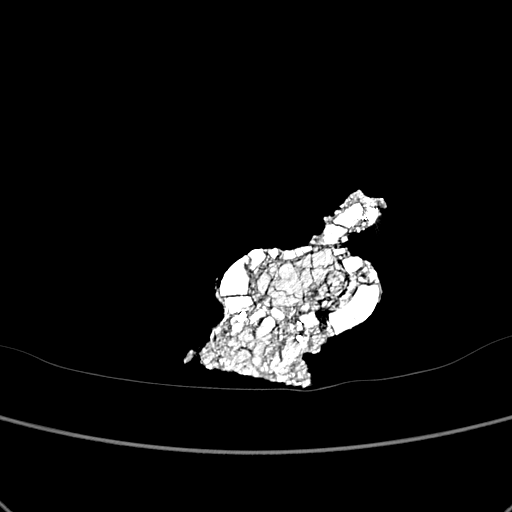

Supplement: S5 File — (ZIP) [file pone.0154403.s006.zip › S2_Files/WWCERATBC.Ser2.Img171.tif]

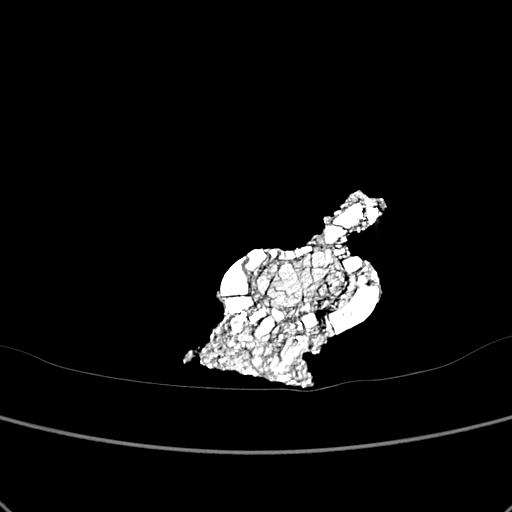

Supplement: S5 File — (ZIP) [file pone.0154403.s006.zip › S2_Files/WWCERATBC.Ser2.Img172.tif]

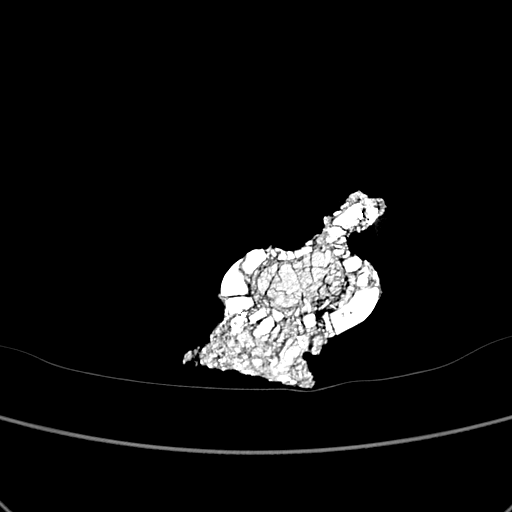

Supplement: S5 File — (ZIP) [file pone.0154403.s006.zip › S2_Files/WWCERATBC.Ser2.Img173.tif]

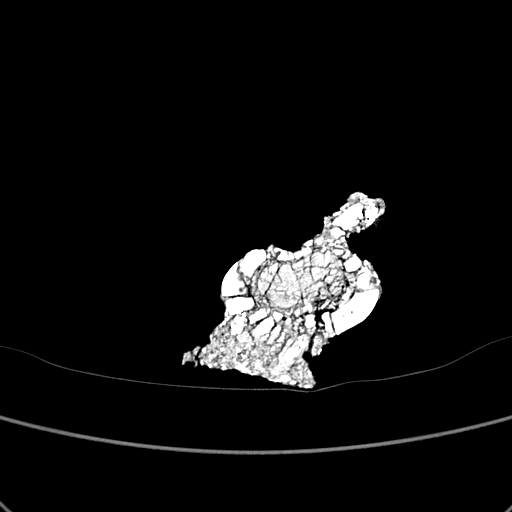

Supplement: S5 File — (ZIP) [file pone.0154403.s006.zip › S2_Files/WWCERATBC.Ser2.Img174.tif]

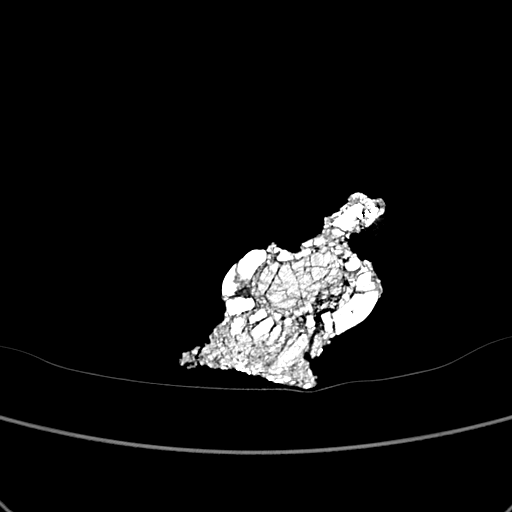

Supplement: S5 File — (ZIP) [file pone.0154403.s006.zip › S2_Files/WWCERATBC.Ser2.Img175.tif]

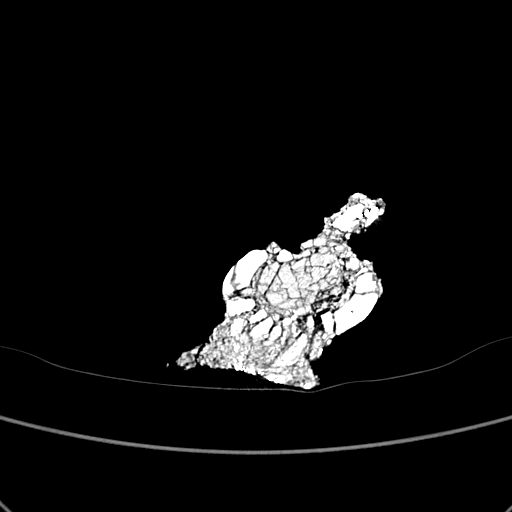

Supplement: S5 File — (ZIP) [file pone.0154403.s006.zip › S2_Files/WWCERATBC.Ser2.Img176.tif]

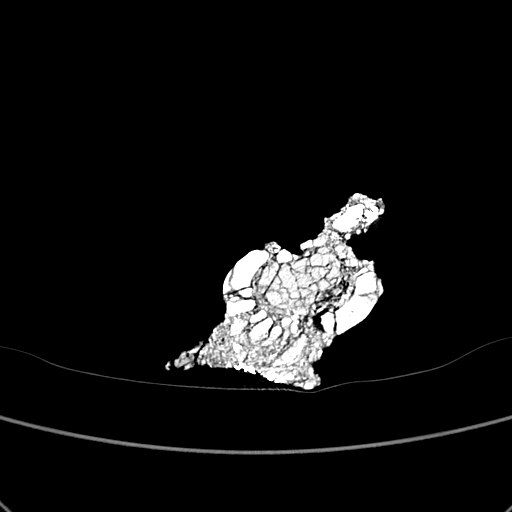

Supplement: S5 File — (ZIP) [file pone.0154403.s006.zip › S2_Files/WWCERATBC.Ser2.Img177.tif]

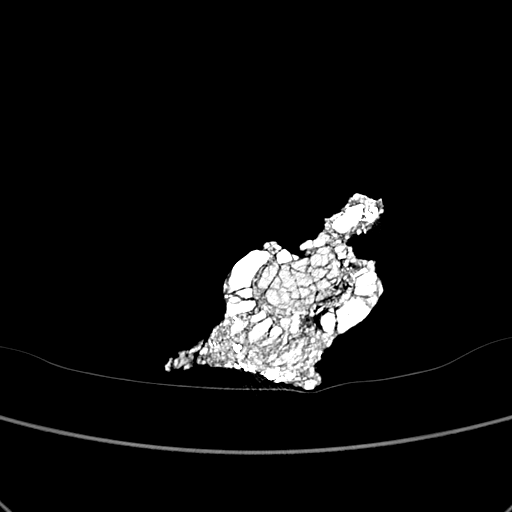

Supplement: S5 File — (ZIP) [file pone.0154403.s006.zip › S2_Files/WWCERATBC.Ser2.Img178.tif]

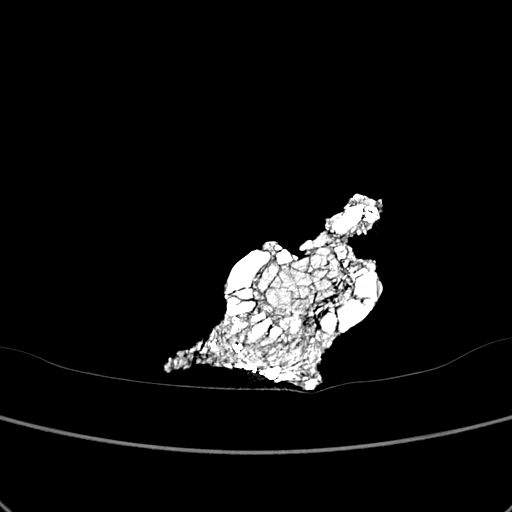

Supplement: S5 File — (ZIP) [file pone.0154403.s006.zip › S2_Files/WWCERATBC.Ser2.Img179.tif]

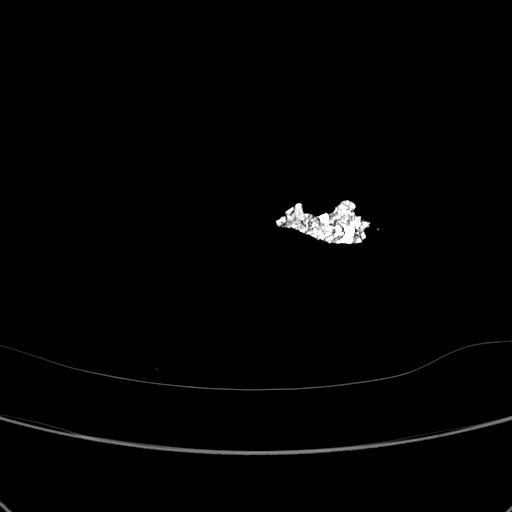

Supplement: S5 File — (ZIP) [file pone.0154403.s006.zip › S2_Files/WWCERATBC.Ser2.Img18.tif]

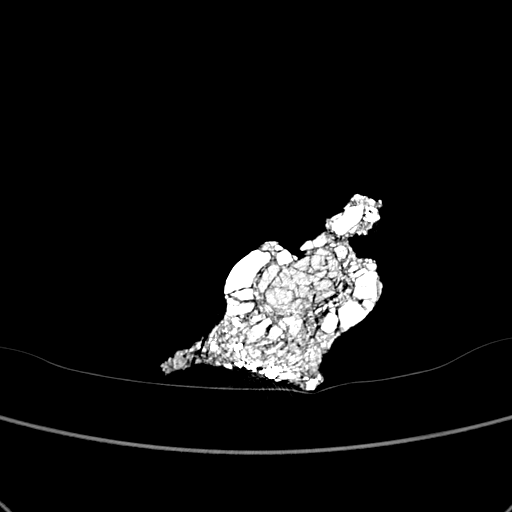

Supplement: S5 File — (ZIP) [file pone.0154403.s006.zip › S2_Files/WWCERATBC.Ser2.Img180.tif]

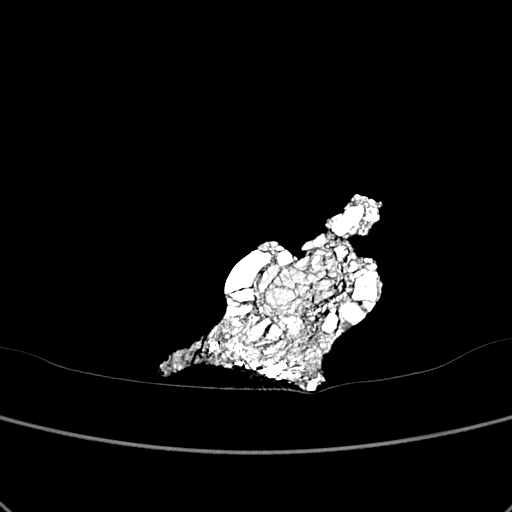

Supplement: S5 File — (ZIP) [file pone.0154403.s006.zip › S2_Files/WWCERATBC.Ser2.Img181.tif]

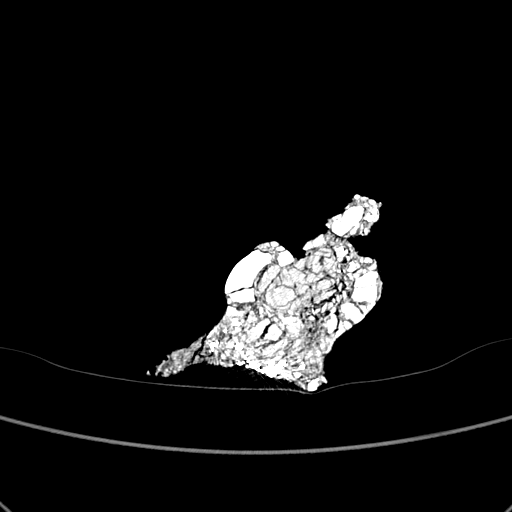

Supplement: S5 File — (ZIP) [file pone.0154403.s006.zip › S2_Files/WWCERATBC.Ser2.Img182.tif]

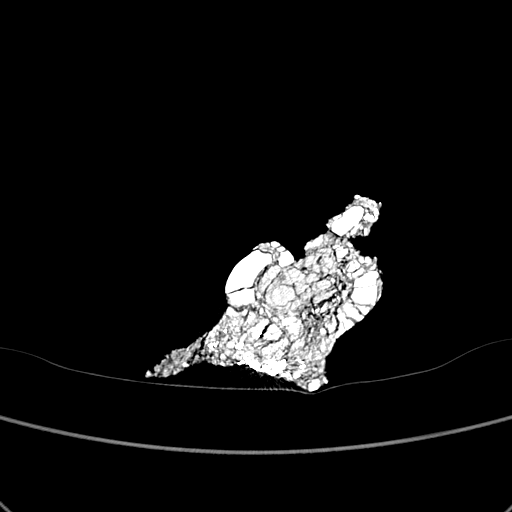

Supplement: S5 File — (ZIP) [file pone.0154403.s006.zip › S2_Files/WWCERATBC.Ser2.Img183.tif]

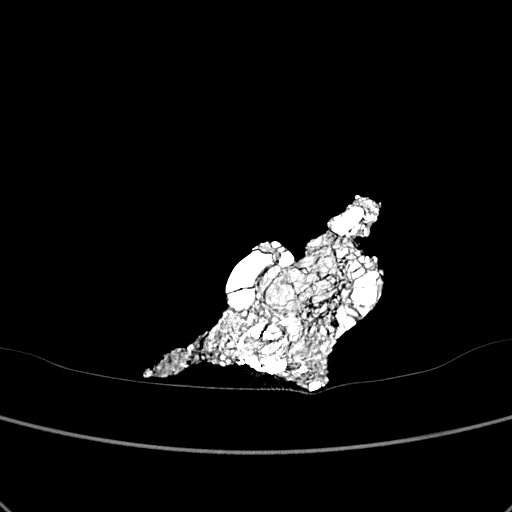

Supplement: S5 File — (ZIP) [file pone.0154403.s006.zip › S2_Files/WWCERATBC.Ser2.Img184.tif]

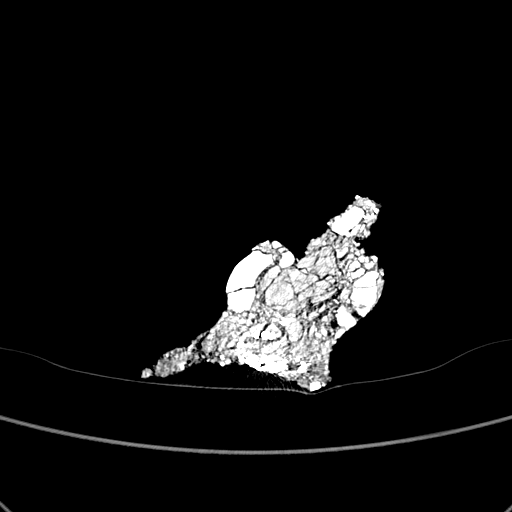

Supplement: S5 File — (ZIP) [file pone.0154403.s006.zip › S2_Files/WWCERATBC.Ser2.Img185.tif]

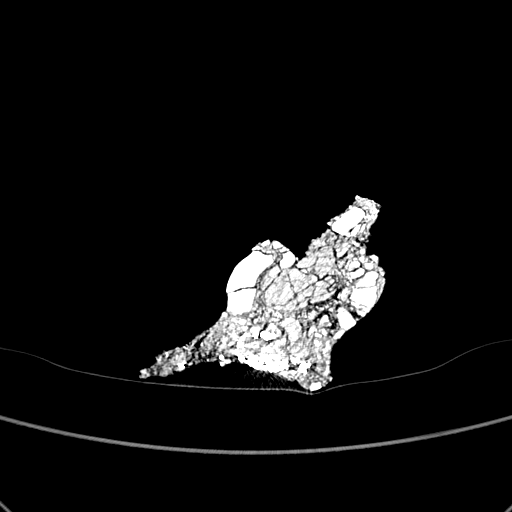

Supplement: S5 File — (ZIP) [file pone.0154403.s006.zip › S2_Files/WWCERATBC.Ser2.Img186.tif]

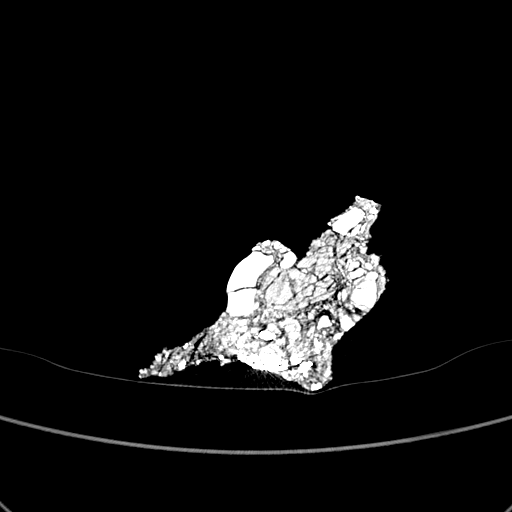

Supplement: S5 File — (ZIP) [file pone.0154403.s006.zip › S2_Files/WWCERATBC.Ser2.Img187.tif]
