# Supplementary material for: A New Centrosaurine Ceratopsid, Machairoceratops cronusi gen et sp. nov., from the Upper Sand Member of the Wahweap Formation (Middle Campanian), Southern Utah
Source: PLoS One. 2016 May 18;11(5):e0154403. doi: 10.1371/journal.pone.0154403 (PMC4871575; doi:10.1371/journal.pone.0154403)
Supplement: S1 File — (DOCX) [file pone.0154403.s002.docx]

APPENDIX A. Specific specimen sources used for character scoring

*Leptoceratops gracilis*  AMNH 2571; CMN 8887, 8889

*Protoceratops andrewsi* AMNH 6251,6408, 6414, 6418, 6425, 6429, 6430, 6438, 6441, 6443, 6444, 6447, 6449,6451, 6466, 6473, 6477, 6480, 6483, 6485, 6486, 6487 6637, 6638; BMNH R6640; R10060; IGM 100-500, 100-502, 100-522, 100-581,

*Magnirostris dodsoni* IVPP V12513

*Bagaceratops rozhdestvenskyi* IGM 100-1008, 100-1013

*Turanoceratops tardabilis* ZIN PH 1868/16

*Zuniceratops christopheri* MSM P2101, 2102, 2110, 2224, 2225, 3196, 3197, 3201 and other MSM specimens

*Chasmosaurus belli* AMNH 5402; CMN 2245; ROM 843; YPM 2016

*Pentaceratops sternbergii* AMNH 1624; MNA 1747; NMMNM P25084, 27468, 50000, OMNH 10165; PMU R200; SMP V1500

*Diabloceratops eatoni* UMNH VP 16699

*Albertaceratops nesmoi* TMP 2002.26.1

*Rubeosaurus ovatus* MOR 492; USNM 11869

*Styracosaurus albertensis* AMNH 5361; CMN 344; ROM 1436; TMP 66.10.28, 89.97.01 2005.42.58

*Spinops sternbergorum* NHMUK R16307, 16308, 16309

*Centrosaurus apertus* AMNH 5239, 5432; NHMUK R4244, 4245, 4859; CMN 348, 971, 8790, 8795, 8798, 11839; ROM 767, 793, 3521, 12782, 12787, 43214; SDNHM 32700, 32702; TMP 67.20.241, 79.11.38, 80.18.115, 82.16.11, 93.36.117, 95.175.64, 95.400.43, UALVP 11735, 16248; USNM 8897; YPM 2015

*Coronosaurus brinkmani* TMP 2002.68.1-3, 5-7, 10-13, 18, 21, 30-32, 38, 41, 43, 46, 56, 75-84, 87, 89-91, 94, 97-101, 105, 111, 114, 120, 125, 127, 129, 130, 134, 142, 168, 173-174, 176, 182, 191, 192, 195, 1995.12.145, 1995.12.69, 1999.82.1

*Xenoceratops foremostensis* CMN 53282, 54950, 54952-54965

*Sinoceratops zhuchengensis* ZCDM V0010, 0012, various other ZCDM specimens

*Einiosaurus procurvicornis* MOR 373, 456, 891, 681, USNM 12745

*Achelousaurus horneri* MOR 485

*Pachyrhinosaurus canadensis* CMN 8867, 9485, 8863, 9602, 10644

*Pachyrhinosaurus lakustai* TMP 83.55.15, 85.55.211, 86.55.47, 86.55.113, 86.55.157, 86.55.193, 239, 86.55.258, 86.55.261, 87.55.137, 87.55.141, 87.55.164, 87.55.210, 87.55.232, 87.55.252, 87.55.258, 88.55.46, 88.55.146, 89.55.125, 89.55.156, 89.55.170, 89.55.256, 89.55.427, 89.55.499, 89.55.547, 89.55.757, 89.55.781, 89.55.1085, 89.55.1144; 89.55.1234, 89.55.1503

*Pachyrhinosaurus perotorum* DMNH 21200, 21201, 22558

*Avaceratops lammersi* ANSP 15800; MOR 692

*Nasutoceratops titusi* UMNH VP 16800, 19466, 19469

*Wendiceratops pinhornensis* TMP 2011.051.0002, 2011.020.0006, 2011.051.0009, 2011.051.0010, 2013.020.0016, 2013.020.0028, 2013.020.0035, 2013.020.0048, 2014.029.0074, 2014.029.0097, 2014.029.0016,
