# Supplementary material for: A New Centrosaurine Ceratopsid, Machairoceratops cronusi gen et sp. nov., from the Upper Sand Member of the Wahweap Formation (Middle Campanian), Southern Utah
Source: PLoS One. 2016 May 18;11(5):e0154403. doi: 10.1371/journal.pone.0154403 (PMC4871575; doi:10.1371/journal.pone.0154403)
Supplement: S2 File — (DOCX) [file pone.0154403.s003.docx]

APPENDIX B. Phylogenetic Analysis Characters

The 101 characters (80 cranial and 21 postcranial) used in the phylogenetic analysis (for both the cladistic parsimony and Bayesian analyses) are enumerated below. This character list is based on Sampson et al. (2013), but incorporates the newly named taxon *Wendiceratops pinhornensis* from Evans and Ryan (2015). Previous uses of characters prior to Farke et al., (2011) are indicated by citations in square brackets. Characters are listed by general anatomical region, with citations identifying original use within a cladistic context. Original uses within a non-cladistic context (e.g., within a taxonomic diagnosis) are not considered. All character scorings are for the adult condition wherever possible. There is one ordered character, number 20.

**Dermal skull roof**

1. Anterior, extent of dorsal and ventral processes: triangular in lateral view, with short dorsal and ventral processes (0); elongate, with deeply concave caudal margin and hypertrophied dorsal and ventral processes (1); ([1], character 1).
2. Premaxillary septum, shape: anteriorly elongate (0); semicircular (1); ([1], character 4).
3. Premaxillary septum, nasal contribution: septum formed by premaxilla only (0); septum formed by premaxilla and nasal (1); ([2], character 5).
4. Premaxilla, narial strut: absent (0); present (1); ([3], character 1).
5. Premaxilla, septal fossa: absent (0); present (1); ([3], character 4).
6. Premaxilla, triangular process: absent (0); present (1); ([4], character 21).
7. Premaxilla, recess along ventral portion of septum: absent (0); present (1); ([1], character 9).
8. Premaxilla, posteroventral expansion of oral margin: absent (0); present (1); ([4], character 6).
9. Premaxilla, ventral extent of posteroventral oral margin: at or above level of alveolar margin of maxilla (0); well below alveolar margin of maxilla (1); ([5], character 12, modified).
10. Posteroventral oral margin, composition of ventral angle: premaxilla and maxilla (0); premaxilla only (1); ([5], character 12, modified).
11. Premaxilla, position of caudal tip of posteroventral process: inserts into an embayment in the nasal (0); intervenes between nasal and maxilla (1); ([6], character 7).
12. Premaxilla, distal end of posteroventral process forked: absent (0); present (1); ([4], character 14).
13. Premaxilla-nasal contact in dorsal view: premaxillae insert between nasal (0); nasals insert between premaxillae (1); ([2], character 20).
14. Accessory antorbital fenestra: present (0); absent (1); ([4], character 15).
15. Accessory antorbital fenestra size: pronounced, penetration of nasal cavity visible in lateral view (0); slight penetration, nasal cavity not visible in lateral view (1); ([2], character 22).
16. External antorbital fossa, size: large, 20% or more length of body of maxilla (0); greatly reduced or absent, less than 10% length of body of maxilla (1); ([4], character 44).
17. Maxillary tooth row, position: ventrally displaced from anterior edentulous portion of maxilla (0); at same level as anterior edentulous portion of maxilla (1); ([2], character 24).
18. Maxilla, maxillary cavity: absent (0); present (1); ([2], character 26).
19. Ectopterygoid/pterygoid complex: covers entire dorsal surface and laps onto lateral surface of caudal ramus of maxilla (0); ectopterygoid vestigial (1); [8].
20. Nasal, ornamentation type in adult (ORDERED): non-pronounced (0); distinct horncore; (2) pachyostotic boss (1); ([4], character 26, 27, 28, 126, modified).
21. Epinasal ossification on nasal: absent (0); present (1); [8].
22. Nasal, narial spine: absent (0); present (1); ([4], character 22).
23. Postorbital, extent of cornual sinuses in base of supraorbital ornamentation: sinus invades frontal and parietal (0); sinus enters postorbital (1); ([4], character 123, modified).
24. Postorbital, type of supraorbital ornamentation in subadult: pointed apex, horncore at least as tall as anteroposteriorly long (0); rounded apex, horncore anteroposteriorly longer than tall (1); ([9], character10, modified).
25. Postorbital, type of supraorbital ornamentation in adult: horncore (0); rugose boss (1); ([9], character 9, modified).
26. Postorbital, position of supraorbital horncore: centered anterodorsal or dorsal to orbit, narrow base with caudal margin of supraorbital horncore extending to or only slightly behind caudal margin of orbit (0); centered posterodorsal to orbit, broad base with caudal margin of supraorbital horncore extending well behind caudal orbit (1); ([10], character 9).
27. Postorbital, orientation of supraorbital horncore base: dorsally directed (0); dorsolaterally directed (1); ([2], character 38).
28. Postorbital, length of supraorbital horncore: short, less than 15% basal skull length (0); present, elongate, greater than 35% basal skull length (1); ([4], character 58, modified).
29. Postorbital, curvature of supraorbital horncore in lateral view: posteriorly recurved (0); anteriorly curved (1); straight (2); ([6], character 2, modified).
30. Postorbital, curvature of supraorbital horncore in anterior view: medially recurved (0); laterally curved (1); straight (2); ([2], character 41).
31. Prefrontal-prefrontal contact: absent (0); present (1); ([4], character 30, modified).
32. Palpebral, shape: rod-like, articulates with prefrontal only at its base and projects across dorsal orbit, ligamentous attachment (0); blocky, fully fused into dorsal orbital margin, sutural articulation with prefrontal and frontal (1); ([4], character 31, modified).
33. Palpebral, antorbital buttress: absent (0); present (1); ([9], character 7, modified).
34. Palpebral, extent of antorbital buttress: present along only anterorodorsal portion of orbit (0); present along entire anterior portion of orbit (1); ([11], modified).
35. Jugal, size and orientation of jugal body: projects strongly posteroventrally, does not extend below the level of the maxillary tooth row (0); projects nearly ventrally, elongated to extend below the level of the maxillary tooth row (1); ([12], character 22).
36. Jugal infratemporal process: absent (0); present, contacts or nearly contacts infratemporal process of squamosal (1); ([4], character 62, modified).
37. Epijugal attachment scar: large blade like triangle with obtuse angle oriented towards quadratojugal (0); scar roughly equilateral in shape (1); ([13], character 113, modified).
38. Frontal fontanelle leading into supracranial cavity complex: absent (0); present (1); ([6], character 3, modified).
39. Frontal fontanelle, shape: transversely narrow, slit-like (0); key-hole shaped, circular or elongate oval (1); ([4], characters 49 and 50, modified).
40. Parietal, anterior extent on dorsum of skull relative to occipital condyle: anterior end of parietal located well in front of occipital condyle (0); anterior end of parietal lies directly over occipital condyle (1); ([2], character 57).
41. Squamosal, shape of expanded blade: sub-rectangular in outline (0); triangular in outline, posteriorly narrowed (1); ([2], character 59).
42. Squamosal, anteromedial lamina forming the posterolateral floor of dorsotemporal fenestra: absent (0); present (1); ([14], character 9).
43. Squamosal-quadrate contact: socket-like cotylus on ventrolateral squamosal for ball-like quadrate head (0); elongate groove on medial surface of squamosal to receive lamina of quadrate (1); ([4], character 64, modified).
44. Squamosal, thickened, rounded swelling along medial margin: absent, lateral surface of squamosal flat to slightly convex (0); present, lateral surface of squamosal slightly concave (1); ([4], character 90).
45. Dorsal squamosal ridge: (0) absent; (1), weakly developed; (2) prominent series of bumps or continuous raised ridge; ([7], character 98).
46. Parietosquamosal contact, shape in lateral view: straight (0); curved, medially concave (1); ([4], character 119).
47. Parietal, concave median embayment on caudal margin: absent (0); present (1); ([2], character 66).
48. Parietal, shape of concave median embayment: shallow, restricted to center of margin (0); shallow, entire transverse bar is a V-shaped embayment (1); ([4], character 83, modified).
49. Parietal, rim on medial margin of dorsotemporal fenestra: absent (0); present, well-defined, laterally projecting rim defines medial margin of fenestra (1); ([4], character 86).
50. Parietal, sharp median crest: absent (0); present (1); ([2], character 75).
51. Parietal, anterocaudal thickness of transverse bar at narrowest point: narrow and straplike, less than 10% total parietal length (0); broad, 20% or more of total parietal length (1); ([3], character 22).
52. Parietal, median bar, transverse width: narrow and straplike, transverse width less than 10% total parietal length (0); relatively wide, transverse width 15% or more of total parietal length (1); ([3], character 23).
53. Parietosquamosal frill, imbrication of marginal undulations: absent (0); present (1); ([1], character 34).

**Epiossifications**

1. Marginal dermal ossifications on parietal and squamosal: absent (0); present (1); ([4], characters 91 and 92, modified).
2. Episquamosal, midlateral, shape: crescentic or ellipsoidal (0); triangular or elongate (1); ([1], character 45, modified).
3. Episquamosals, number per side: three to five (0); six or more (1); [8].
4. Marginal ossification crossing squamosal-parietal contact: absent (0); present (1); ([1], character 43).
5. Epiparietals, number per side: three (0); five or more (1); ([3], character 28).
6. Epiparietal, locus P0: (0) absent; (1) present; ([7], character 99).
7. Epiparietal locus P1: absent (0); present (1); ([9], character 14, modified).
8. Epiparietal, shape of locus P1: (0) low D-shaped process, wider than long; (1) rugose tongue-shaped process, less than twice as long as wide (2) elongate flattened process or spike, greater than twice as long as wide; ([7], character 100).
9. Epiparietal, curvature of locus P1: (0) straight; (1) laterally curved; (2) dorsally curved; ([7], character 101).
10. Epiparietal, shape of locus P2: low D-shaped process, wider than long (0); elongate flattened process or spike, longer than wide (1); ([9], character 15, modified).
11. Epiparietal, curvature of locus P2: straight (0); laterally curved (1); medially curved (2); dorsally curved (3); ([9], character 15, modified).
12. Epiparietal, shape of locus P3: low D-shaped or triangular process (0); elongate flattened process or spike (1); ([9], character 16, modified).
13. Epiparietal, curvature of locus P3: uncurved or slightly medially curved (0); laterally curved (1); dorsally curved (2); [8].
14. Epiparietal, locus P4 shape: low raised D-shaped process (0); elongate spike (1); ([2], character 102, modified).
15. Epiparietal, locus P5: absent (0); present (1); [8].
16. Epiparietal, locus P5 shape: low D-shaped or triangular process (0); elongate spike (1); [8].
17. Epiparietal, locus P6: absent (0); present (1); [8].
18. Epiparietal, locus P6 shape: low D-shaped or triangular process (0); elongate spike (1); [8].
19. Epiparietal, locus P7: absent (0); present (1); [8].

**Braincase**

1. Supraoccipital, contribution to foramen magnum: forms dorsal margin of foramen magnum (0); eliminated from margin by exoccipital-exoccipital contact on midline (1); ([4], character 63).

**Lower jaw**

1. Predentary, dentary processes: ventral processes much longer than abbreviated dorsal processes (0); dorsal and ventral processes elongate and subequal in length (1); ([2], character 114).
2. Predentary, orientation of triturating surface: nearly horizontal (0); inclined steeply laterally (1); ([1], character 57).
3. Dentary lateral ridge confluent with cutting surface of predentary: present (0); absent (1); ([2], character 116).
4. Dentary, caudal extent of tooth row: terminates at the center of the coronoid process (0); terminates caudal to the coronoid process (1); ([15], character 18).

**Dentition**

1. Teeth, number of roots: one (0); two (1); ([4], character 34).
2. Teeth, number of alveoli in dentary: fewer than 20 (0); more than 20 (1); ([5], character 104).
3. Teeth, number of replacements per alveolus: one or two replacement teeth (0); three or more replacement teeth (1); ([13], character 137).

**Axial skeleton**

1. Cervical vertebrae, formation of syncervical: C1-3 fused or tightly articulated, atlantal hypocentrum present as a ventrally placed, wedge-like bone (0); C1-3 firmly fused, atlantal hypocentrum forms a complete ring (1); ([4], character 122).
2. Axis, neural spine shape and orientation: blade-like and nearly vertical, overhangs only anteriormost portion of C3 (0); blade-like morphology lost, spine steeply angled to reach caudal margin of C3 (1); ([13], character 141).
3. Atlantal rib: present (0); absent (1); ([2], character 129).
4. Dorsal vertebrae, shape of centra: relatively axially elongate (0); axially shortened (1); ([2], character 130).
5. Sacrum, longitudinal sulcus on ventral surface: absent (0); present (1); ([13], character 144).

**Pectoral girdle and forelimb**

1. Scapula, relative contribution to glenoid fossa: scapula and coracoid contribute equally (0); scapula contributes well over half of the glenoid (1); ([13], character 145).
2. Olecranon process: relatively small (0); enlarged (>one-third of ulnar length) (1); ([4], character 104, modified).
3. Clavicle: present (0); absent (1); ([13], character 147).
4. Manual and pedal unguals, shape: taper to distal tip (0); dorsoventrally flattened with blunt and rounded distal tips (1); ([15], character 64).
5. Manal and pedal penultimate phalanges shape: length exceeds width (0); width exceeds length (1); ([2], character 137).

**Pelvic girdle and hind limb**

1. Ilium, lateral eversion of dorsal margin: absent (0); present (1); ([4], characters 108-109, modified).
2. Ilium, relative lengths of pubic and ischial peduncles: pubic and ischial peduncles long, extend well below body of ilium approximately the same distance (0); ischial peduncle reduced along ventral aspect, pubic peduncle projects further ventrally than ischial peduncle (1); ([2], character 139).
3. Pubis, prepubic process: short and unexpanded distally (0); elongate, distal end greatly expanded dorsoventrally (1); ([4], character 111).
4. Pubis, position and length of postpubic rod: relatively short but extends past ischial peduncle of ilium, arises ventral to acetabulum and lies along ventral and ventromedial margin of ischium (0); very abbreviated, terminates at level of ischial peduncle, arises medial to acetabulum and passes entirely medial to ischium (1); ([4], character 110).
5. Pubis and ischium, morphology of contributions to acetabulum: pubic acetabular surface faces posterolaterally, pubis and pubic process of ischium contribute equally to ventral margin of acetabulum (0); pubic acetabular surface faces laterally and forms a partial medial wall to the acetabulum, pubic process of ischium elongate and meets pubis close to anterior margin of acetabulum, ventral portion of pubic acetabular surface lies medial to pubic ramus of ischium (1); ([2], character 142).
6. Ischium, cross-sectional shape of shaft: thick and ovoid (0); laterally compressed and bladelike, tapered dorsally (1); ([4], character 112).
7. Ischium, orientation of shaft: nearly straight or slightly decurved (0); broadly and continuously curved (1); ([4], character 113).
8. Femur, morphology of greater and lesser trochanters: trochanters distinct and located below the level of the femoral head (0); trochanters coalesced and level with the femoral head (1); ([1], character 72).
9. Femur, size of fourth trochanter: large and pendant (0); small, reduced to low prominence (1); ([13], character 154).
10. Femur-tibia proportion: tibia longer than femur (0); femur longer than tibia (1); ([4], character 103).
11. Pes, metatarsal proportions: length of MT I two-thirds the length of MT II (0); MT I reduced to one half or less the length of MT II (1); ([2], character 148).

**REFERENCES**

1. Dodson P, Forster CA, Sampson SD. Ceratopsidae. In: Weishampel DB, Dodson P, Osmólska H, editors. The Dinosauria. 2^nd^ ed. Berkeley: University of California Press; 2004. p. 494–513.
2. Sampson SD, Loewen MA, Farke AA, Roberts EM, Forster CA. New Horned Dinosaurs from Utah Provide Evidence for Intracontinental Dinosaur Endemism. PLoS ONE. 2010; 5(9): e12292. doi:10.1371/journal.pone.0012292.
3. Holmes RB, Forster CA, Ryan M, Sheperd KM. A new species of *Chasmosaurus* from the Dinosaur Park Formation of southern Alberta. Canadian Journal of Earth Sciences. 2001; 38:1423–1438.
4. Forster CA. The cranial morphology of *Triceratops*, and a preliminary phylogeny of the Ceratopsia. Ph.D. Dissertation, University of Pennsylvania. 1990. Available: http://repository.upenn.edu/dissertations/AAI9101154
5. Makovicky PJ, Norell MA. *Yamaceratops dorngobiensis*, a new primitive ceratopsian (Dinosauria: Ornithischia) from the Cretaceous of Mongolia. American Museum Novitates. 2006; 3530:1–41.
6. Forster CA, Sereno PC, Evans TW, Rowe T. A complete skull of *Chasmosaurus mariscalensis* (Dinosauria: Ceratopsidae) from the Aguja Formation (Late Campanian) of West Texas. Journal of Vertebrate Paleontology. 1993; 13:161–170.
7. Evans DC, Ryan MJ. Cranial Anatomy of *Wendiceratops pinhornensis* gen. et sp. nov., a centrosaurine ceratopsid (Dinosauria: Ornithischia) from the Oldman Formation (Campanian), Alberta, Canada, and the evolution of ceratopsid nasal ornamentation. PLoS ONE. 2015; 10(7): e0130007. doi:10.1371/journal.pone.0130007.
8. Farke AA, Ryan MJ, Barrett PM, Tanke DH, Braman DR, Loewen MA, Graham MR. A new centrosaurine from the Late Cretaceous of Alberta, Canada, and the evolution of parietal ornamentation in horned dinosaurs. Acta Palaeontologica Polonica. 2011; 56:691–702.
9. Sampson SD. Two new horned dinosaurs from the Upper Cretaceous Two Medicine Formation of Montana; with a phylogenetic analysis of the Centrosaurinae (Ornithischia: Ceratopsidae). Journal of Vertebrate Paleontology. 1995; 15:743–760.
10. Lehman TM. A horned dinosaur from the El Picacho Formation of West Texas, and a review of ceratopsian dinosaurs from the American Southwest. Journal of Paleontology. 1996; 70:494–508.
11. Currie PJ, Langston W, Tanke DH. A new species of *Pachyrhinosaurus* (Dinosauria: Ceratopsidae) from the Upper Cretaceous of Alberta, Canada. In: Currie PJ, Langston W, Tanke DH editors. A New Horned Dinosaur from and Upper Cretaceous Bone Bed in Alberta. Ottawa: NRC Research Press; 2008. p. 1–108.
12. Makovicky PJ. A redescription of the *Montanoceratops cerorhynchus* holotype with a review of referred material. In: Ryan MJ, Chinnery-Allgeier BJ, Eberth DA, editors. New Perspectives on Horned Dinosaurs. Bloomington: Indiana University Press; 2010. p. 68–82.
13. Sereno PC. The evolution of dinosaurs. Science. 1999; 284:2137–2147.
14. Penkalski P, Dodson P. The morphology and systematics of *Avaceratops*, a primitive horned dinosaur from the Judith River Formation (Late Campanian) of Montana, with the description of a second skull. Journal of Vertebrate Paleontology. 1999; 19:692–711.
15. Chinnery BJ, Weishampel DB. *Montanoceratops cerorhynchus* (Dinosauria: Ceratopsia) and relationships among basal neoceratopsians. Journal of Vertebrate Paleontology. 1998; 18:569–585.
