# Supplementary material for: A New Centrosaurine Ceratopsid, Machairoceratops cronusi gen et sp. nov., from the Upper Sand Member of the Wahweap Formation (Middle Campanian), Southern Utah
Source: PLoS One. 2016 May 18;11(5):e0154403. doi: 10.1371/journal.pone.0154403 (PMC4871575; doi:10.1371/journal.pone.0154403)
Supplement: S3 File — (DOCX) [file pone.0154403.s004.docx]

APPENDIX C. Parameter settings in MrBayes.

#NEXUS

BEGIN DATA;

DIMENSIONS NTAX=26 NCHAR=101;

FORMAT DATATYPE=standard symbols="01234567" GAP= - MISSING = ? interleave;

matrix

Leptoceratops_gracilis1--0---0010001-00?0--0-00-----000-0000-00000000-00--------0-------------00000000000000000000000000000

Protoceratops_andrewsi 0--0---0000000100000 00-0?--?--000-0000-0 0100000-00 00-0----0- ------------00000000 000000000000000000000

Magnirostris_dodsoni 1--0---000110000000100-?1000--000-0000-??????0????--------?-??----------00100?0??????????????????????

Bagaceratops_rozhdestvenskyi 1--0---000100000000000-?0-----000-0000-001???00-?0 --------?- ??----------0010000??????????????????????

Turanoceratops_tardabilis ???????????????0000???-?0001-1?1?????0-????????????????????? ?????????????00????0?????????????????????

Zuniceratops_christopheri 1000000???010000000--100000102?1101100-?0?0?????0010-0----?- ??----------00000000???110?????????01?0??

Chasmosaurus_belli 100111000-0100111111101?000(01)0201111111111011111111 0001110001020300-0-0-011011111111111111111111111111

Pentaceratops_sternbergii 100111000-01101111111010001112 01111111111011111111 0001110001020300-0-0-01101111111111111111111111?111

Diabloceratops_eatoni 01100001010010000?0001??000102?110110100 0110101011 11110011011100010101011??????1?????????????????????

Machairoceratops_cronusi ????????????????????????000102????111???0?10101011 11??????0122??????????1????????????????????????????

Wendiceratops_pinhornensis ?????????????1?101?10???????????????????01102010?1 11110011011212110100?0?????11????1?10?1?11???11????

Albertaceratops_nesmoi ?1?000??????01-101?00???000112?111111??00110101011 11110011012110000101 01?1101111 ?????????????????????

Rubeosaurus_ovatus 01??0001?1??0??????1??0?000022?1??????????????101??1?100?100 ??00101111?1?????????????????????????????

Styracosaurus_albertensis 01100001111001-11111010000002211111111000110101011 1111001101220001111111111011111?111101111111101111?

Spinops_sternbergorum ??1?00???? ???1-????1??0?000022?111???10? ????1?101111?100??01 2212000?0?0??????????????????????????????

Centrosaurus_apertus 01100001111001-111110100000020111111110001101010111111001101 22120001010111101111111111011111111011111

Coronosaurus_brinkmani 011?000111??01-11111010000102011111111000110101011 1111001101121??0010101111011111?11?1????111110?111?

Xenoceratops_foremostensis ????????????????????0???????????????????01?0?010?1 11?1??1101120100010101?????????????????????????????

Sinoceratops_zhuchengensis??1??????????001???1?????00022010-???1000110100-11 1111001111121(13)00010101?????????????1???? 11?????11??

Einiosaurus_procurvicornis ?11?000??????1-111110111100022010-1111000110101011 1111001100??001(01)010101111011111?11?1??1? 111??1111??

Achelousaurus_horneri 011?00011110?1-111120111100022?10-1?11000110101011 111100?100??001101010111101111?????????????????????

Pachyrhinosaurus_canadensis 011000011110?1-11112011?110022?10-111100?11010??11 ???1???100??121101010111101????????????????????????

Pachyrhinosaurus_lakustai 01100001111001-111120111110022?10-1?11000110101011 1111001101??1211010101111011111?11?1??11111110111??

Pachyrhinosaurus_perotorum 01?0000111???1-11?1201??110022?10-???1????1??010?1 11?1???101121(23)11010101?????1?1 ???1???????????0111??

Avaceratops_lammersi 01?000011110?1-10100011?001111?11011?100 0110200-11--0100?111 0000000101011??011111111?10?11 ???????1?1?

Nasutoceratops_titusi 01100001111001-10100011?001111?110??11000110200-11 1101001101 120000010101???????111?1?10??????????????

;

end;

begin mrbayes;

set autoclose=yes nowarn=yes;

LOG START filename=Machairo_uniform.screenlog;

CTYPE ordered: 20;

LSET coding=variable rates=gamma;

OUTGROUP Leptoceratops_gracilis;

constraint root = 1-. ;

constraint Neoceratopsia = 2-. ; [ingroup]

constraint Ceratopsidae = 7-. ;

constraint Centrosaurinae = 9-. ;

CALIBRATE Leptoceratops_gracilis=uniform(65,67); [1]

CALIBRATE Protoceratops_andrewsi=uniform(70.5,76.5); [2–5]

CALIBRATE Magnirostris_dodsoni =uniform(70.5,76.5); [2–5]

CALIBRATE Bagaceratops_rozhdestvenskyi=uniform(70.5,76.5); [2–5]

CALIBRATE Turanoceratops_tardabilis=uniform(90,91); [6]

CALIBRATE Zuniceratops_christopheri=uniform(90,91); [7]

CALIBRATE Chasmosaurus_belli=uniform(75.5,75.8); [8–10]

CALIBRATE Pentaceratops_sternbergii=uniform(73.7,74.2); [9–11]

CALIBRATE Diabloceratops_eatoni=fixed(79.9); [10,12]

CALIBRATE Albertaceratops_nesmoi=uniform(77.5,78); [9–10, 13]

CALIBRATE Rubeosaurus_ovatus=uniform(74,74.5); [9–10, 14]

CALIBRATE Styracosaurus_albertensis=uniform(75.5,75.8); [8–10]

CALIBRATE Spinops_sternbergorum=uniform(77,79); [9–10, 15]

CALIBRATE Centrosaurus_apertus =uniform(75.5,75.8); [8–10]

CALIBRATE Coronosaurus_brinkmani=uniform(77,77.5); [9–10, 16]

CALIBRATE Xenoceratops_foremostensis=uniform(79,79.5); [9–10, 17]

CALIBRATE Sinoceratops_zhuchengensis =uniform(73.5,76); [18]

CALIBRATE Einiosaurus_procurvicornis=uniform(74,74.5); [9–10, 19]

CALIBRATE Achelousaurus_horneri=uniform(74,74.5); [9–10, 19]

CALIBRATE Pachyrhinosaurus_canadensis=uniform(68.3,72); [1, 20]

CALIBRATE Pachyrhinosaurus_lakustai=fixed(73.27); [21]

CALIBRATE Pachyrhinosaurus_perotorum =uniform(69,70); [22]

CALIBRATE Avaceratops_lammersi=fixed(78.7); [9–10, 23–24]

CALIBRATE Nasutoceratops_titusi =uniform(75.51,75.97); [9–10, 25]

CALIBRATE Wendiceratops_pinhornensis=uniform(78, 79); [26]

CALIBRATE Machairoceratops_cronusi=fixed(77.3); [10, 12]

PRSET brlenspr=clock:uniform;

PRSET clockvarpr=igr;

PRSET igrvarpr=exp(10); [default]

PRSET clockratepr=normal(0.01,0.1);

CALIBRATE root=offsetexponential(65,92);

PRSET topologypr = constraints(root, Neoceratopsia, Ceratopsidae, Centrosaurinae);

PRSET nodeagepr = calibrated;

mcmcp temp=0.2 samplefreq=1000 printfr=1000 nruns=4 nchain=4 relburnin=yes burninfrac=0.25 ;

mcmcp savebrlens=yes filename=Machairo_uniform;

mcmc ngen=20000000;

sumt filename=Machairo_uniform relburnin=yes burninfrac=0.25 contype=allcompat;

sump filename=Machairo_uniform relburnin=yes burninfrac=0.25;

end;

**REFERENCES**

1. Eberth DA, Braman DR. A revised stratigraphy and depositional history for the Horeshoe Canyon Formation (Upper Cretaceous), southern Alberta plains. Can J Earth Sci. 2012; 49:1053–1068.
2. Carpenter K, Hayashi S, Kobayashi Y, Maryańska T, Barsbold R, Sato K, Obata I. *Saichania chulsanensis* (Ornithischia, Ankylosauridae) from the Upper Cretaceous of Mongolia. Palaeontographica Abt. A. 2011; 294:1–61.
3. Shuvalov VF. The Cretaceous stratigraphy and palaeobiogeography of Mongolia. In: Benton MJ, Shishkin MA, Unwin DM, Kurochkin EN, editors. The age of dinosaurs in Russia and Mongolia. Cambridge: Cambridge University Press; 2000. pp. 256–278.
4. Hicks JF, Fastovsku D, Nichols DJ, Watabe M. Magnetostratigraphic correlation of Late Cretaceous dinosaur-bearing localities in the Nemegt and Ulan Nuur Basins, Gobi Desert, Mongolia. Geol Soc of Am Prog Abstr. 2001; 323A.
5. Dashzeveg D, Dingus L, Loope DB, Swisher CC, Dulam T, Sweeney MR. New stratigraphic subdivision, depositional environment, and age estimate for the Upper Cretaceous Djadokhta Formation, southern Ulan Nuur Basin, Mongolia. Am Mus Nov. 2005; 3498:1–31.
6. Sues HD, Averianov A. *Turanoceratops tardabilis*—the first ceratopsid dinosaur from Asia. Naturwiss. 2009; 96:645–652.
7. Wolfe DG, Kirkland JI, Smith D, Poole K, Chinnery-Allgeier B, McDonald A. *Zuniceratops christopheri*: The North American ceratopsid sister taxon reconstructed on the basis of new data. In: Ryan MJ, Chinnery-Allgeier BJ, Eberth DA, editors. New Perspectives on Horned Dinosaurs. Bloomington: Indiana University Press; 2010. pp. 91–98.
8. Ryan MJ, Evans DC. Ornithischian dinosaurs. In: Currie PJ, Koppelhus E, eds. Dinosaur Provincial Park: A spectacular ancient ecosystem reveled. Bloomington: Indiana University Press; 2005. pp. 312–348.
9. Roberts EM, Deino AL, Chan MA. ^40^Ar/^39^Ar age of the Kaiparowits Formation, southern Utah and correlation of contemporaneous Campanian strata and vertebrate faunas along the margin of the Western Interior Basin. Cretaceous Res. 2005; 26:307–318.
10. Roberts EM, Sampson SD, Deino AL, Bowring SI. The Kaiparowits Formation: a remarkable record of Late Cretaceous terrestrial environments, ecosystems and evolution in western North America. In: Titus AL, Loewen MA, editors. At the top of Grand Staircase: the Late Cretaceous of Southern Utah. Bloomington: Indiana University Press. 2013. pp. 85–106.
11. Sullivan RM, Lucas SG. The Kirtlandian land-vertebrate “age” ­– faunal composition, temporal position and biostratigraphic correlation in the nonmarine Upper Cretaceous of western North America. New Mexico Mus Nat Hist Sci Bull. 2006; 35:7–29.
12. Jinnah ZA, Roberts EM, Deino AL, Larsen JS, Link PK, Fanning CM. New ^40^Ar-^39^Ar and detrital zircon U-Pb ages for the Upper Cretaceous Wahweap and Kaiparowits formations on the Kaiparowits Plateau, Utah: implications for regional correlation, provenance, and biostratigraphy. Cretaceous Res. 2009; 30:287–299.
13. Ryan MJ. A new basal centrosaurine ceratopsid from the Oldman Formation, southeastern Alberta. J Paleontol. 2007; 81:376–396.
14. McDonald AT, Horner JR. New material of *Styracosaurus ovatus* from the Two Medicine Formation of Montana. In: Ryan MJ, Chinnery-Allgeier BJ, Eberth DA, editors. New Perspectives on Horned Dinosaurs. Bloomington: Indiana University Press; 2010. p. 156–168.
15. Farke AA, Ryan MJ, Barrett PM, Tanke DH, Braman DR, Loewen MA, Graham MR. A new centrosaurine from the Late Cretaceous of Alberta, Canada, and the evolution of parietal ornamentation in horned dinosaurs. Acta Palaeontol Pol. 2011; 56:691–702.
16. Ryan MJ, Russell AP. A new centrosaurine ceratopsid from the Oldman Formation of Alberta and its implications for centrosaurine taxonomy and systematics. Can J Earth Sci. 2005; 42:1369–1387. doi: 10.1139/e05-029
17. Ryan MJ, Evans DC, Shepard KM. A new ceratopsid from the Foremost Formation (middle Campanian) of Alberta. Can J Earth Sci. 2012; 49:1251–1262.
18. Hone DW, Wang K, Sullivan C, Shau X, Chen S, Li D, Ji S, Ji Q, Xu X. A new, large tyrannosaurine theropod from the Upper Cretaceous of China. Cretaceous Res. 2011; 32:495–505.
19. Sampson SD. Two new horned dinosaurs from the Upper Cretaceous Two Medicine Formation of Montana; with a phylogenetic analysis of the Centrosaurinae (Ornithischia: Ceratopsidae). J Vertebr Paleontol. 1995; 15:743–760.
20. Campbell JA, Ryan MJ, Currie PJ, Langston W Jr. New reconstruction of the parietal morphology of *Pachyrhinosaurus canadensis*, a centrosaurine ceratopsid from the Campanian of Alberta. J VertebrPaleontol Prog Abstr 2012: 74A.
21. Currie PJ, Langston W, Tanke DH. A new species of *Pachyrhinosaurus* (Dinosauria: Ceratopsidae) from the Upper Cretaceous of Alberta, Canada. In: Currie PJ, Langston W, Tanke DH editors. A New Horned Dinosaur from and Upper Cretaceous Bone Bed in Alberta. Ottawa: NRC Research Press; 2008. pp. 1–108.
22. Fiorillo AR, Tykoski RS. A new Maastrichtian species of the centrosaurine ceratopsid *Pachyrhinosaurus* from the North Slope of Alaska. Acta Palaeontol Pol. 2012; 57:561–573.
23. Dodson P. *Avaceratops lammersi*: a new ceratopsid from the Judith River Formation of Montana. Proc Acad Natl Sci. 1986; 138:305-317.
24. Penkalski P, Dodson P. The morphology and systematics of *Avaceratops*, a primitive horned dinosaur from the Judith River Formation (Late Campanian) of Montana, with the description of a second skull. J Vertebr Paleontol. 1999; 19:692–711.
25. Sampson SD, Lund EK, Loewen MA, Farke AA, Clayton KE. A remarkable short-snouted horned dinosaur from the Late Cretaceous (late Campanian) of southern Laramidia. Proc R Soc B. 2013; 280: doi: 10.1098/rspb.2013. 1186.
26. Evans DC, Ryan MJ. Cranial Anatomy of *Wendiceratops pinhornensis* gen. et sp. nov., a centrosaurine ceratopsid (Dinosauria: Ornithischia) from the Oldman Formation (Campanian), Alberta, Canada, and the evolution of ceratopsid nasal ornamentation. PLoS ONE. 2015; 10(7): e0130007. doi:10.1371/journal.pone.0130007.
