# Supplementary figures and images for: A New Centrosaurine Ceratopsid, Machairoceratops cronusi gen et sp. nov., from the Upper Sand Member of the Wahweap Formation (Middle Campanian), Southern Utah (part 3 of 3)
Source: PLoS One. 2016 May 18;11(5):e0154403. doi: 10.1371/journal.pone.0154403 (PMC4871575; doi:10.1371/journal.pone.0154403)

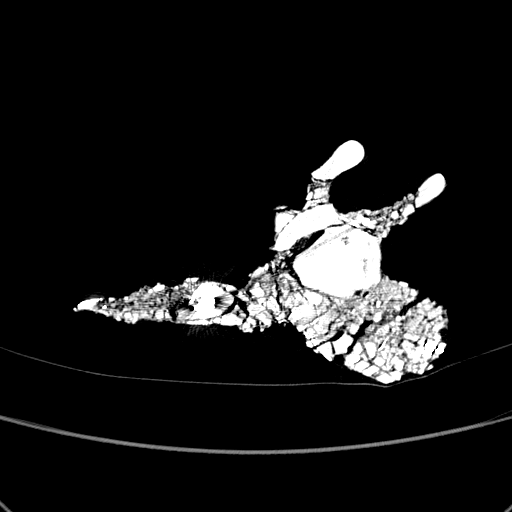

Supplement: S5 File — (ZIP) [file pone.0154403.s006.zip › S2_Files/WWCERATBC.Ser2.Img278.tif]

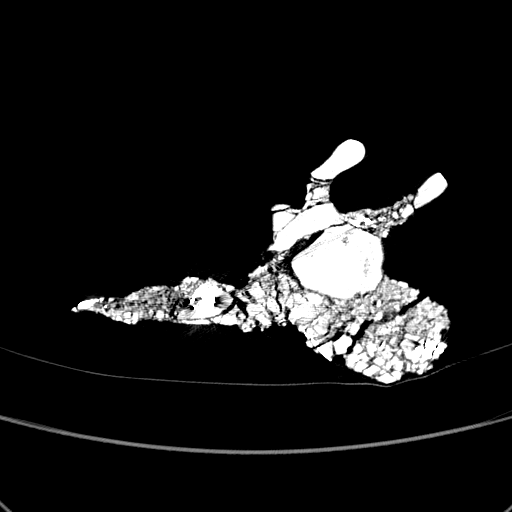

Supplement: S5 File — (ZIP) [file pone.0154403.s006.zip › S2_Files/WWCERATBC.Ser2.Img279.tif]
