# Supplementary figures and images for: A New Centrosaurine Ceratopsid, Machairoceratops cronusi gen et sp. nov., from the Upper Sand Member of the Wahweap Formation (Middle Campanian), Southern Utah (part 2 of 3)
Source: PLoS One. 2016 May 18;11(5):e0154403. doi: 10.1371/journal.pone.0154403 (PMC4871575; doi:10.1371/journal.pone.0154403)

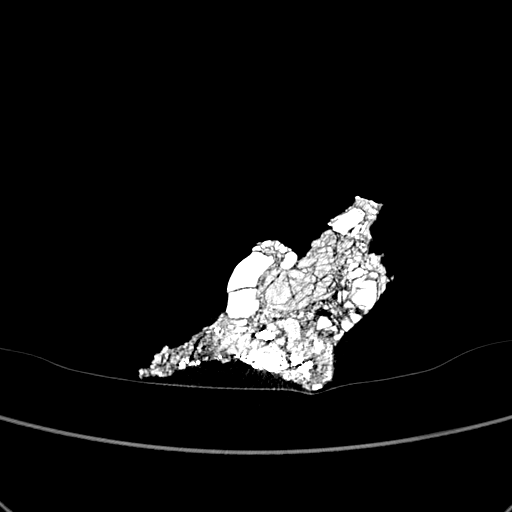

Supplement: S5 File — (ZIP) [file pone.0154403.s006.zip › S2_Files/WWCERATBC.Ser2.Img188.tif]

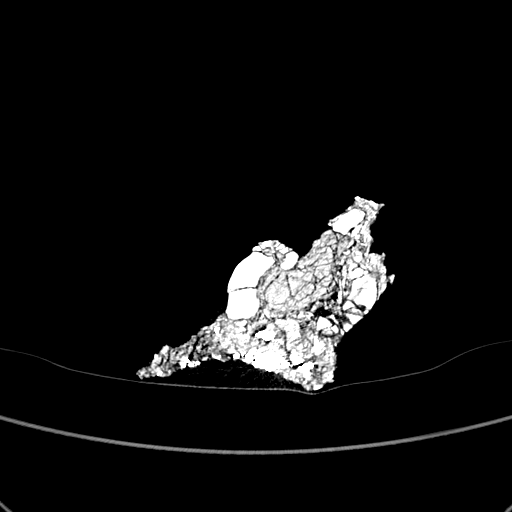

Supplement: S5 File — (ZIP) [file pone.0154403.s006.zip › S2_Files/WWCERATBC.Ser2.Img189.tif]

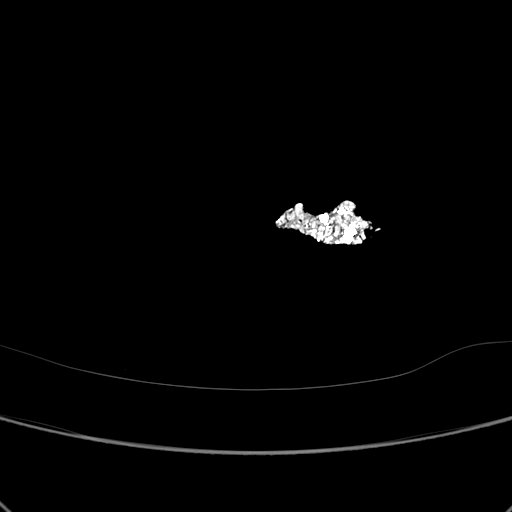

Supplement: S5 File — (ZIP) [file pone.0154403.s006.zip › S2_Files/WWCERATBC.Ser2.Img19.tif]

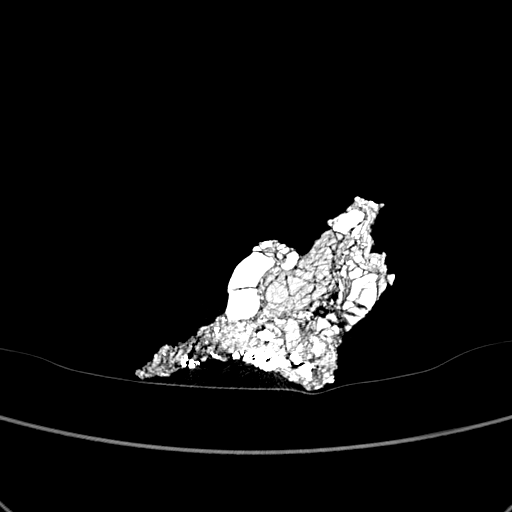

Supplement: S5 File — (ZIP) [file pone.0154403.s006.zip › S2_Files/WWCERATBC.Ser2.Img190.tif]

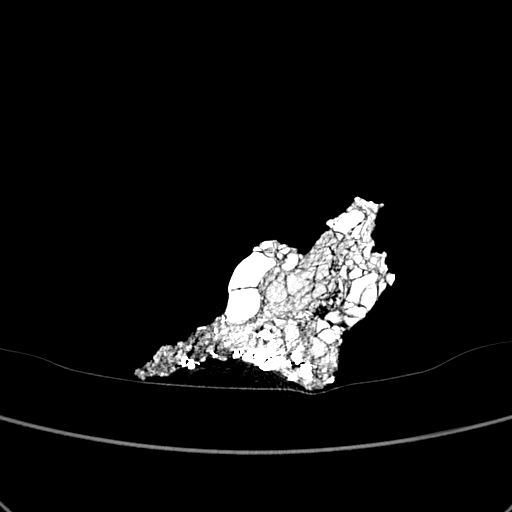

Supplement: S5 File — (ZIP) [file pone.0154403.s006.zip › S2_Files/WWCERATBC.Ser2.Img191.tif]

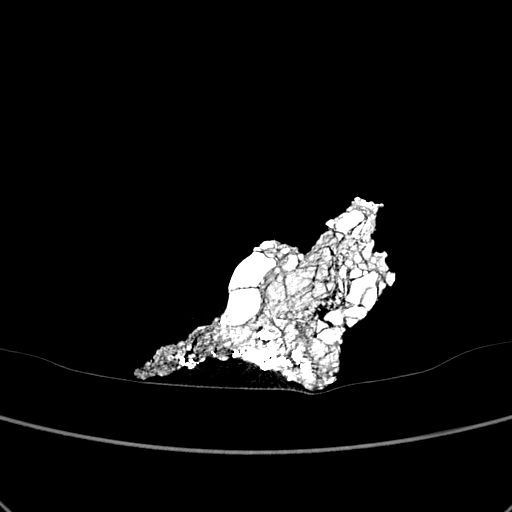

Supplement: S5 File — (ZIP) [file pone.0154403.s006.zip › S2_Files/WWCERATBC.Ser2.Img192.tif]

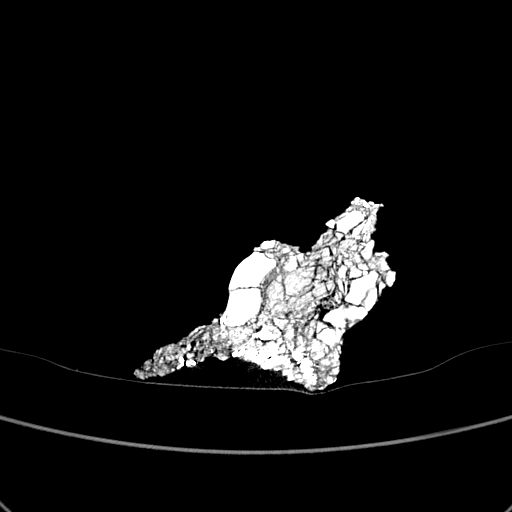

Supplement: S5 File — (ZIP) [file pone.0154403.s006.zip › S2_Files/WWCERATBC.Ser2.Img193.tif]

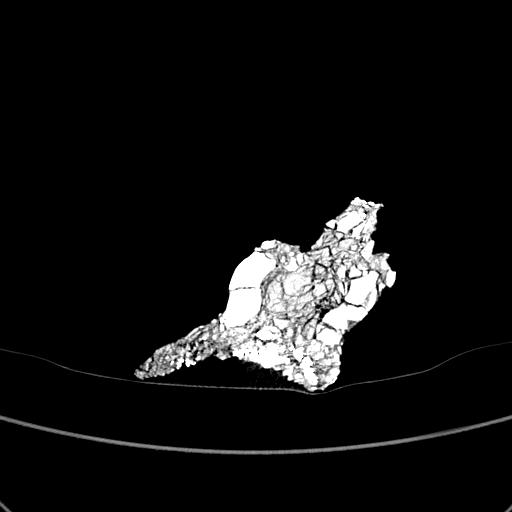

Supplement: S5 File — (ZIP) [file pone.0154403.s006.zip › S2_Files/WWCERATBC.Ser2.Img194.tif]

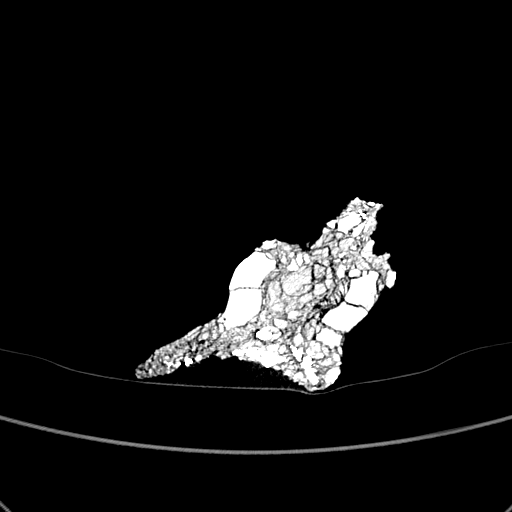

Supplement: S5 File — (ZIP) [file pone.0154403.s006.zip › S2_Files/WWCERATBC.Ser2.Img195.tif]

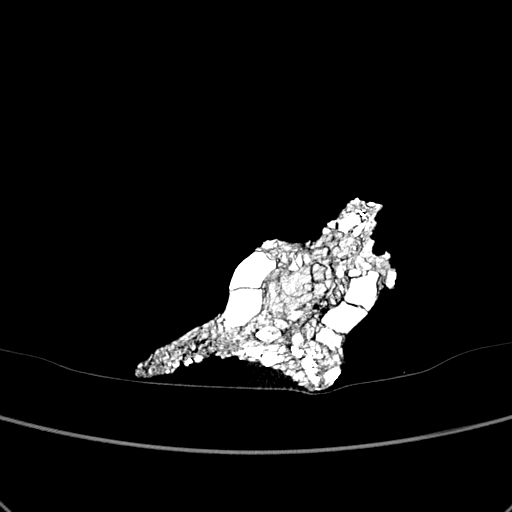

Supplement: S5 File — (ZIP) [file pone.0154403.s006.zip › S2_Files/WWCERATBC.Ser2.Img196.tif]

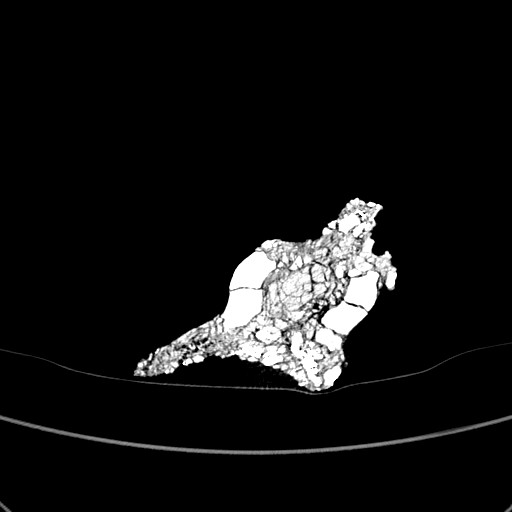

Supplement: S5 File — (ZIP) [file pone.0154403.s006.zip › S2_Files/WWCERATBC.Ser2.Img197.tif]

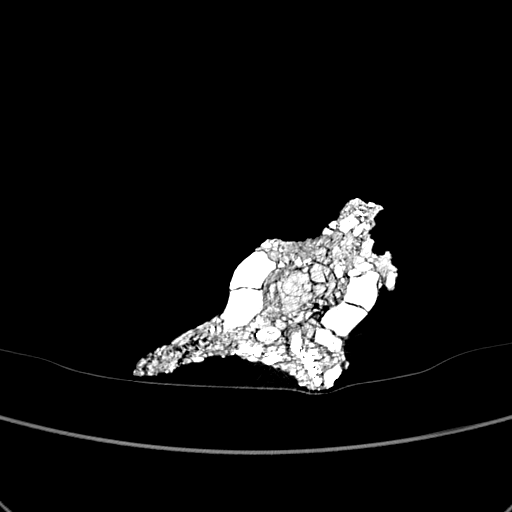

Supplement: S5 File — (ZIP) [file pone.0154403.s006.zip › S2_Files/WWCERATBC.Ser2.Img198.tif]

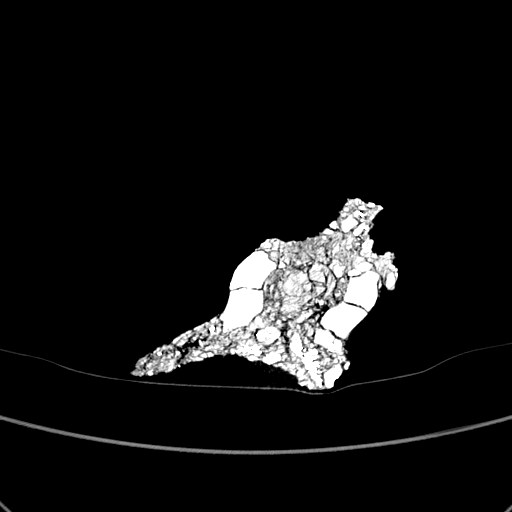

Supplement: S5 File — (ZIP) [file pone.0154403.s006.zip › S2_Files/WWCERATBC.Ser2.Img199.tif]

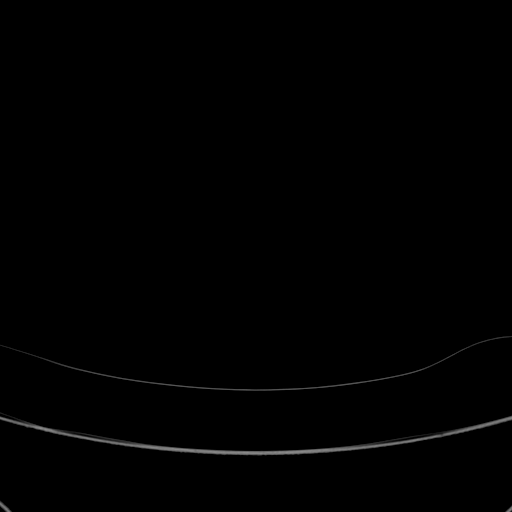

Supplement: S5 File — (ZIP) [file pone.0154403.s006.zip › S2_Files/WWCERATBC.Ser2.Img2.tif]

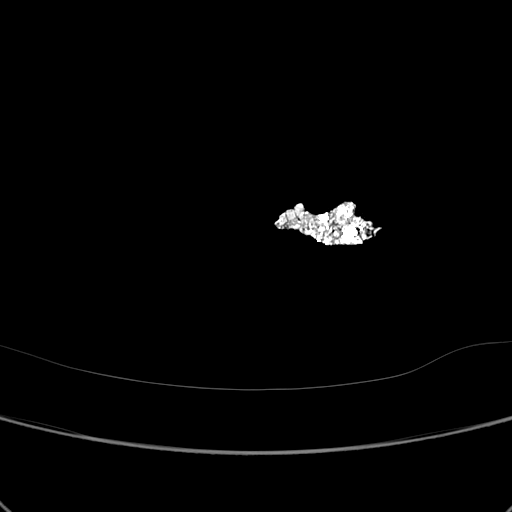

Supplement: S5 File — (ZIP) [file pone.0154403.s006.zip › S2_Files/WWCERATBC.Ser2.Img20.tif]

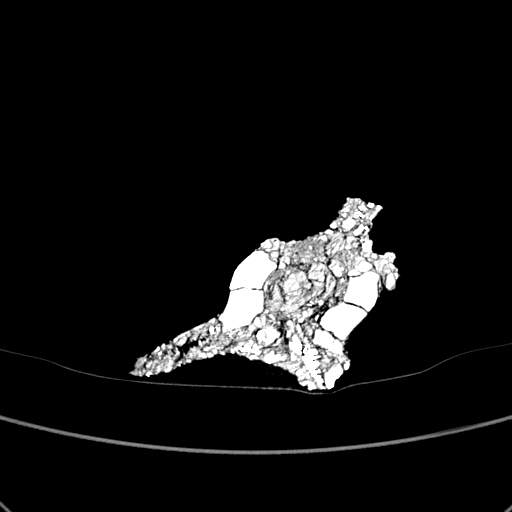

Supplement: S5 File — (ZIP) [file pone.0154403.s006.zip › S2_Files/WWCERATBC.Ser2.Img200.tif]

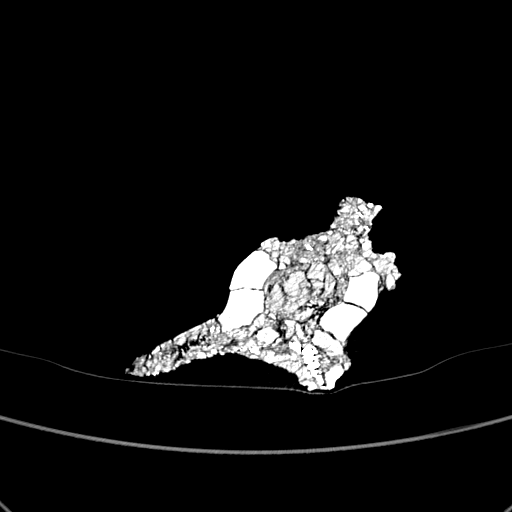

Supplement: S5 File — (ZIP) [file pone.0154403.s006.zip › S2_Files/WWCERATBC.Ser2.Img201.tif]

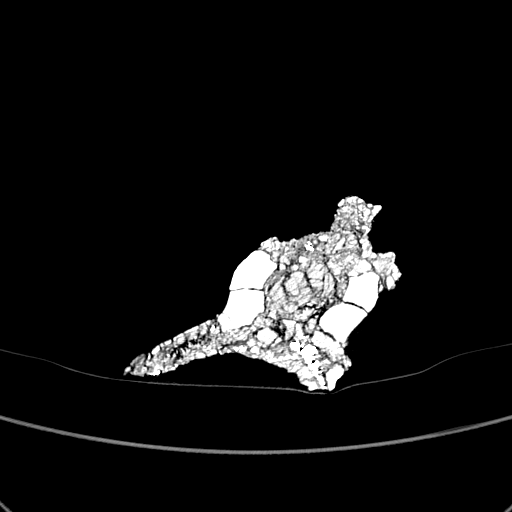

Supplement: S5 File — (ZIP) [file pone.0154403.s006.zip › S2_Files/WWCERATBC.Ser2.Img202.tif]

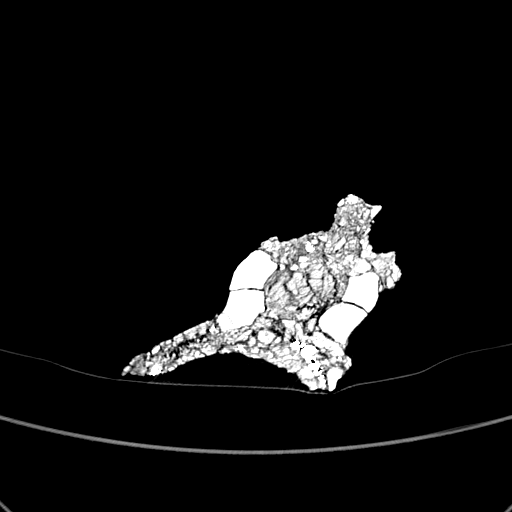

Supplement: S5 File — (ZIP) [file pone.0154403.s006.zip › S2_Files/WWCERATBC.Ser2.Img203.tif]

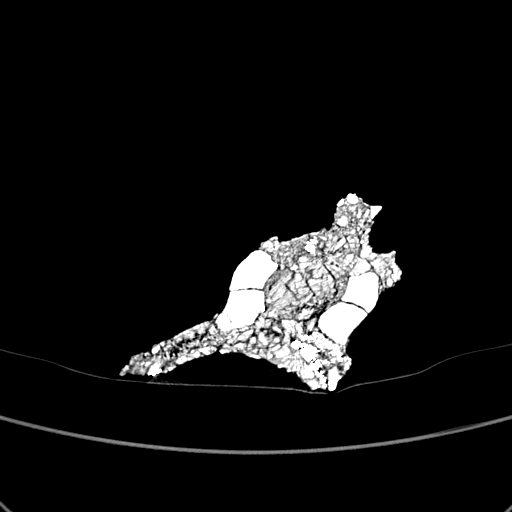

Supplement: S5 File — (ZIP) [file pone.0154403.s006.zip › S2_Files/WWCERATBC.Ser2.Img204.tif]

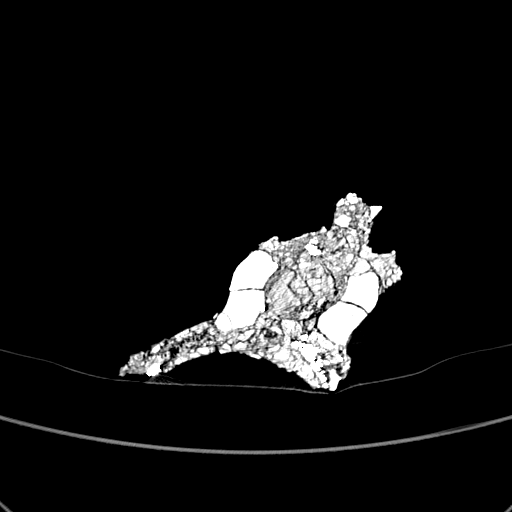

Supplement: S5 File — (ZIP) [file pone.0154403.s006.zip › S2_Files/WWCERATBC.Ser2.Img205.tif]

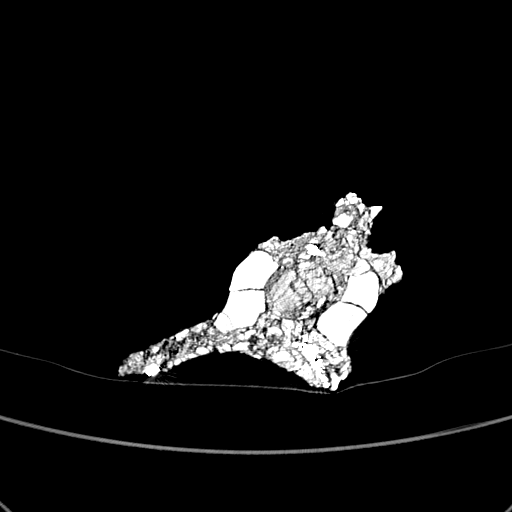

Supplement: S5 File — (ZIP) [file pone.0154403.s006.zip › S2_Files/WWCERATBC.Ser2.Img206.tif]

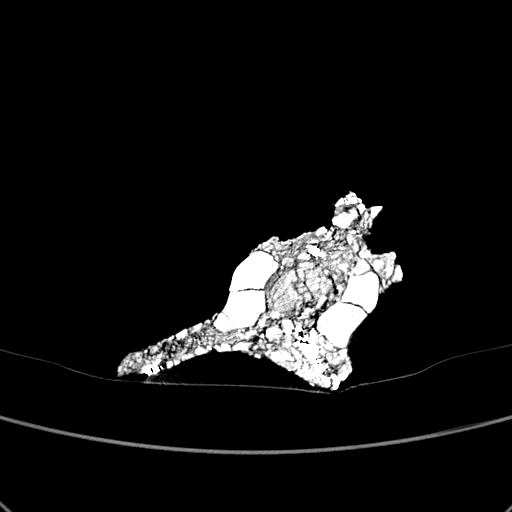

Supplement: S5 File — (ZIP) [file pone.0154403.s006.zip › S2_Files/WWCERATBC.Ser2.Img207.tif]

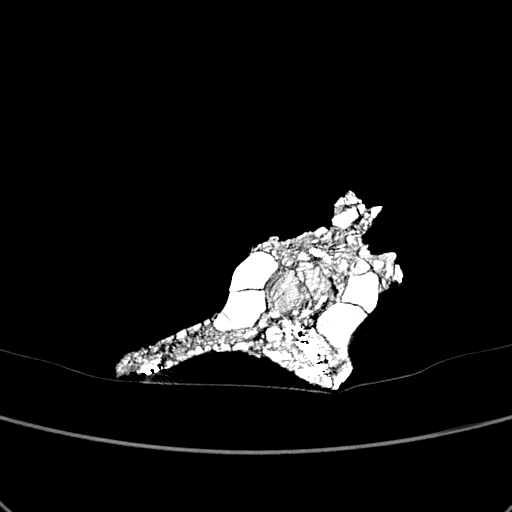

Supplement: S5 File — (ZIP) [file pone.0154403.s006.zip › S2_Files/WWCERATBC.Ser2.Img208.tif]

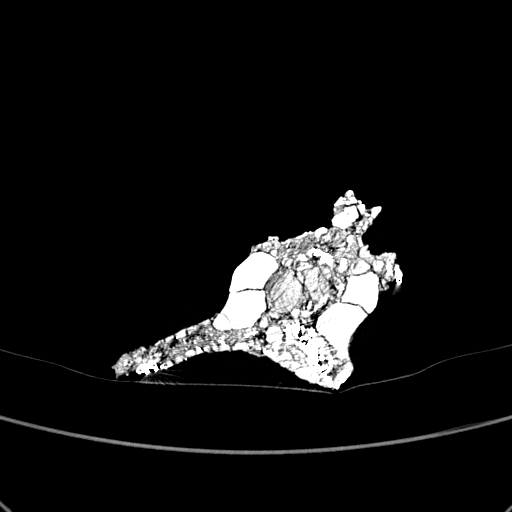

Supplement: S5 File — (ZIP) [file pone.0154403.s006.zip › S2_Files/WWCERATBC.Ser2.Img209.tif]

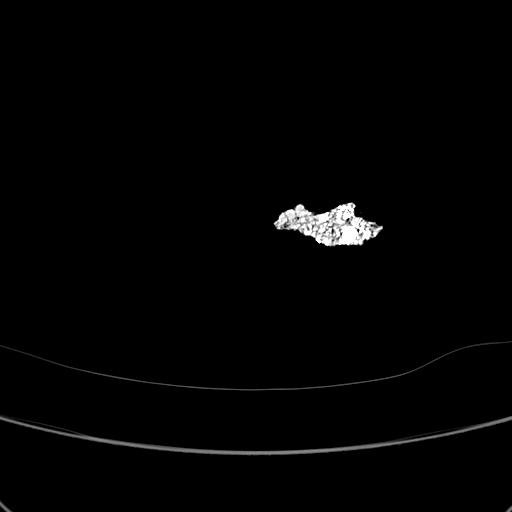

Supplement: S5 File — (ZIP) [file pone.0154403.s006.zip › S2_Files/WWCERATBC.Ser2.Img21.tif]

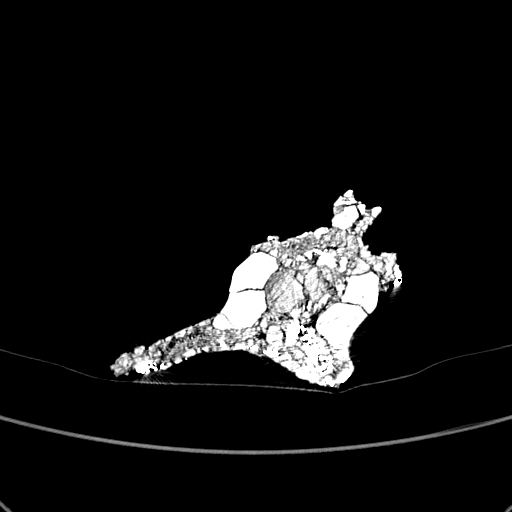

Supplement: S5 File — (ZIP) [file pone.0154403.s006.zip › S2_Files/WWCERATBC.Ser2.Img210.tif]

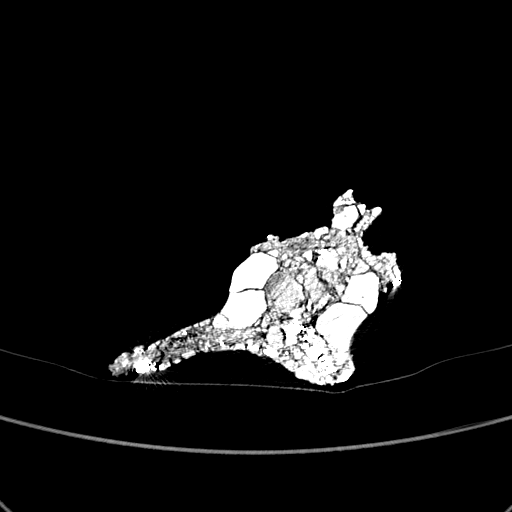

Supplement: S5 File — (ZIP) [file pone.0154403.s006.zip › S2_Files/WWCERATBC.Ser2.Img211.tif]

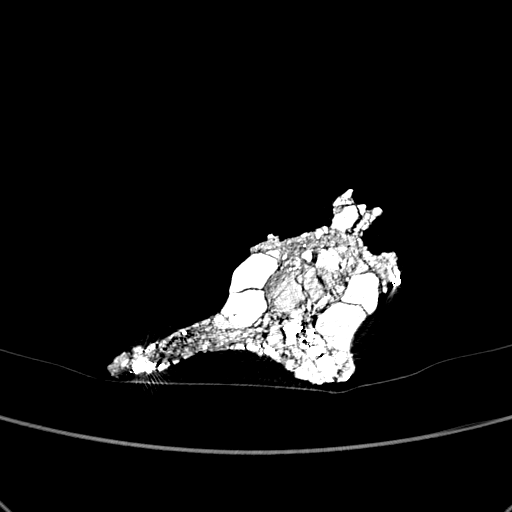

Supplement: S5 File — (ZIP) [file pone.0154403.s006.zip › S2_Files/WWCERATBC.Ser2.Img212.tif]

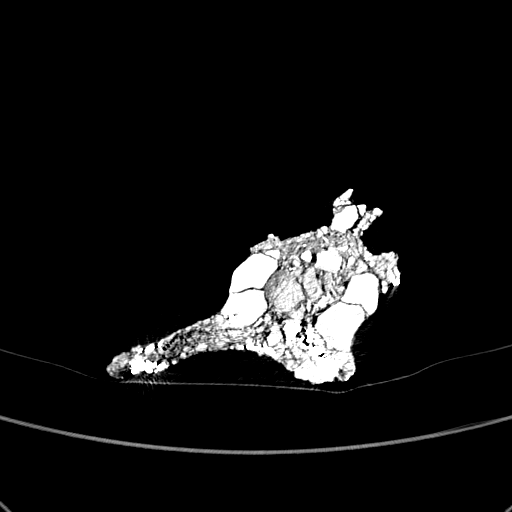

Supplement: S5 File — (ZIP) [file pone.0154403.s006.zip › S2_Files/WWCERATBC.Ser2.Img213.tif]

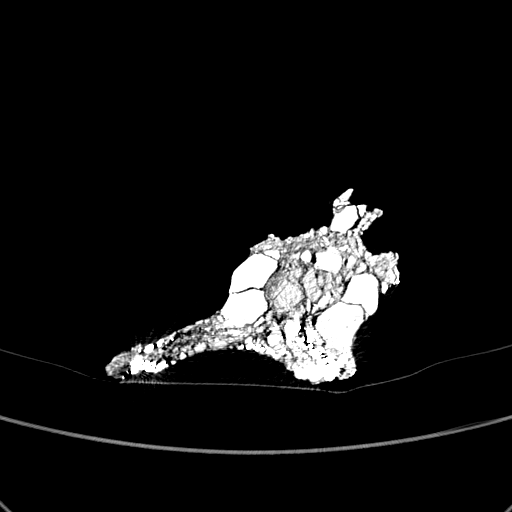

Supplement: S5 File — (ZIP) [file pone.0154403.s006.zip › S2_Files/WWCERATBC.Ser2.Img214.tif]

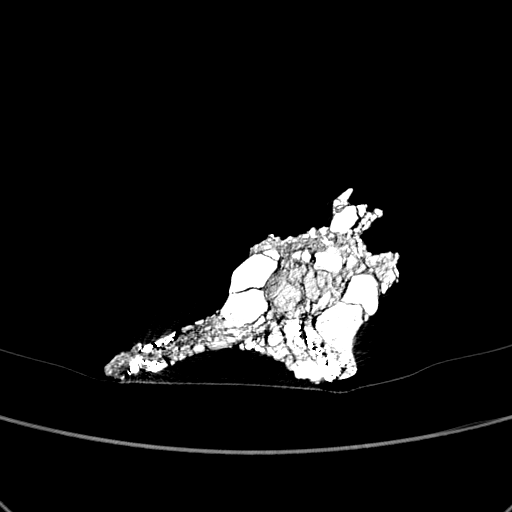

Supplement: S5 File — (ZIP) [file pone.0154403.s006.zip › S2_Files/WWCERATBC.Ser2.Img215.tif]

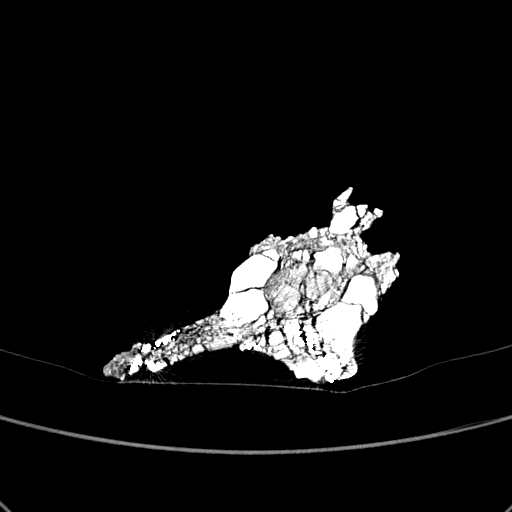

Supplement: S5 File — (ZIP) [file pone.0154403.s006.zip › S2_Files/WWCERATBC.Ser2.Img216.tif]

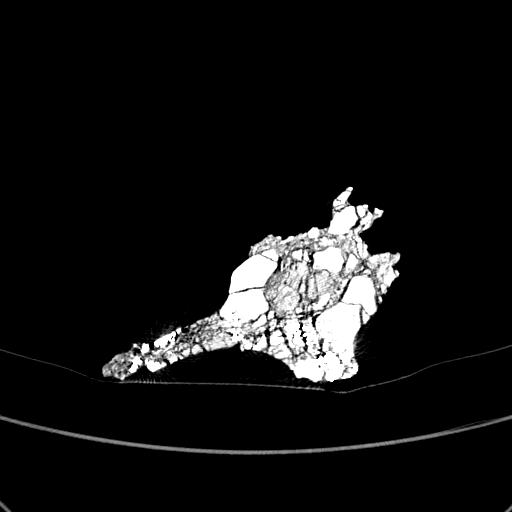

Supplement: S5 File — (ZIP) [file pone.0154403.s006.zip › S2_Files/WWCERATBC.Ser2.Img217.tif]

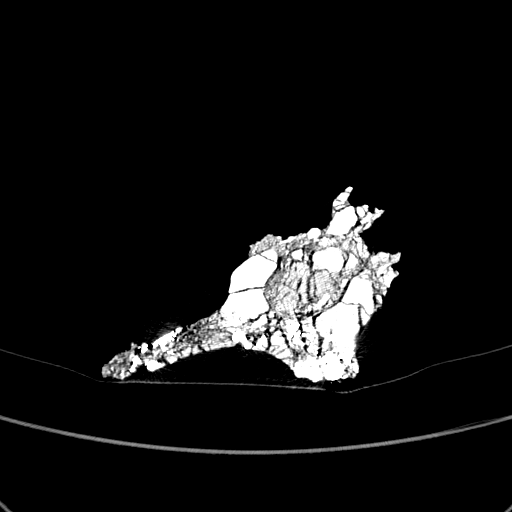

Supplement: S5 File — (ZIP) [file pone.0154403.s006.zip › S2_Files/WWCERATBC.Ser2.Img218.tif]

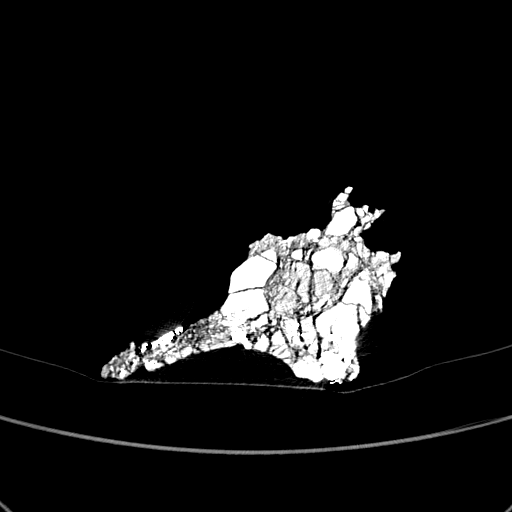

Supplement: S5 File — (ZIP) [file pone.0154403.s006.zip › S2_Files/WWCERATBC.Ser2.Img219.tif]

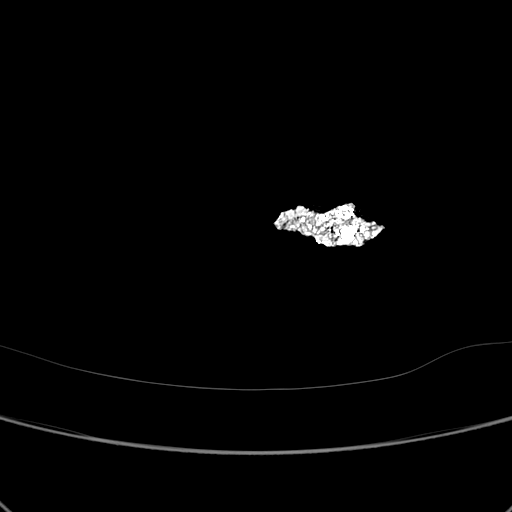

Supplement: S5 File — (ZIP) [file pone.0154403.s006.zip › S2_Files/WWCERATBC.Ser2.Img22.tif]

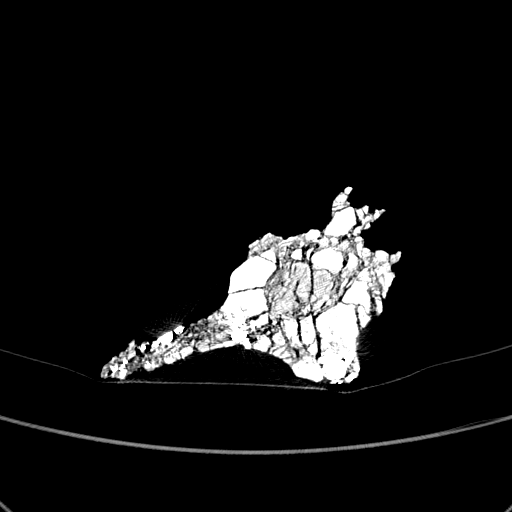

Supplement: S5 File — (ZIP) [file pone.0154403.s006.zip › S2_Files/WWCERATBC.Ser2.Img220.tif]

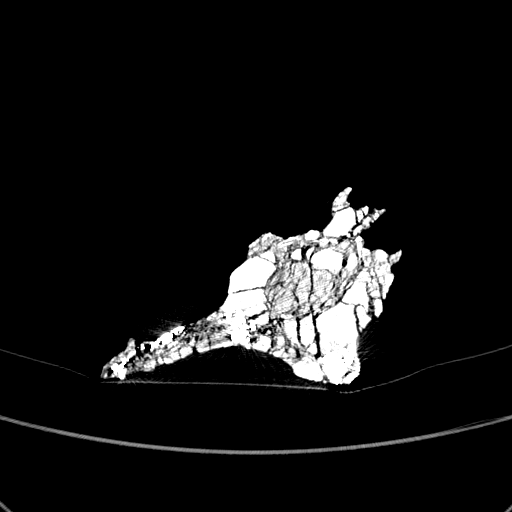

Supplement: S5 File — (ZIP) [file pone.0154403.s006.zip › S2_Files/WWCERATBC.Ser2.Img221.tif]

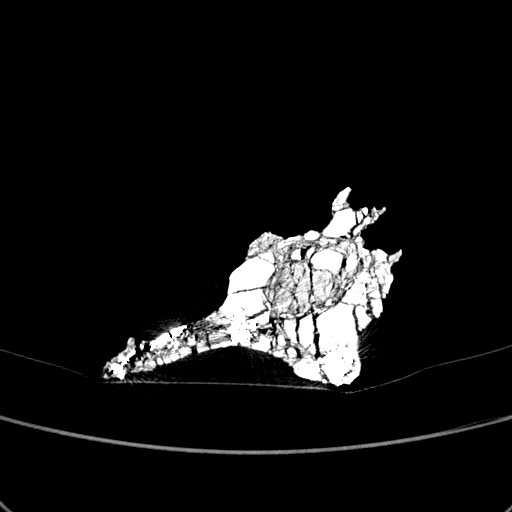

Supplement: S5 File — (ZIP) [file pone.0154403.s006.zip › S2_Files/WWCERATBC.Ser2.Img222.tif]

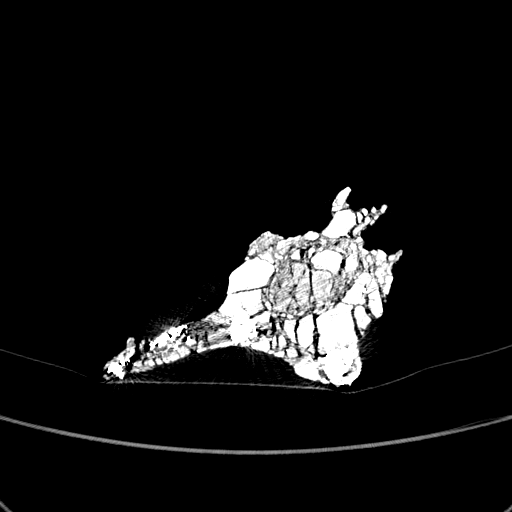

Supplement: S5 File — (ZIP) [file pone.0154403.s006.zip › S2_Files/WWCERATBC.Ser2.Img223.tif]

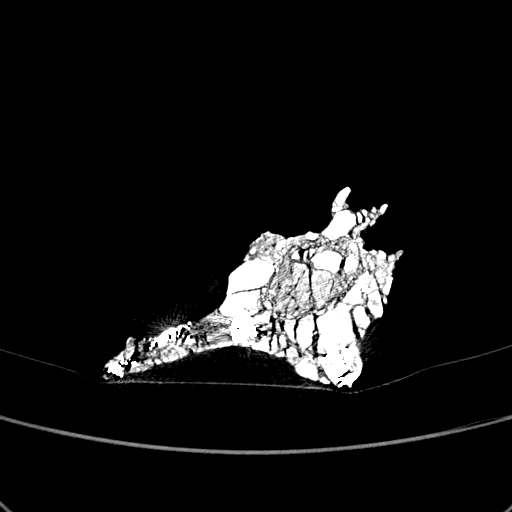

Supplement: S5 File — (ZIP) [file pone.0154403.s006.zip › S2_Files/WWCERATBC.Ser2.Img224.tif]

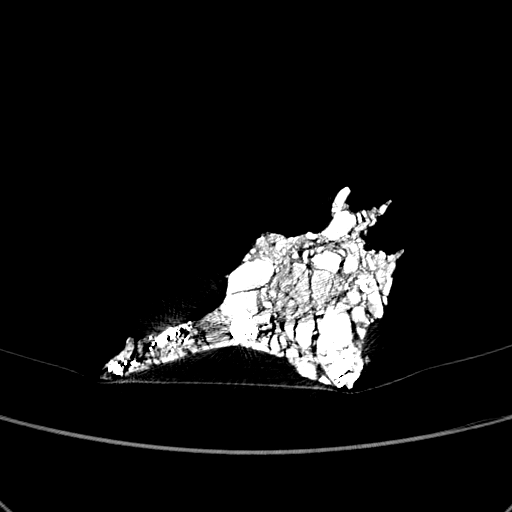

Supplement: S5 File — (ZIP) [file pone.0154403.s006.zip › S2_Files/WWCERATBC.Ser2.Img225.tif]

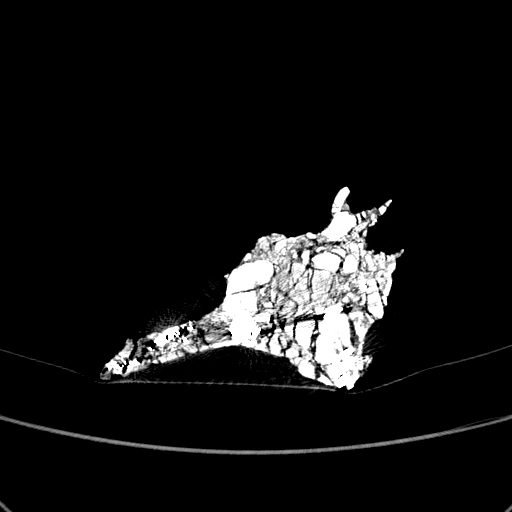

Supplement: S5 File — (ZIP) [file pone.0154403.s006.zip › S2_Files/WWCERATBC.Ser2.Img226.tif]

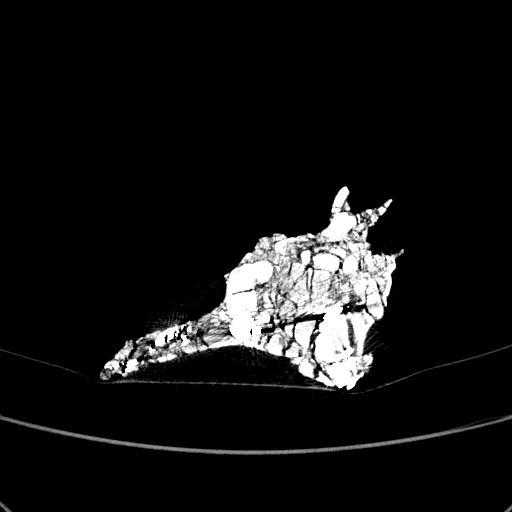

Supplement: S5 File — (ZIP) [file pone.0154403.s006.zip › S2_Files/WWCERATBC.Ser2.Img227.tif]

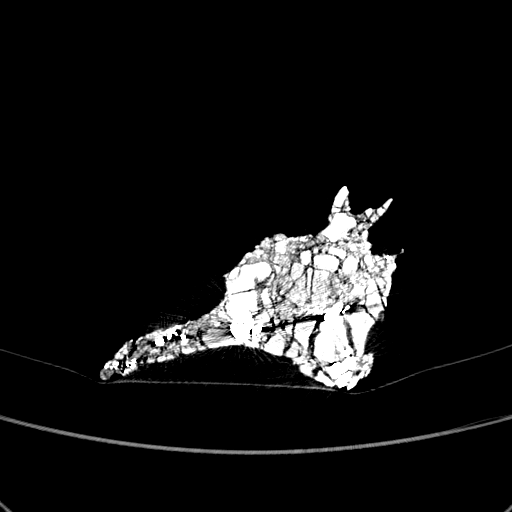

Supplement: S5 File — (ZIP) [file pone.0154403.s006.zip › S2_Files/WWCERATBC.Ser2.Img228.tif]

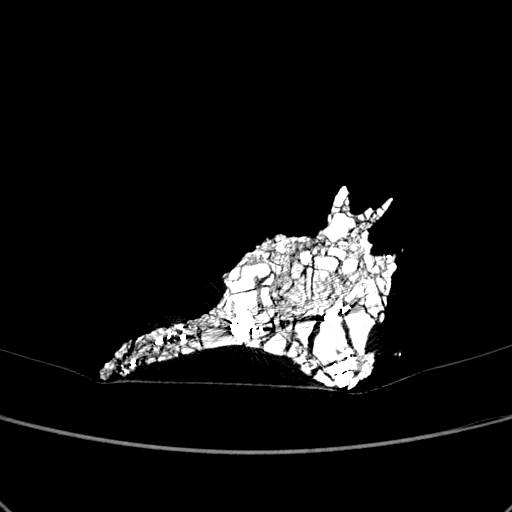

Supplement: S5 File — (ZIP) [file pone.0154403.s006.zip › S2_Files/WWCERATBC.Ser2.Img229.tif]

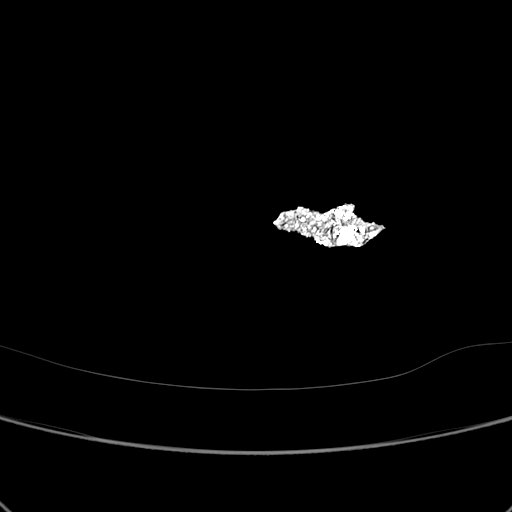

Supplement: S5 File — (ZIP) [file pone.0154403.s006.zip › S2_Files/WWCERATBC.Ser2.Img23.tif]

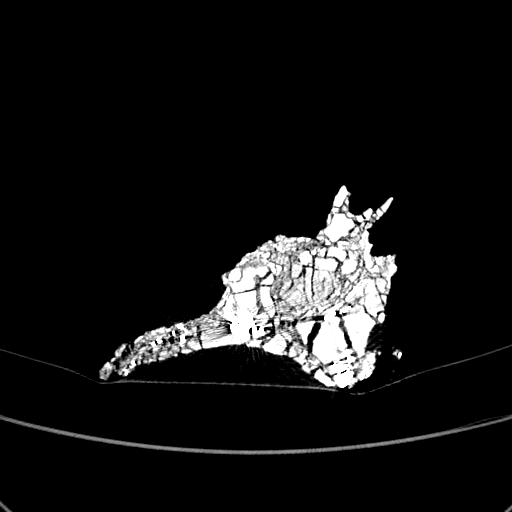

Supplement: S5 File — (ZIP) [file pone.0154403.s006.zip › S2_Files/WWCERATBC.Ser2.Img230.tif]

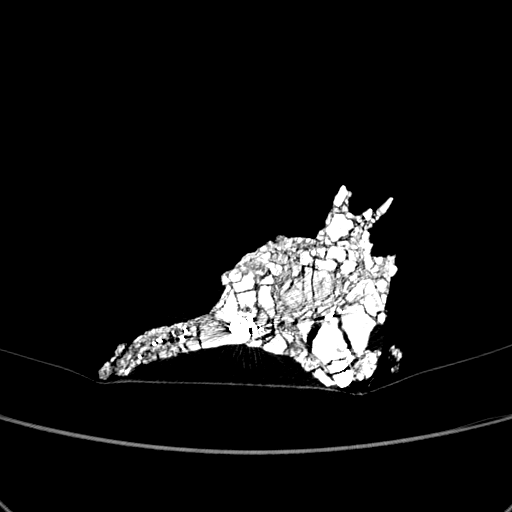

Supplement: S5 File — (ZIP) [file pone.0154403.s006.zip › S2_Files/WWCERATBC.Ser2.Img231.tif]

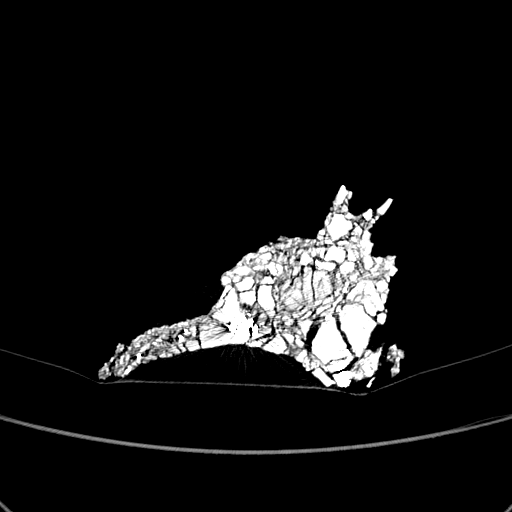

Supplement: S5 File — (ZIP) [file pone.0154403.s006.zip › S2_Files/WWCERATBC.Ser2.Img232.tif]

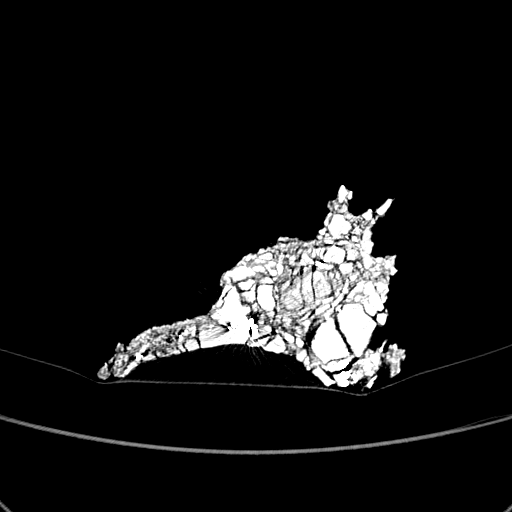

Supplement: S5 File — (ZIP) [file pone.0154403.s006.zip › S2_Files/WWCERATBC.Ser2.Img233.tif]

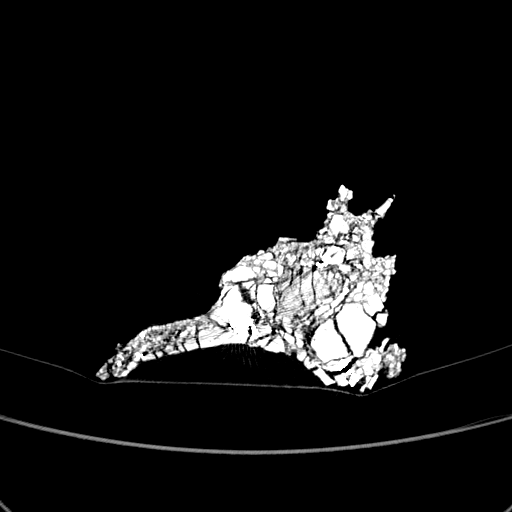

Supplement: S5 File — (ZIP) [file pone.0154403.s006.zip › S2_Files/WWCERATBC.Ser2.Img234.tif]

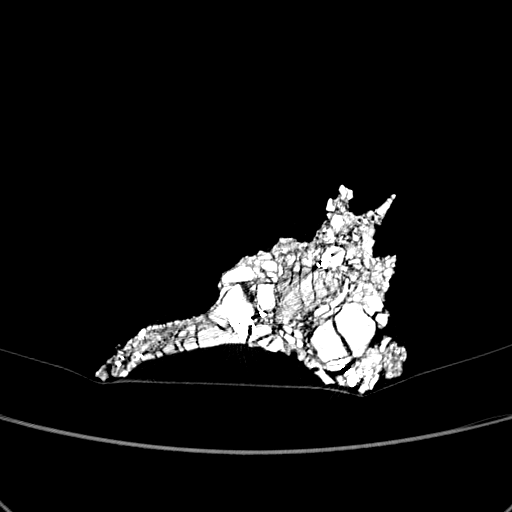

Supplement: S5 File — (ZIP) [file pone.0154403.s006.zip › S2_Files/WWCERATBC.Ser2.Img235.tif]

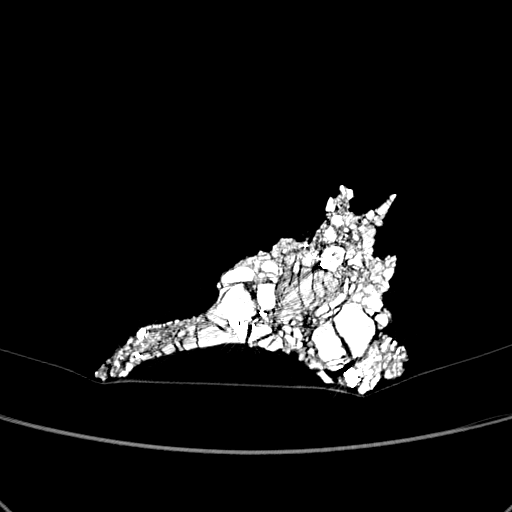

Supplement: S5 File — (ZIP) [file pone.0154403.s006.zip › S2_Files/WWCERATBC.Ser2.Img236.tif]

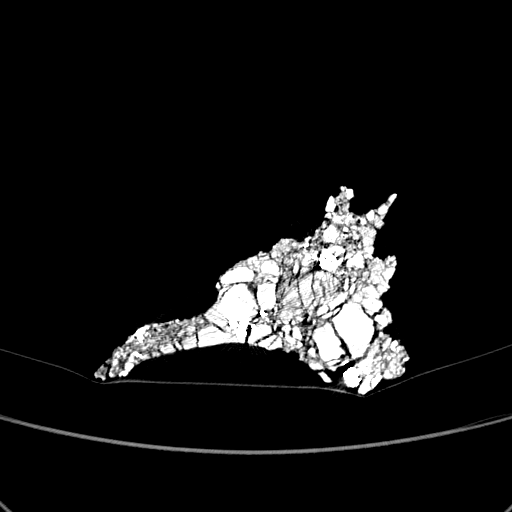

Supplement: S5 File — (ZIP) [file pone.0154403.s006.zip › S2_Files/WWCERATBC.Ser2.Img237.tif]

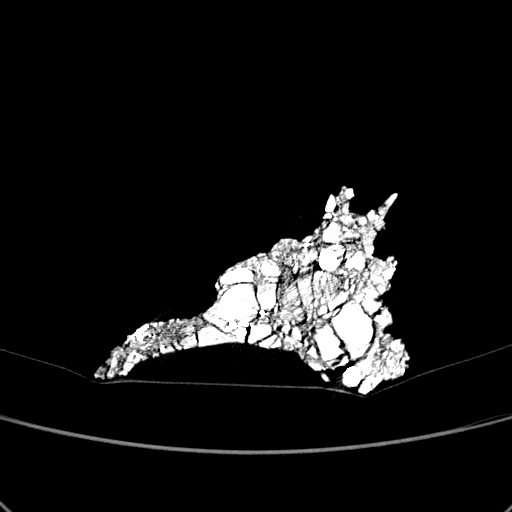

Supplement: S5 File — (ZIP) [file pone.0154403.s006.zip › S2_Files/WWCERATBC.Ser2.Img238.tif]

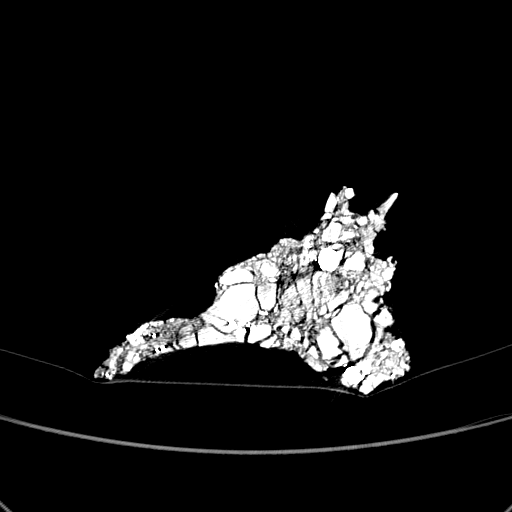

Supplement: S5 File — (ZIP) [file pone.0154403.s006.zip › S2_Files/WWCERATBC.Ser2.Img239.tif]

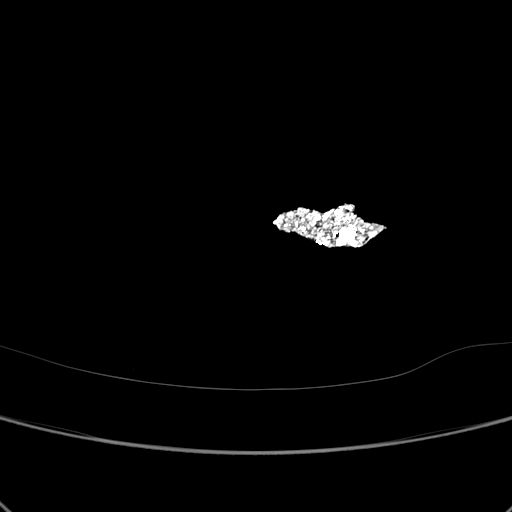

Supplement: S5 File — (ZIP) [file pone.0154403.s006.zip › S2_Files/WWCERATBC.Ser2.Img24.tif]

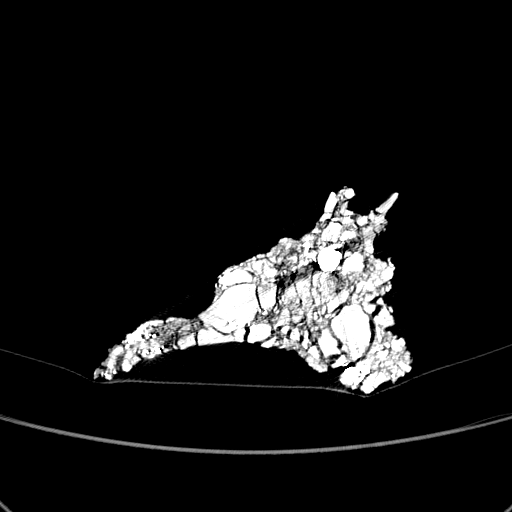

Supplement: S5 File — (ZIP) [file pone.0154403.s006.zip › S2_Files/WWCERATBC.Ser2.Img240.tif]

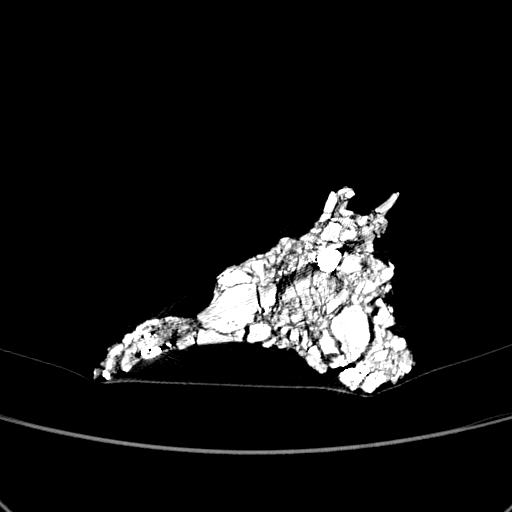

Supplement: S5 File — (ZIP) [file pone.0154403.s006.zip › S2_Files/WWCERATBC.Ser2.Img241.tif]

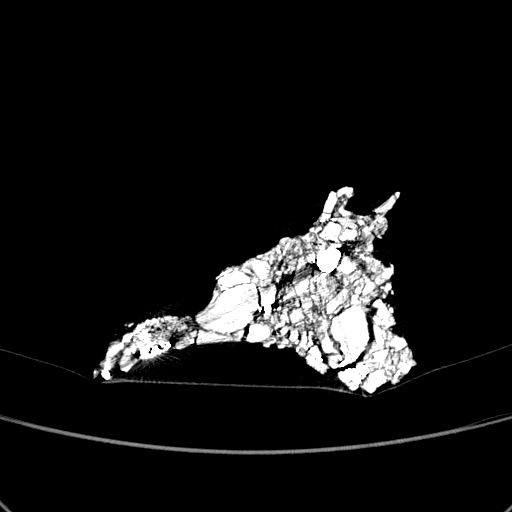

Supplement: S5 File — (ZIP) [file pone.0154403.s006.zip › S2_Files/WWCERATBC.Ser2.Img242.tif]

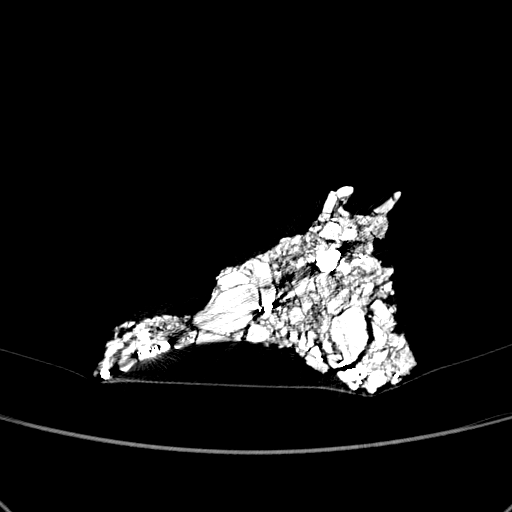

Supplement: S5 File — (ZIP) [file pone.0154403.s006.zip › S2_Files/WWCERATBC.Ser2.Img243.tif]

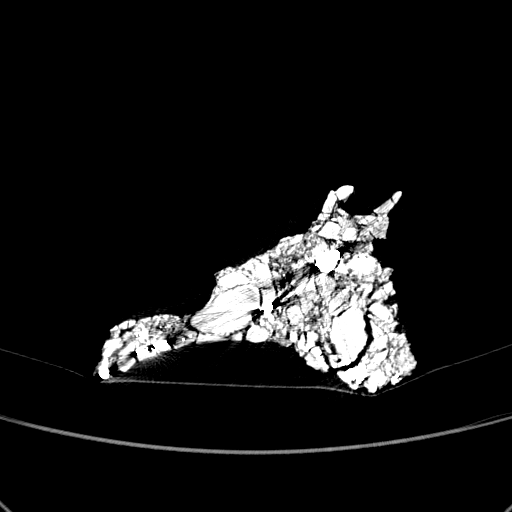

Supplement: S5 File — (ZIP) [file pone.0154403.s006.zip › S2_Files/WWCERATBC.Ser2.Img244.tif]

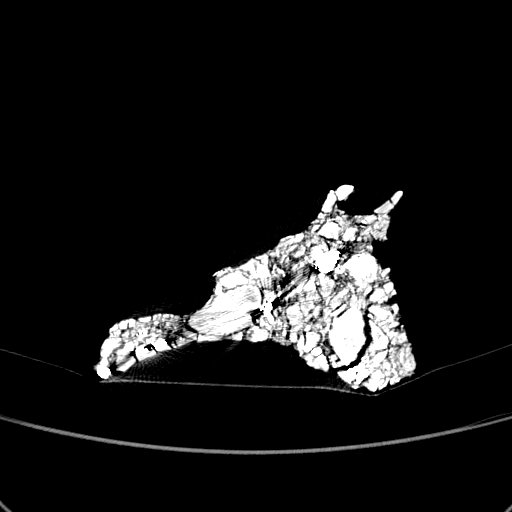

Supplement: S5 File — (ZIP) [file pone.0154403.s006.zip › S2_Files/WWCERATBC.Ser2.Img245.tif]

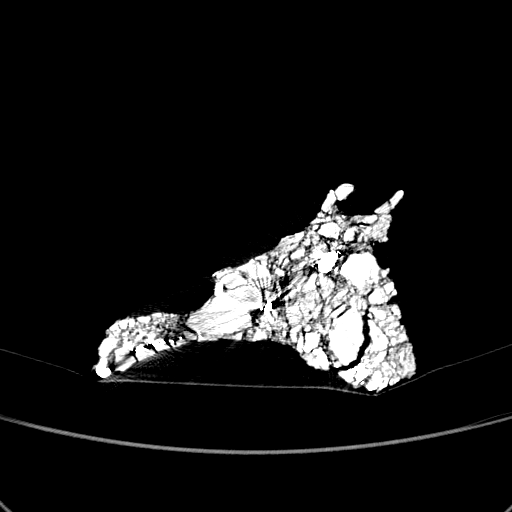

Supplement: S5 File — (ZIP) [file pone.0154403.s006.zip › S2_Files/WWCERATBC.Ser2.Img246.tif]

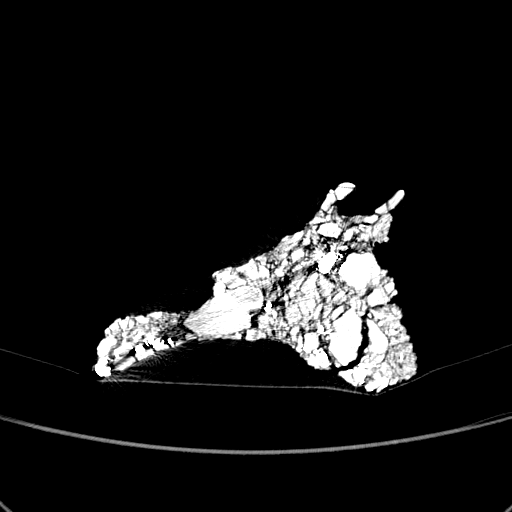

Supplement: S5 File — (ZIP) [file pone.0154403.s006.zip › S2_Files/WWCERATBC.Ser2.Img247.tif]

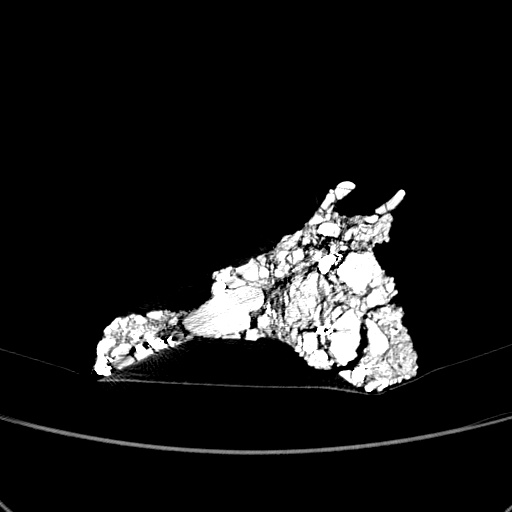

Supplement: S5 File — (ZIP) [file pone.0154403.s006.zip › S2_Files/WWCERATBC.Ser2.Img248.tif]

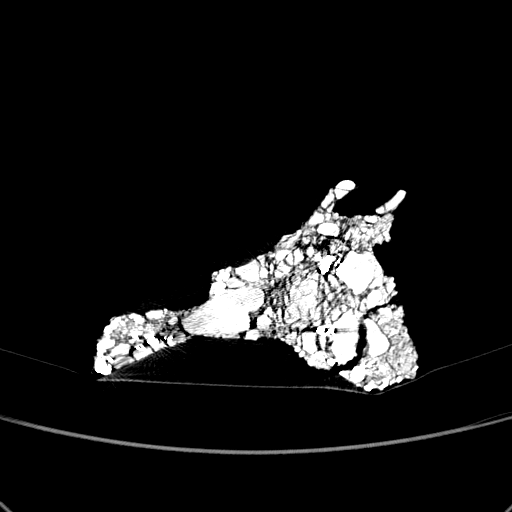

Supplement: S5 File — (ZIP) [file pone.0154403.s006.zip › S2_Files/WWCERATBC.Ser2.Img249.tif]

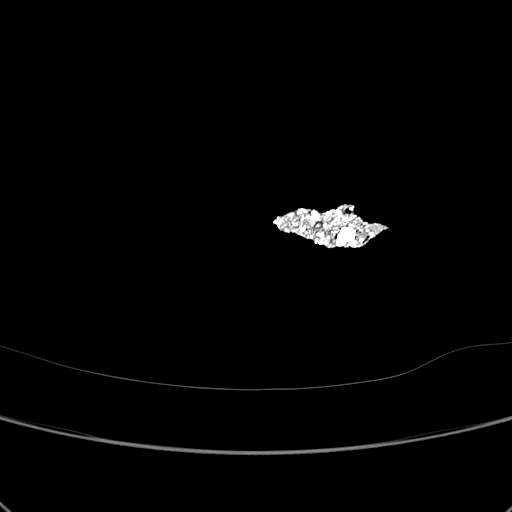

Supplement: S5 File — (ZIP) [file pone.0154403.s006.zip › S2_Files/WWCERATBC.Ser2.Img25.tif]

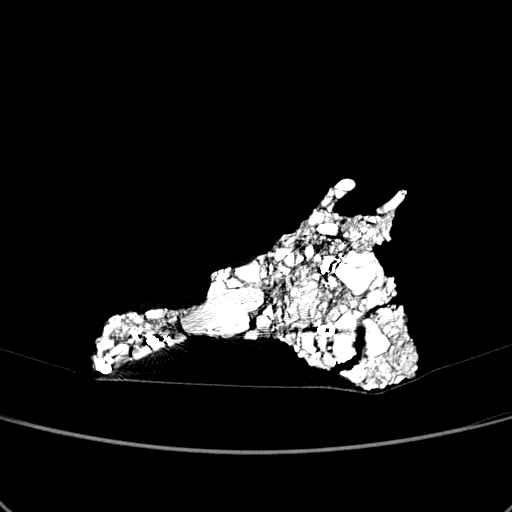

Supplement: S5 File — (ZIP) [file pone.0154403.s006.zip › S2_Files/WWCERATBC.Ser2.Img250.tif]

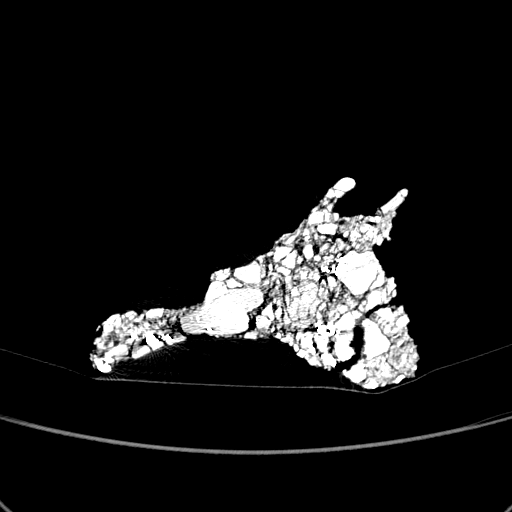

Supplement: S5 File — (ZIP) [file pone.0154403.s006.zip › S2_Files/WWCERATBC.Ser2.Img251.tif]

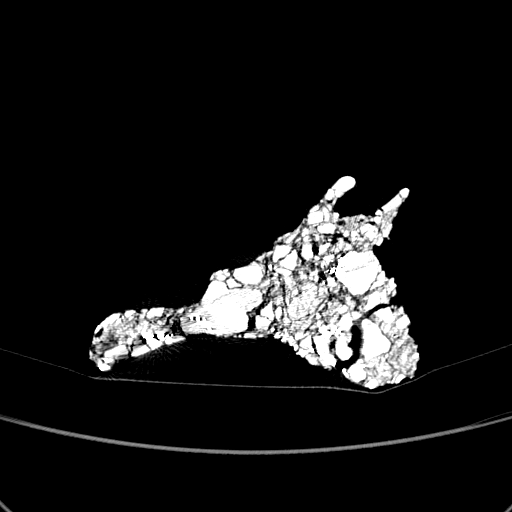

Supplement: S5 File — (ZIP) [file pone.0154403.s006.zip › S2_Files/WWCERATBC.Ser2.Img252.tif]

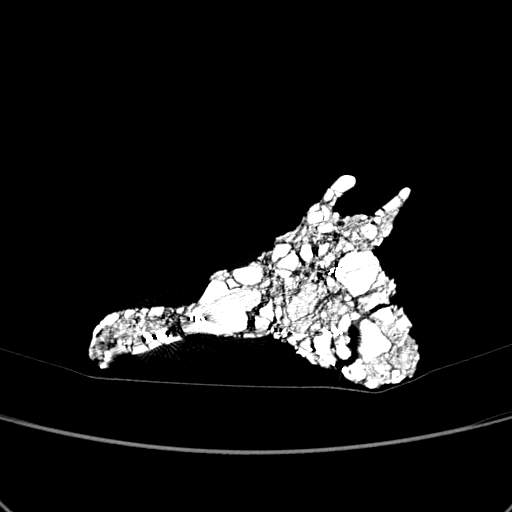

Supplement: S5 File — (ZIP) [file pone.0154403.s006.zip › S2_Files/WWCERATBC.Ser2.Img253.tif]

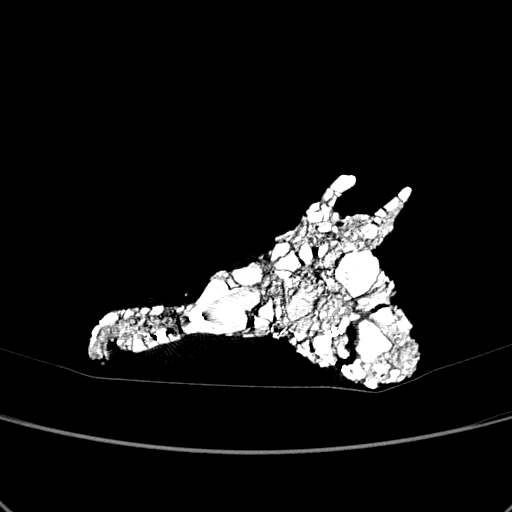

Supplement: S5 File — (ZIP) [file pone.0154403.s006.zip › S2_Files/WWCERATBC.Ser2.Img254.tif]

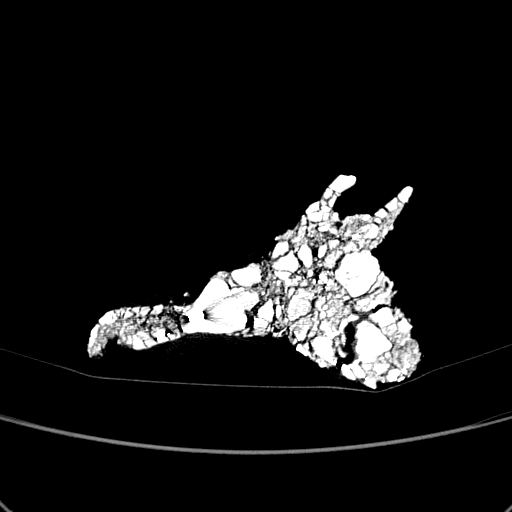

Supplement: S5 File — (ZIP) [file pone.0154403.s006.zip › S2_Files/WWCERATBC.Ser2.Img255.tif]

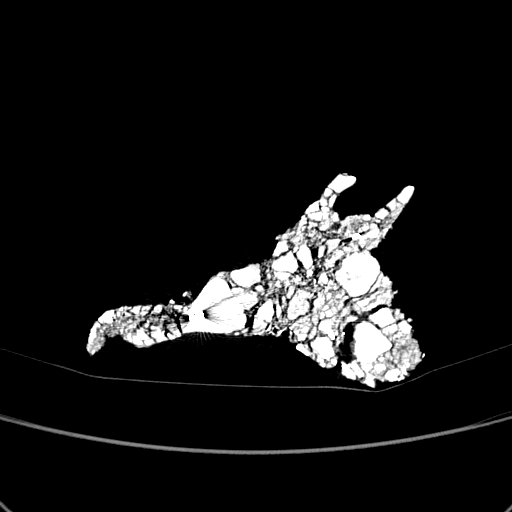

Supplement: S5 File — (ZIP) [file pone.0154403.s006.zip › S2_Files/WWCERATBC.Ser2.Img256.tif]

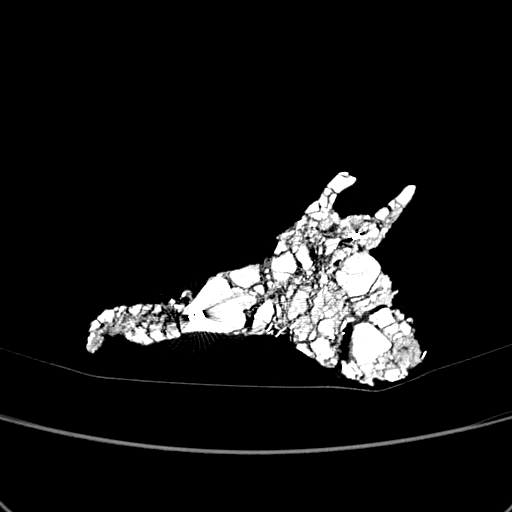

Supplement: S5 File — (ZIP) [file pone.0154403.s006.zip › S2_Files/WWCERATBC.Ser2.Img257.tif]

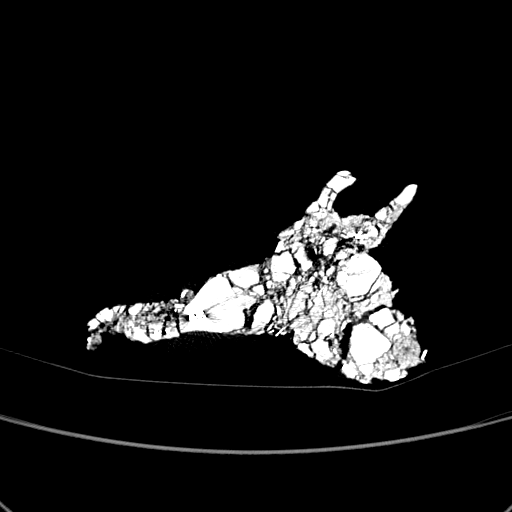

Supplement: S5 File — (ZIP) [file pone.0154403.s006.zip › S2_Files/WWCERATBC.Ser2.Img258.tif]

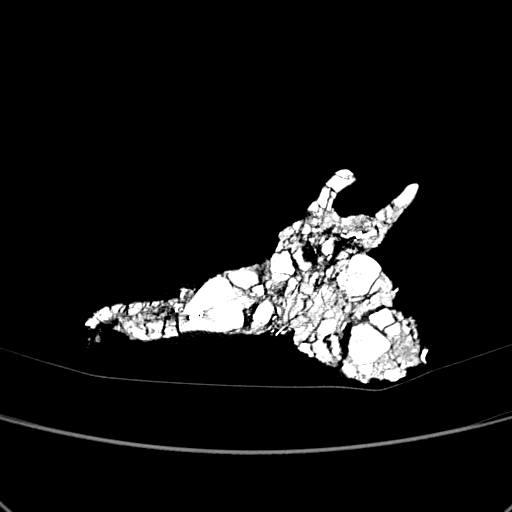

Supplement: S5 File — (ZIP) [file pone.0154403.s006.zip › S2_Files/WWCERATBC.Ser2.Img259.tif]

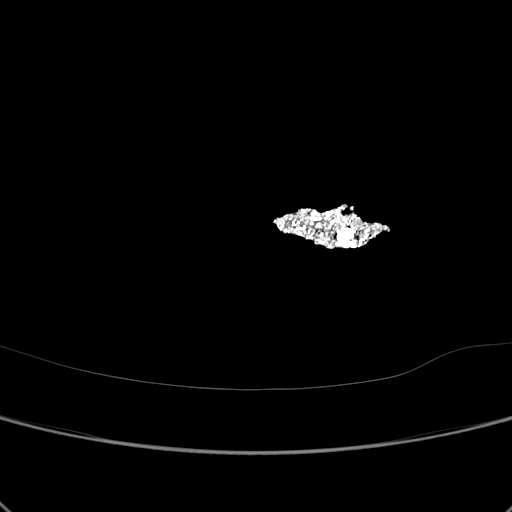

Supplement: S5 File — (ZIP) [file pone.0154403.s006.zip › S2_Files/WWCERATBC.Ser2.Img26.tif]

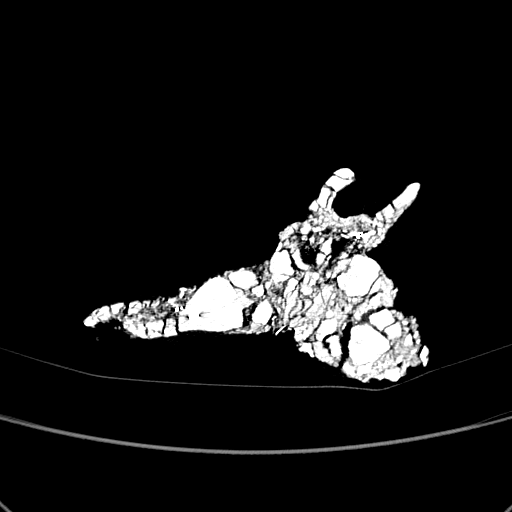

Supplement: S5 File — (ZIP) [file pone.0154403.s006.zip › S2_Files/WWCERATBC.Ser2.Img260.tif]

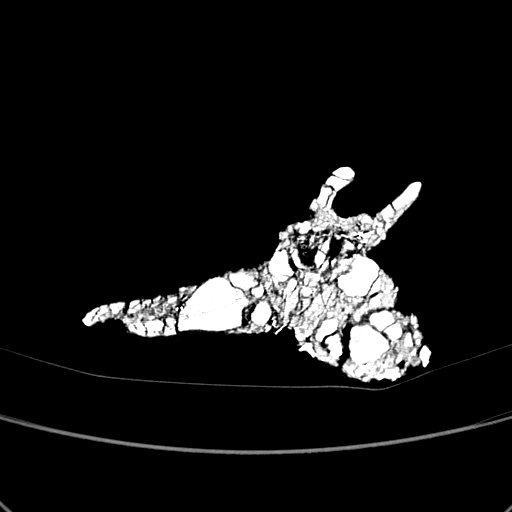

Supplement: S5 File — (ZIP) [file pone.0154403.s006.zip › S2_Files/WWCERATBC.Ser2.Img261.tif]

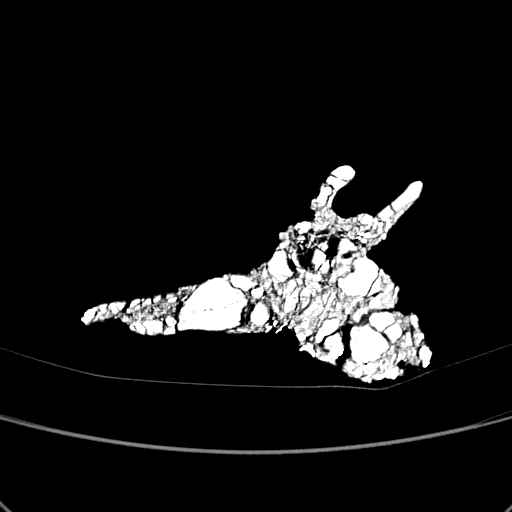

Supplement: S5 File — (ZIP) [file pone.0154403.s006.zip › S2_Files/WWCERATBC.Ser2.Img262.tif]

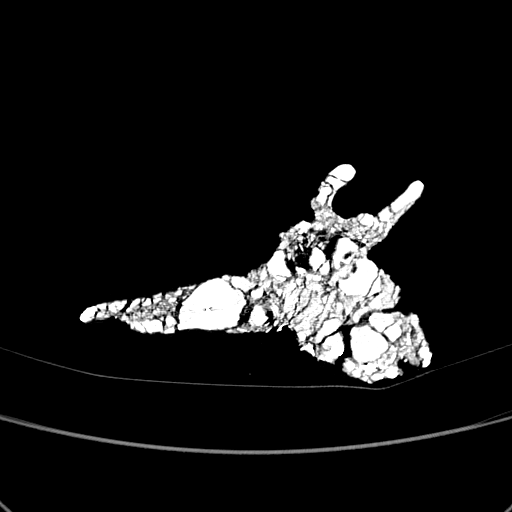

Supplement: S5 File — (ZIP) [file pone.0154403.s006.zip › S2_Files/WWCERATBC.Ser2.Img263.tif]

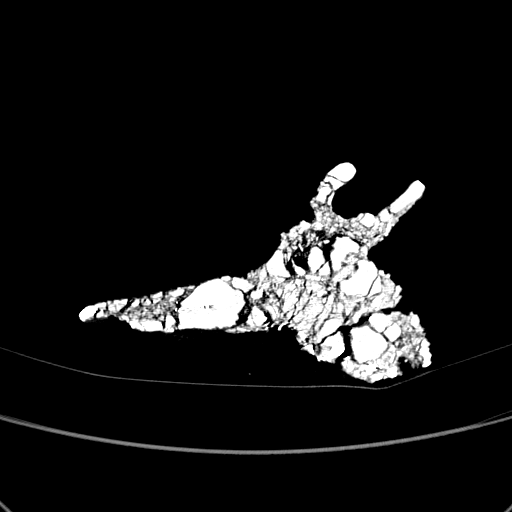

Supplement: S5 File — (ZIP) [file pone.0154403.s006.zip › S2_Files/WWCERATBC.Ser2.Img264.tif]

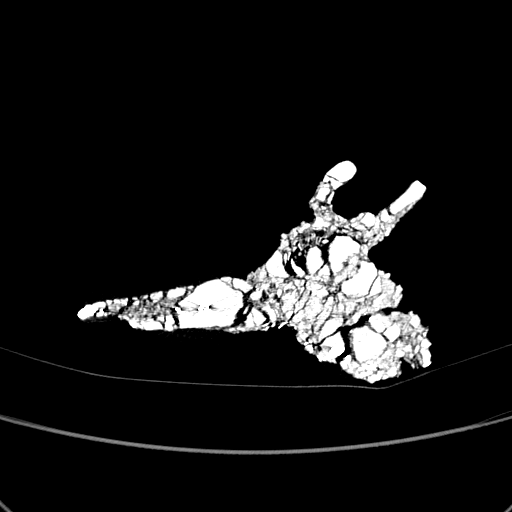

Supplement: S5 File — (ZIP) [file pone.0154403.s006.zip › S2_Files/WWCERATBC.Ser2.Img265.tif]

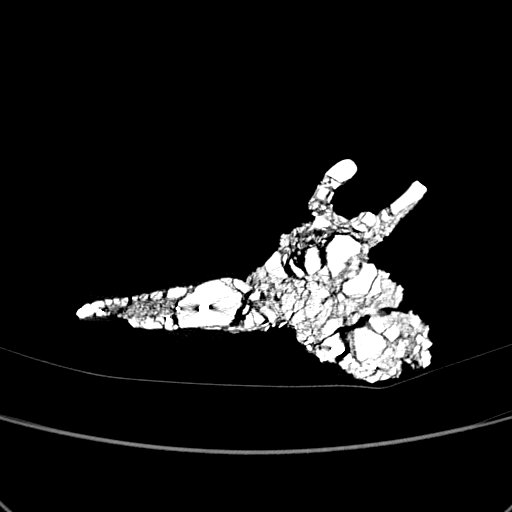

Supplement: S5 File — (ZIP) [file pone.0154403.s006.zip › S2_Files/WWCERATBC.Ser2.Img266.tif]

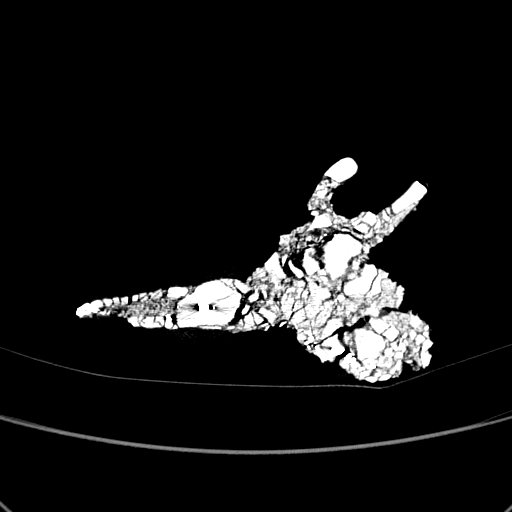

Supplement: S5 File — (ZIP) [file pone.0154403.s006.zip › S2_Files/WWCERATBC.Ser2.Img267.tif]

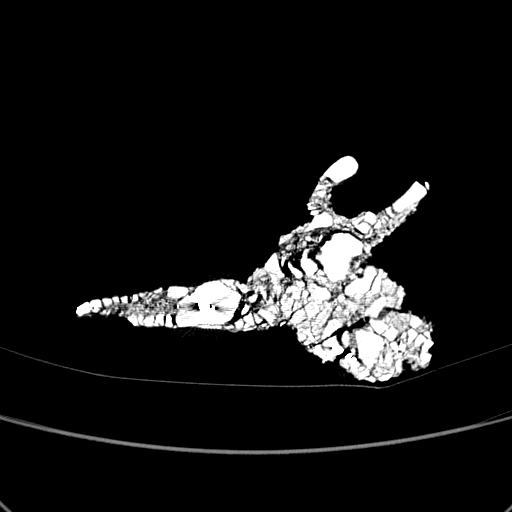

Supplement: S5 File — (ZIP) [file pone.0154403.s006.zip › S2_Files/WWCERATBC.Ser2.Img268.tif]

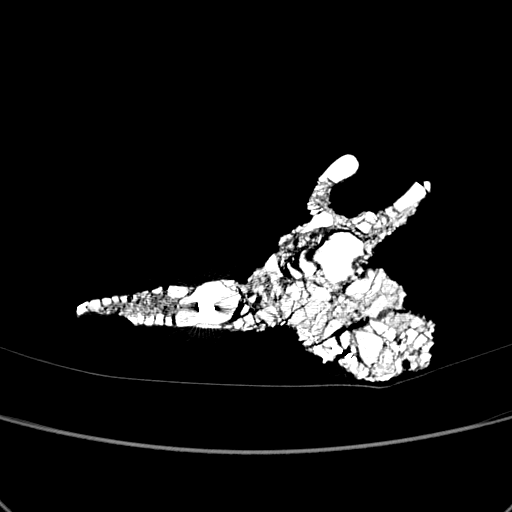

Supplement: S5 File — (ZIP) [file pone.0154403.s006.zip › S2_Files/WWCERATBC.Ser2.Img269.tif]

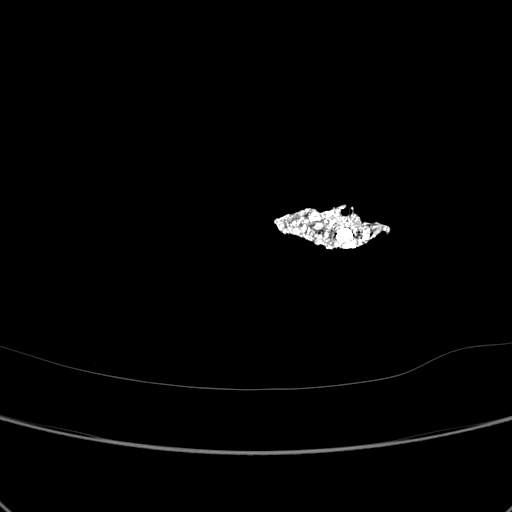

Supplement: S5 File — (ZIP) [file pone.0154403.s006.zip › S2_Files/WWCERATBC.Ser2.Img27.tif]

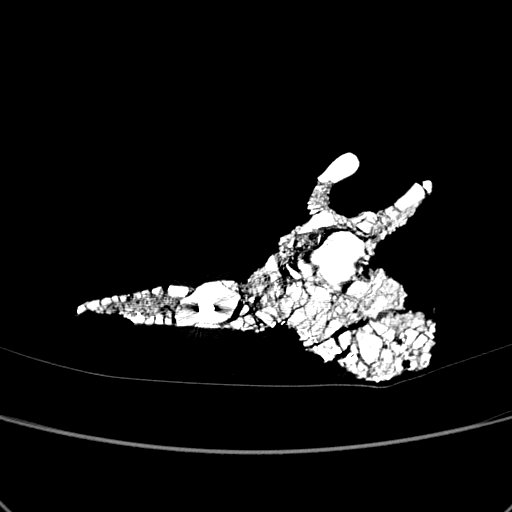

Supplement: S5 File — (ZIP) [file pone.0154403.s006.zip › S2_Files/WWCERATBC.Ser2.Img270.tif]

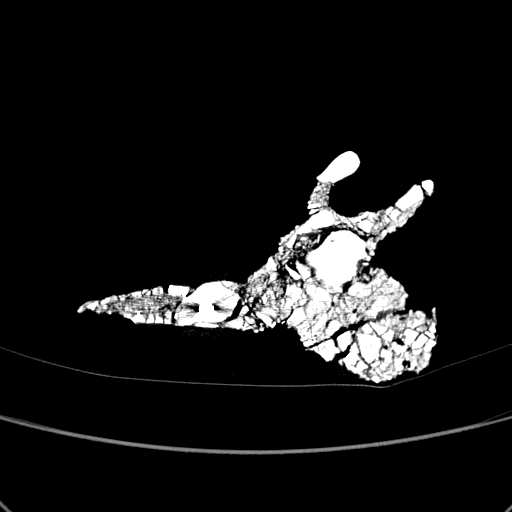

Supplement: S5 File — (ZIP) [file pone.0154403.s006.zip › S2_Files/WWCERATBC.Ser2.Img271.tif]

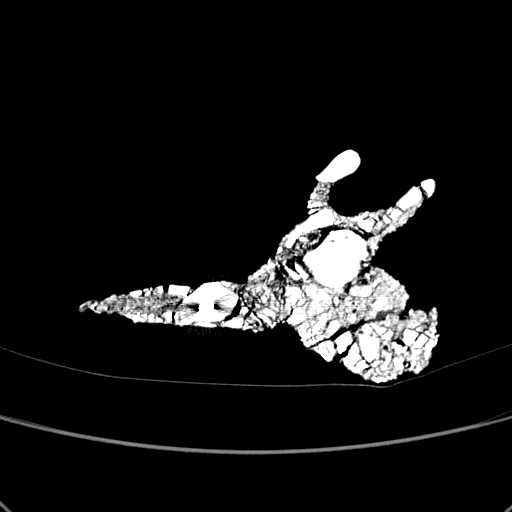

Supplement: S5 File — (ZIP) [file pone.0154403.s006.zip › S2_Files/WWCERATBC.Ser2.Img272.tif]

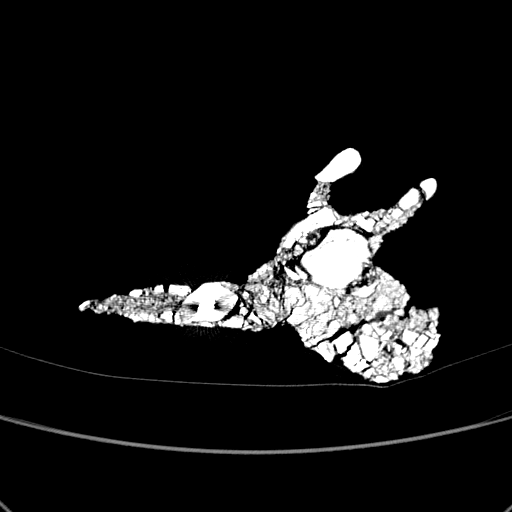

Supplement: S5 File — (ZIP) [file pone.0154403.s006.zip › S2_Files/WWCERATBC.Ser2.Img273.tif]

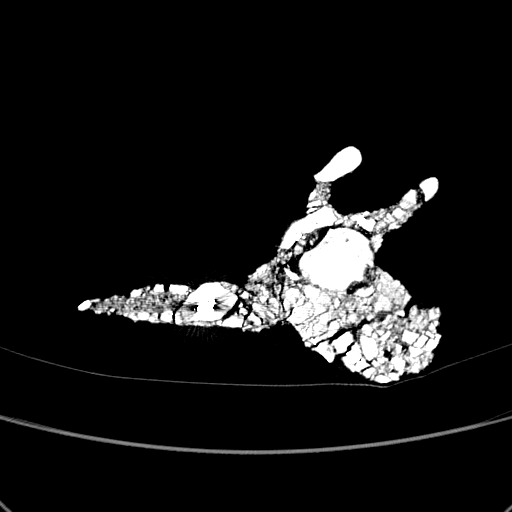

Supplement: S5 File — (ZIP) [file pone.0154403.s006.zip › S2_Files/WWCERATBC.Ser2.Img274.tif]

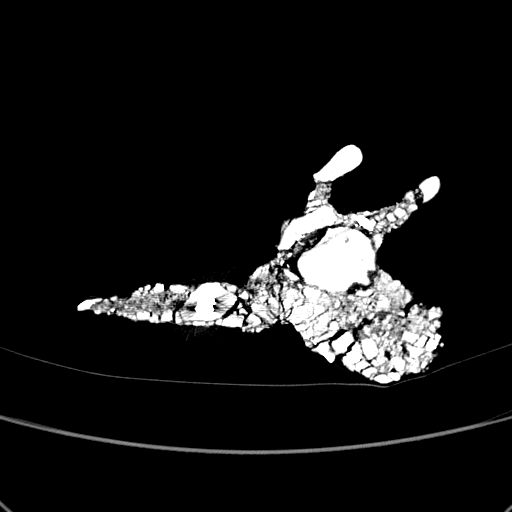

Supplement: S5 File — (ZIP) [file pone.0154403.s006.zip › S2_Files/WWCERATBC.Ser2.Img275.tif]

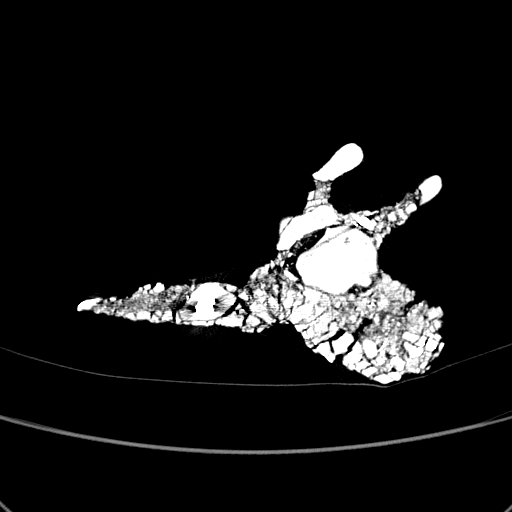

Supplement: S5 File — (ZIP) [file pone.0154403.s006.zip › S2_Files/WWCERATBC.Ser2.Img276.tif]

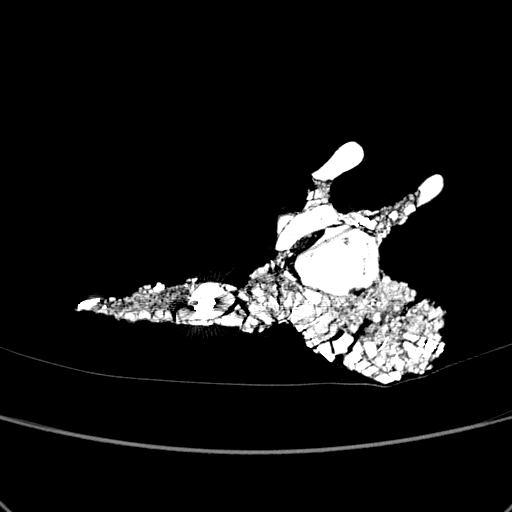

Supplement: S5 File — (ZIP) [file pone.0154403.s006.zip › S2_Files/WWCERATBC.Ser2.Img277.tif]
