# Supplementary material for: A New Centrosaurine Ceratopsid, Machairoceratops cronusi gen et sp. nov., from the Upper Sand Member of the Wahweap Formation (Middle Campanian), Southern Utah
Source: PLoS One. 2016 May 18;11(5):e0154403. doi: 10.1371/journal.pone.0154403 (PMC4871575; doi:10.1371/journal.pone.0154403)
Supplement: S1 Table — (DOCX) [file pone.0154403.s007.docx]

S1 Table. Character-taxon matrix. **A**, (0,1); **B**, (1,3); **C**, (2,3).

*Leptoceratops gracilis*

| 10 | 20 | 30 | 40 | 50 | 60 | 70 |
| --- | --- | --- | --- | --- | --- | --- |
| 1--0---001 | 0001-00?0- | -0-00----- | 000-0000-0 | 0000000-00 | --------0- | ---------- |
| 80 | 90 | 100 | 101 |  |  |  |
| --00000000 | 0000000000 | 0000000000 | 0 |  |  |  |

*Protoceratops andrewsi*

| 10 | 20 | 30 | 40 | 50 | 60 | 70 |
| --- | --- | --- | --- | --- | --- | --- |
| 0--0---000 | 0000100000 | 00-0?--?-- | 000-0000-0 | 0100000-00 | 00-0----0- | ---------- |
| 80 | 90 | 100 | 101 |  |  |  |
| --00000000 | 0000000000 | 0000000000 | 0 |  |  |  |

*Magnirostris dodsoni*

| 10 | 20 | 30 | 40 | 50 | 60 | 70 |
| --- | --- | --- | --- | --- | --- | --- |
| 1--0---000 | 1100000001 | 00-?1000-- | 000-0000-? | ?????0???? | --------?- | ??-------- |
| 80 | 90 | 100 | 101 |  |  |  |
| --100?0?0? | ?????????? | ?????????? | ? |  |  |  |

*Bagaceratops rozhdestvenskyi*

| 10 | 20 | 30 | 40 | 50 | 60 | 70 |
| --- | --- | --- | --- | --- | --- | --- |
| 1--0---000 | 1000000000 | 00-?0----- | 000-0000-0 | 01???00-?0 | --------?- | ??-------- |
| 80 | 90 | 100 | 101 |  |  |  |
| --0010000? | ?????????? | ?????????? | ? |  |  |  |

*Turanoceratops tardabilis*

| 10 | 20 | 30 | 40 | 50 | 60 | 70 |
| --- | --- | --- | --- | --- | --- | --- |
| ?????????? | ?????0000? | ??-?0001-1 | ?1?????0-? | ?????????? | ?????????? | ?????????? |
| 80 | 90 | 100 | 101 |  |  |  |
| ???00????0 | ?????????? | ?????????? | ? |  |  |  |

*Zuniceratops christopheri*

| 10 | 20 | 30 | 40 | 50 | 60 | 70 |
| --- | --- | --- | --- | --- | --- | --- |
| 1000000??? | 010000000- | -100000102 | ?1101100-? | 0?0?????00 | 10-0----?- | ??-------- |
| 80 | 90 | 100 | 101 |  |  |  |
| --00000000 | ???110???? | ?????01?0? | ? |  |  |  |

*Chasmosaurus belli*

| 10 | 20 | 30 | 40 | 50 | 60 | 70 |
| --- | --- | --- | --- | --- | --- | --- |
| 100111000- | 0100111111 | 101?000A02 | 0111111111 | 1011111110 | 0001110001 | 020300-0-0 |
| 80 | 90 | 100 | 101 |  |  |  |
| -011011111 | 1111111111 | 1111111111 | 1 |  |  |  |

*Pentaceratops sternbergii*

| 10 | 20 | 30 | 40 | 50 | 60 | 70 |
| --- | --- | --- | --- | --- | --- | --- |
| 100111000- | 0110111111 | 1010001112 | 0111111111 | 1011111111 | 0001110001 | 020300-0-0 |
| 80 | 90 | 100 | 101 |  |  |  |
| -011011111 | 1111111111 | 1111111?11 | 1 |  |  |  |

*Diabloceratops eatoni*

| 10 | 20 | 30 | 40 | 50 | 60 | 70 |
| --- | --- | --- | --- | --- | --- | --- |
| 0110000101 | 0010000-00 | 01??000102 | ?110110100 | 0110101011 | 1111001101 | 1100010101 |
| 80 | 90 | 100 | 101 |  |  |  |
| 011??????1 | ?????????? | ?????????? | ? |  |  |  |

*Albertaceratops nesmoi*

| 10 | 20 | 30 | 40 | 50 | 60 | 70 |
| --- | --- | --- | --- | --- | --- | --- |
| ?1?000???? | ??01?101?0 | 0???000112 | ?111111??0 | 0110101011 | 1111001101 | 2110000101 |
| 80 | 90 | 100 | 101 |  |  |  |
| 01?1101111 | ?????????? | ?????????? | ? |  |  |  |

*Rubeosaurus ovatus*

| 10 | 20 | 30 | 40 | 50 | 60 | 70 |
| --- | --- | --- | --- | --- | --- | --- |
| 01??0001?1 | ??0??????1 | ??0?000022 | ?1???????? | ??????101? | ?1?100?100 | ??00101111 |
| 80 | 90 | 100 | 101 |  |  |  |
| ?1???????? | ?????????? | ?????????? | ? |  |  |  |

*Styracosaurus albertensis*

| 10 | 20 | 30 | 40 | 50 | 60 | 70 |
| --- | --- | --- | --- | --- | --- | --- |
| 0110000111 | 1001-11111 | 0100000022 | 1111111100 | 0110101011 | 1111001101 | 2200011111 |
| 80 | 90 | 100 | 101 |  |  |  |
| 1111101111 | 1?11110111 | 1111101111 | ? |  |  |  |

*Spinops sternbergorum*

| 10 | 20 | 30 | 40 | 50 | 60 | 70 |
| --- | --- | --- | --- | --- | --- | --- |
| ??1?00???? | ???1-????1 | ??0?000022 | ?111???10? | ????1?1011 | 11?100??01 | 2212000?0? |
| 80 | 90 | 100 | 101 |  |  |  |
| 0????????? | ?????????? | ?????????? | ? |  |  |  |

*Centrosaurus apertus*

| 10 | 20 | 30 | 40 | 50 | 60 | 70 |
| --- | --- | --- | --- | --- | --- | --- |
| 0110000111 | 1001-11111 | 0100000020 | 1111111100 | 0110101011 | 1111001101 | 2212000101 |
| 80 | 90 | 100 | 101 |  |  |  |
| 0111101111 | 1111110111 | 1111101111 | 1 |  |  |  |

*Coronosaurus brinkmani*

| 10 | 20 | 30 | 40 | 50 | 60 | 70 |
| --- | --- | --- | --- | --- | --- | --- |
| 011?000111 | ??01-11111 | 0100001020 | 1111111100 | 0110101011 | 1111001101 | 121??00101 |
| 80 | 90 | 100 | 101 |  |  |  |
| 0111101111 | 1?11?1???? | 111110?111 | ? |  |  |  |

*Xenoceratops foremostensis*

| 10 | 20 | 30 | 40 | 50 | 60 | 70 |
| --- | --- | --- | --- | --- | --- | --- |
| ?????????? | ?????????? | 0????????? | ?????????? | 01?0?010?1 | 11?1??1101 | 1201000101 |
| 80 | 90 | 100 | 101 |  |  |  |
| 01???????? | ?????????? | ?????????? | ? |  |  |  |

*Sinoceratops zhuchengensis*

| 10 | 20 | 30 | 40 | 50 | 60 | 70 |
| --- | --- | --- | --- | --- | --- | --- |
| ??1??????? | ???001???1 | ?????00022 | 010-???100 | 0110100-11 | 1111001111 | 121B000101 |
| 80 | 90 | 100 | 101 |  |  |  |
| 01???????? | ?????1???? | 11?????11? | ? |  |  |  |

*Einiosaurus procurvicornis*

| 10 | 20 | 30 | 40 | 50 | 60 | 70 |
| --- | --- | --- | --- | --- | --- | --- |
| ?11?000??? | ???1-11111 | 0111100022 | 010-111100 | 0110101011 | 1111001100 | ??001A0101 |
| 80 | 90 | 100 | 101 |  |  |  |
| 0111101111 | 1?11?1??1? | 111??1111? | ? |  |  |  |

*Achelousaurus horneri*

| 10 | 20 | 30 | 40 | 50 | 60 | 70 |
| --- | --- | --- | --- | --- | --- | --- |
| 011?000111 | 10?1-11112 | 0111100022 | ?10-1?1100 | 0110101011 | 111100?100 | ??00110101 |
| 80 | 90 | 100 | 101 |  |  |  |
| 0111101111 | ?????????? | ?????????? | ? |  |  |  |

*Pachyrhinosaurus Canadensis*

| 10 | 20 | 30 | 40 | 50 | 60 | 70 |
| --- | --- | --- | --- | --- | --- | --- |
| 0110000111 | 10?1-11112 | 011?110022 | ?10-111100 | ?11010??11 | ???1???100 | ??12110101 |
| 80 | 90 | 100 | 101 |  |  |  |
| 0111101??? | ?????????? | ?????????? | ? |  |  |  |

*Pachyrhinosaurus lakustai*

| 10 | 20 | 30 | 40 | 50 | 60 | 70 |
| --- | --- | --- | --- | --- | --- | --- |
| 0110000111 | 1001-11112 | 0111110022 | ?10-1?1100 | 0110101011 | 1111001101 | ??12110101 |
| 80 | 90 | 100 | 101 |  |  |  |
| 0111101111 | 1?11?1??11 | 111110111? | ? |  |  |  |

*Pachyrhinosaurus perotorum*

| 10 | 20 | 30 | 40 | 50 | 60 | 70 |
| --- | --- | --- | --- | --- | --- | --- |
| 01?0000111 | ???1-11?12 | 01??110022 | ?10-???1?? | ??1??010?1 | 11?1???101 | 121C110101 |
| 80 | 90 | 100 | 101 |  |  |  |
| 01?????1?1 | ???1?????? | ?????0111? | ? |  |  |  |

*Avaceratops lammersi*

| 10 | 20 | 30 | 40 | 50 | 60 | 70 |
| --- | --- | --- | --- | --- | --- | --- |
| 01?0000111 | 10?1-10100 | 011?001111 | ?11011?100 | 0110200-11 | --0100?111 | 0000000101 |
| 80 | 90 | 100 | 101 |  |  |  |
| 011??01111 | 1111?10?11 | ???????1?1 | ? |  |  |  |

*Nasutoceratops titusi*

| 10 | 20 | 30 | 40 | 50 | 60 | 70 |
| --- | --- | --- | --- | --- | --- | --- |
| 0110000111 | 1001-10100 | 011?001111 | ?110??1100 | 0110200-11 | 1101001101 | 1200000101 |
| 80 | 90 | 100 | 101 |  |  |  |
| 01???????1 | 11?1?10??? | ?????????? | ? |  |  |  |

*Wendiceratops pinhornensis*

| 10 | 20 | 30 | 40 | 50 | 60 | 70 |
| --- | --- | --- | --- | --- | --- | --- |
| ?????????? | ???1?101?1 | 0????????? | ?????????? | 01102010?1 | 1111001101 | 1212110100 |
| 80 | 90 | 100 | 101 |  |  |  |
| ?0?????11? | ???1?10?1? | 11???11??? | ? |  |  |  |

*Machairoceratops cronusi*

| 10 | 20 | 30 | 40 | 50 | 60 | 70 |
| --- | --- | --- | --- | --- | --- | --- |
| ?????????? | ?????????? | ????000102 | ????111??? | 0?10101011 | 11??????01 | 22???????? |
| 80 | 90 | 100 | 101 |  |  |  |
| ??1??????? | ?????????? | ?????????? | ? |  |  |  |
